# Cross‐lags and the unbiased estimation of life‐history and demographic parameters

**DOI:** 10.1111/1365-2656.13572

**Published:** 2021-08-18

**Authors:** Martijn van de Pol, Lyanne Brouwer

**Affiliations:** ^1^ Department of Animal Ecology Netherlands Institute of Ecology (NIOO‐KNAW) Wageningen the Netherlands; ^2^ Department of Animal Ecology & Physiology Institute for Water and Wetland Research Radboud University Nijmegen the Netherlands; ^3^ Division of Ecology & Evolution Research School of Biology The Australian National University Canberra ACT Australia

**Keywords:** covariate endogeneity, density dependence, group living, *Malurus elegans*, measurement error, structural equation model, time‐series length, trade‐off

## Abstract

1. Biological processes exhibit complex temporal dependencies due to the sequential nature of allocation decisions in organisms' life cycles, feedback loops and two‐way causality. Consequently, longitudinal data often contain cross‐lags: the predictor variable depends on the response variable of the previous time step. Although statisticians have warned that regression models that ignore such covariate endogeneity in time series are likely to be inappropriate, this has received relatively little attention in biology. Furthermore, the resulting degree of estimation bias remains largely unexplored.
2. We use a graphical model and numerical simulations to understand why and how regression models that ignore cross‐lags can be biased, and how this bias depends on the length and number of time series. Ecological and evolutionary examples are provided to illustrate that cross‐lags may be more common than is typically appreciated and that they occur in functionally different ways.
3. We show that routinely used regression models that ignore cross‐lags are asymptotically unbiased. However, this offers little relief, as for most realistically feasible lengths of time‐series conventional methods are biased. Furthermore, collecting time series on multiple subjects—such as populations, groups or individuals—does not help to overcome this bias when the analysis focusses on within‐subject patterns (often the pattern of interest). Simulations, a literature search and a real‐world empirical example together suggest that approaches that ignore cross‐lags are likely biased in the direction opposite to the sign of the cross‐lag (e.g. towards detecting density dependence of vital rates and against detecting life‐history trade‐offs and benefits of group living). Next, we show that multivariate (e.g. structural equation) models can dynamically account for cross‐lags, and simultaneously address additional bias induced by measurement error, but only if the analysis considers multiple time series.
4. We provide guidance on how to identify a cross‐lag and subsequently specify it in a multivariate model, which can be far from trivial. Our tutorials with data and R code of the worked examples provide step‐by‐step instructions on how to perform such analyses.
5. Our study offers insights into situations in which cross‐lags can bias analysis of ecological and evolutionary time series and suggests that adopting dynamical models can be important, as this directly affects our understanding of population regulation, the evolution of life histories and cooperation, and possibly many other topics. Determining how strong estimation bias due to ignoring covariate endogeneity has been in the ecological literature requires further study, also because it may interact with other sources of bias.

## Graphical abstract

Cross‐lags are omnipresent in biological data. However, we usually ignore them in our statistical analysis. The authors show that this potentially can lead to serious bias for quantifying life‐history trade‐offs, benefits of group living and the strength of density dependence. Multivariate models can account for cross‐lags and can provide a way forward in some cases.

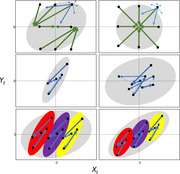

## 1 INTRODUCTION

Avoiding bias is important for making inference about scientific questions, as bias can lead to a systematic misunderstanding of biological processes and to unreliable predictions. Estimation bias can occur when statistical models are misspecified, for example because key confounding variables are not included in the model. Although arguably all models are wrong (Box, 1976), some are more useful than others, and some types of model misspecification may lead to particularly strong biases in estimators such that they profoundly influence biological conclusions. A well‐known example is the analysis of time series that exhibit strong temporal autocorrelation. Statistical models that do not specify the autoregressive nature of the data tend to produce (more) biased estimates of regression coefficients (Keele & Kelly, 2006; Wilkins, 2018).

In addition to auto‐lags, cross‐lags may also occur in multivariate biological time‐series data. A cross‐lag exists when the predictor variable depends on the response variable of the previous time step. An example is the life‐history trade‐off between reproduction (*Y_t_*, e.g. offspring number) and maintenance (*X_t_*, e.g. somatic growth). Any cost of reproduction may generate a negative cross‐lag, as somatic growth (*X_t_*) will then depend on the reproductive success at the previous attempt (*Y_t_*_−1_; Fitzpatrick et al., 2008). As another example: when interested in how reproductive success (*Y_t_*) depends on the population size (*X_t_*; a proxy of competitor density), a positive cross‐lag may be present, as the population size (*X_t_*) typically depends on the reproduction in the previous year (*Y_t_*_−1_).

More generally, cross‐lags in observational time‐series data are caused by the sequential nature of biological systems, or by the fact that the variables of interest often affect each other in multiple ways. Virtually every decision an organism makes will have downstream consequences later in life (Harrison et al., 2011), meaning that when we follow organisms, groups or populations over time (longitudinal data), temporal cross‐dependencies may be likely. In addition, biological systems often exhibit feedback loops over time or two‐way causality (*X* affects *Y*, but *Y* also affects *X*). Thus, cross‐lags are likely to be common in biological time‐series data.

The challenges in analysing cross‐lagged data have received considerable attention from statisticians. Diggle et al. (2002) refer to the challenge that cross‐lags impose as covariate endogeneity: the covariate process is endogenous with respect to the response variable, or in other words, as a situation where the response at time *t* predicts the covariates at times greater than *t*. The problem is that the intricate temporal dynamical relationships caused by cross‐lags may cause statistical non‐independence that is not adequately captured by models that ignore cross‐lags, and hence ignoring cross‐lags may cause biased estimation of parameters of interest. The challenges in analysing cross‐lagged data have also received ample attention in the social sciences (e.g. in studies on how national education level affects economic growth, where growth may also affect future education in longitudinal studies; Solaki, 2013) and medical sciences (e.g. in studies on how anxiety affects depression, where depression may also affect future anxiety of individuals followed over time; Eaton & Ritter, 1988). By contrast, only a few ecological studies touch upon the issue indirectly (Eisenhauer et al., 2015; Hefley et al., 2016; Ives et al., 2003), with only one in‐depth study on the topic (investigating how movement impacts heart rate from high‐frequency tracker data, where heart rate may also affect subsequent movement; Fieberg & Ditmer, 2012). Cross‐lags and its implications thus appear to have received little attention in ecology and evolution.

Here we provide three illustrative examples on classical biological questions in which we think cross‐lags are likely to be important. Using a graphical model, we first intuitively explain why cross‐lags, if ignored, may cause bias in estimating contemporaneous effects of interest (how *X_t_* affects *Y_t_*) in longitudinal studies. We consider both situations when collecting single time series and series on multiple subjects (individuals, groups or populations). We then explore—using simulated and real‐world datasets—the extent of estimator bias by widely adopted static regression models (i.e. models that ignore cross‐lags and wrongly assume that *X* is exogenous instead of endogenous with respect to *Y*; Diggle et al., 2002). We particularly focus on how bias depends on the length and number of time series analysed, as these are typically limited in biology. Furthermore, we show that dynamical multivariate models—such as structural equation models—provide a solution in some cases, and can simultaneously address additional bias induced by measurement error. Finally, we argue that adopting such dynamic models—despite introducing new challenges—could be important for our understanding of fundamental biological questions, as the reliance of the literature on static regression models implies that existing evidence could be biased. The direction of bias due to ignoring cross‐lags is expected to be opposite to the sign of the cross‐lag, which for our examples implies an underestimation of the existence and strength of life‐history trade‐offs and group living benefits and an overestimation of the strength of negative density dependence in vital rates (though in practice other sources of bias may exist in other directions that may also interact).

## 2 THE PROBLEM OF CROSS‐LAGS EXPLAINED BY A GRAPHICAL MODEL

### 2.1 Why cross‐lags cause bias in single time series

**FIGURE 1:**
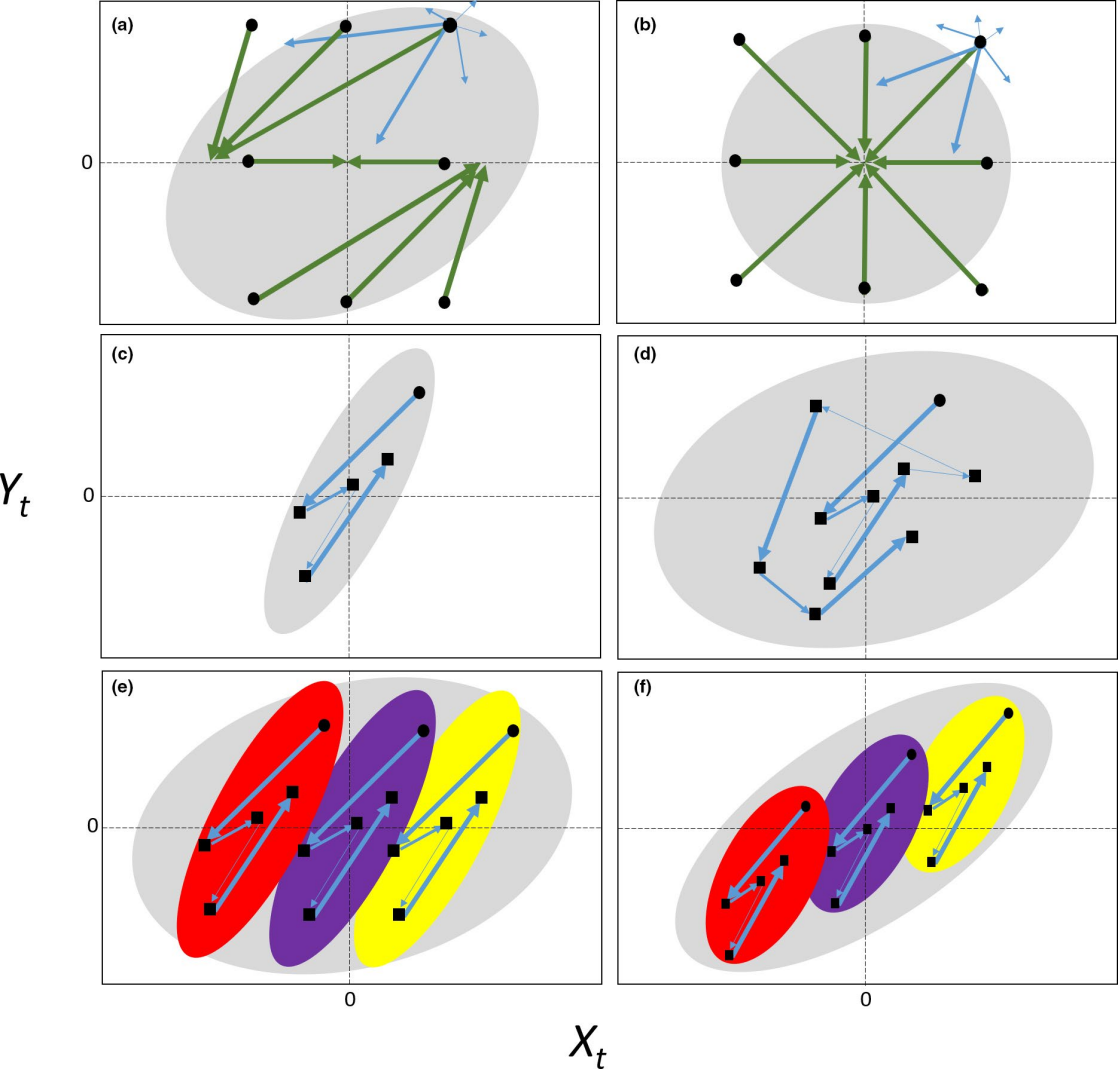
Graphical model illustrating that time‐series data on *X_t_* and *Y_t_* are likely to be correlated when a cross‐lag occurs, even if *X_t_* does not affect *Y_t_*. (a) A negative cross‐lag between *X_t_* and *Y_t_*_−1_ means that if by chance *Y_t_* is higher (lower) than average, then in the next time step both *X_t_*_+1_ and *Y_t_*_+1_ are predominantly expected to be lower (higher), respectively, due to the cross‐lag and regression to the mean. Consequently, a negative cross‐lag causes a positive correlation between $\Delta X_{t\to t+1}$ and $\Delta Y_{t\to t+1}$, which means that datapoints of (*X_t_*, *Y_t_*) are likely to move along the direction of the grey ellipse over time, also causing a directional pattern and correlation between *X_t_* and *Y_t_*. Green arrows originating from black points (*X_t_*, *Y_t_*) depict the most likely temporal trajectory to the observation in the next time step ($\Delta X_{t\to t+1}$, $\Delta Y_{t\to t+1}$), but other less‐likely trajectories are possible, as indicated by thinner blue arrows for the top right datapoint. For comparison, (b) shows an example without cross‐lag (*X* and *Y* being uncorrelated random variables) in which we get regression to the mean for both *X* and *Y*, and we see no directionality (directions of green arrows are diverse, meaning that $\Delta X_{t\to t+1}$ and $\Delta Y_{t\to t+1}$ are uncorrelated). Comparing an example *X*,*Y*‐trajectory over (c) 5 and (d) 10 time steps illustrates how the directional orientation gradually disappears in longer time series, as chance effects cause the variation in *X* to increase over time, which dilutes the directionality caused by the cross‐lag (shallower ellipse in (d) than in (c)). (e) Cross‐sectional patterns (grey ellipse) of multiple time series have little directional orientation, despite each within‐subject pattern (red, purple and yellow ellipses) being directional. (f) However, the cross‐sectional pattern of a heterogeneous population (grey ellipse) often depends on the covariance among‐subjects rather than within‐subject patterns. See main text for additional explanation

Why cross‐lags cause estimator bias in a single time series analysed using static regression models can be intuitively understood from a graphical model (Figure 1). For example, consider the trade‐off between reproduction *Y* and somatic growth *X*, which one could study by measuring reproduction and growth at multiple time steps *t* for a single individual followed over time. If growth *X_t_* does not (or weakly) affects reproduction *Y_t_* (the contemporaneous effect of interest b≈0 in Equation 1a, Box 1), but growth *X_t_* does depend on reproduction in the previous time step $Y_{t‐1}$ due to a cost of reproduction (negative cross‐lag; d<0 in Equation 1b, Box 1), then measurements of (*X*, *Y*) at subsequent time steps are likely to show a specific directionality (green arrows in Figure 1a). The reason is that when considering a point in time with above‐average reproduction ($Y_{t}>0$), growth in the next time step is likely lower due to the high cost of reproduction ($\Delta X_{t\to t+1}<0$), while reproduction in the next time step is also likely to be lower due to regression to the mean ($\Delta Y_{t\to t+1}<0$). Conversely, when considering a point in time with below‐average reproduction ($Y_{t}<0$), growth in the next time step is likely (relatively) higher due to the low cost of reproduction while reproduction in the next time step is likely also higher due to regression to the mean ($\Delta X_{t\to t+1}>0$ & $\Delta Y_{t\to t+1}>0$). Consequently, datapoints of *X*, *Y* are likely to move along the directional grey ellipse in Figure 1a (as $\Delta X_{t\to t+1},\;\Delta Y_{t\to t+1}$ tend to be correlated), even though *X_t_* does not affect *Y_t_* (we assumed b≈0). Such a scenario does not occur when there is no cross‐lag, and regression to the mean for both *X* and *Y* means that there is no directionality (Figure 1b; $\Delta X_{t\to t+1}$ & $\Delta Y_{t\to t+1}$ are uncorrelated). As a result, simply regressing *Y_t_* on *X_t_* time series in the presence of a cross‐lag—that is, fitting a static regression model that assumes that *X* is exogenous, while in fact it is endogenous with respect to *Y*—is likely to suggest that *b* is positive and thereby overestimates its true value (conversely, positive cross‐lag is expected to result in underestimation).

BOX 1 **Equations describing different types of cross‐lagged data structures**

*Data‐generating processes*

We considered four processes for variables *Y*, X (and *Z*) that differ in their cross‐lag and auto‐lag structure. In all examples, the (a) part of the equation describes the process of biological interest while the (b) part describes the cross‐lag process that cause the covariate of interest *X* to be endogenous.

1. Time series of single subject with simple cross‐lagged process: (e.g. trade‐off example: *X* is growth and *Y* is reproduction measured in a single individual at different time steps *t;* a cost of reproduction may cause a simple negative cross‐lag, see Equation 1b. below with *d* < 0).
  - $Y_{t}=a+bX_{t}+\varepsilon_{t}$
  - $X_{t}=c+dY_{t‐1}+\kappa_{t}$
2. Time series of single subject with complex cross‐lag (e.g. density‐dependent example: *X* is population size, *Y* is per capita productivity and *Z* survival measured in a single population; an interacting positive cross‐ and auto‐lagged process occurs, as population size in year *t* depends on the per capita productivity (& survival) multiplied by the previous population size, see Equation 2b).
  - $Y_{t}=a+bX_{t}+\varepsilon_{t}$
  - $X_{t}=c+\left(dY_{t‐1}+fZ_{t‐1}\right)X_{t‐1}+\kappa_{t}$
  - $Z_{t}=g+\lambda_{t}$
3. Time series of multiple subjects (*s*) with complex cross‐lag and among‐subject covariance (e.g. benefits of group living example: *X* is group size, *Y* is group productivity and *Z* survival measured on many groups; a positive cross‐ and auto‐lagged process occurs as group size in year *t* depends on the group productivity and group size in the previous year, see Equation 3b).
  - $Y_{s,t}=a+bX_{s,t}+\mu_{s}+\varepsilon_{s,t}$
  - $X_{s,t}=c+dY_{s,t‐1}+fZ_{s,t‐1}X_{s,t‐1}+\kappa_{s,t}$
  - $Z_{s,t}=g+\nu_{s}+\lambda_{s,t}$
4. Time series of multiple subjects with a simple cross‐lagged process and among‐subject covariance (e.g. trade‐off example, same as example 1 but now considering multiple individuals/subjects *s*).
  - $Y_{s,t}=a+bX_{s,t}+\mu_{s}+\varepsilon_{s,t}$
  - $X_{s,t}=c+dY_{s,t‐1}+\nu_{s}+\kappa_{s,t}$

Parameters *a* through *g* represent constants, *b* being the contemporaneous effect of interest to be estimated accurately and *d* the cross‐lag parameter. Random normal variables $\varepsilon_{s,t}$, $\kappa_{s,t}$, $\lambda_{s,t}$ reflect uncorrelated (white) noise, for example due to environmental stochasticity. Multivariate correlated random variables $\mu_{t}$, $\nu_{t}$ describe among‐subject (co)variation due to, for example, quality differences. Note that Equation 4 includes among‐subject covariance in *X* and *Y*, while Equation 3 includes a covariance between *Y* and *Z*, which ultimately also causes *X* and *Y* to be correlated among subjects.

*Simulating datasets*

We generated simulated datasets based on the processes described in Equations 2–4 to reflect the biological examples of density dependence, group living and trade‐offs. Response variables *X*, *Y* and *Z* were sampled across *s* subjects and *t* time step. We assumed that all response variables were generated by Gaussian processes, though we acknowledge that in reality they are typically generated by discrete processes (e.g. Bernoulli for survival, or Poisson‐like process for reproduction or group size). However, this simplification to the Gaussian case is useful here, as (a) it suffices to illustrate our point about bias and (b) it means that we can use more conventional statistical packages (e.g. lavaan; Rosseel, 2012) to analyse these datasets in *R*, as illustrated in Tutorial 1. In Section 7, we provide an example and Tutorial with Poisson and binomial variables on a more realistic real‐word case study. We also acknowledge that other (confounding) variables may need to be included in real‐world studies, but we ignore these here as they are not needed to make our point. Random normal variables $\varepsilon_{s,t}$, $\kappa_{s,t}$ and $\lambda_{s,t}$ were thus modelled as Gaussian noise, for example, $\varepsilon_{s,t}=N(0,\;\sigma_{\varepsilon }^{2}$). Among‐subject (co)variation was modelled using multivariate correlated random variables ${\left(\mu_{t},\;\nu_{t}\right)}^{T}=\text{MultivariateNormal}\left(0,\;\Omega_{\mu ,\nu }\right)$.

We first varied the number of subjects (1, 10, 100 or 1,000) and time‐series length (5, 10, 20, 40 or 80) to explore how bias depends on these variables. These values were chosen to reflect that most studies do not follow populations, groups or individuals for long, as study duration rarely exceeds 10–20 years and individuals or groups die after a limited number of years; while longer time series were considered to further explore how any bias depends on series length. In these simulations, we assumed a fixed level of effect size, cross‐lags and positive among‐subject covariance (values shown by vertical reference lines in Figure 3). Furthermore, to explore how estimation bias depends on the strength of cross‐lag, among‐subject covariance and effect size, we also generated datasets with varying values for parameters *b*, *d* and *σ_µ_*_,_ *_ν_* for a situation of short time series (10 time steps) and either a single (1) or multiple (100) subjects.

We created up to 50,000 replicates of simulated datasets for each set of parameter combinations and describe the bias in estimates of *b* averaged among replicates (Tutorial 1 for R code & parameter values; Brouwer & van de Pol, 2021).

Figure 1a illustrates why cross‐lags may cause *X_t_* and *Y_t_* to be correlated, even if *X_t_* does not causally affect *Y_t_*. However, with increasing length of time series, we gradually get more movement along the *X*‐axis over time (Figure 1c vs. 1d). This increase in variance of *X* with time‐series length causes the overall scatter of *X*, *Y* datapoints to be less directional (shallower grey ellipse in Figure 1c vs. 1d) and consequently a static regression approach is expected to produce less (positively) biased estimates of contemporaneous effect *b* in long compared to short time series (converging to the true value, here *b* = 0, for infinitely long time series; see Section 4). The reason for the predicted increase of variance in *X* with time‐series length (Figure A in Supplementary Material A) is that due to chance (i.e. residual variation in *X*) there will be more and more changes over time in directions opposite to the direction caused by the cross‐lag. In conclusion, we expect that static regression models provide biased estimates of contemporaneous effects of interest only for short cross‐lagged time series. An outstanding question is whether this bias is likely to be strong for the lengths of time series that are typically achieved in ecological and evolutionary studies (often time series are particularly short for traits measurable once a year [e.g. reproduction], as then time‐series' length is constrained by a species life span [when following individuals] or study duration [when following a population]).

### 2.2 Why cross‐lags cause bias when collecting multiple time series

In biology, we often collect and jointly analyse time series on many different subjects (e.g. multiple individuals, groups or populations). We graphically illustrate the impact of cross‐lag in such situations by again considering the trade‐off between reproduction and somatic growth, but now assume that we have measured both variables over time for multiple individuals. Cross‐sectional patterns of multiple short time series typically also cover a wider range of *X*‐values (due to chance and among‐subject heterogeneity in *X*) than a single time series, and thus have little directional orientation, despite each within‐subject pattern being directional (indicated by the coloured ellipses of three subjects followed over a short time in Figure 1e, which together determine the cross‐sectional grey ellipse). The joint (cross‐sectional) analysis of multiple short cross‐lagged time series can thus in theory reduce bias in the contemporaneous effect of interest in the same way as collecting longer single time series can (Figure 1e vs. 1d).

However, collecting more instead of longer time series may not necessarily—possibly rarely—offer a practical solution for biological studies. The reason is that subjects typically differ systematically in the amount of resources they acquire, which causes a positive among‐subject covariance between *X* and *Y* (Reznick et al., 2000; van Noordwijk & de Jong, 1986). For example, some individuals consistently both grow and reproduce faster than others because their territory has more resources or they are better foragers. The issue is that studies typically do not hypothesize about such among‐subject patterns, but instead hypothesize specifically about within‐subject patterns (e.g. whether high growth causes lower reproduction; Dingemanse & Dochtermann, 2013; Nussey et al., 2007; van de Pol & Wright, 2009). Classical cross‐sectional comparisons confound the within‐subject patterns of interest (e.g. the individual's life‐history trade‐offs) with the among‐subject patterns that are not of primary interest (e.g. driven by heterogeneity in individual or habitat quality; Snijders & Bosker, 1999; van de Pol & Wright, 2009). This is illustrated in Figure 1f where the overall cross‐sectional pattern (orientation of the grey ellipse) is primarily influenced by how the coloured ellipses of subjects are non‐randomly clustered across the *X*,*Y*‐plane (due to the positive among‐subject covariance in *X* and *Y*), and little influenced by the within‐subject pattern (which could even have had the opposite direction/sign).

To avoid committing an ‘ecological fallacy’ (Selvin, 1958; Simpson, 1951), most biological observational studies that hypothesize about within‐subject mechanisms nowadays focus on estimating the average within‐subject association across all subjects (Dingemanse & Dochtermann, 2013; van de Pol & Wright, 2009). However, this within‐subject focus reintroduces the directional bias caused by cross‐lags again, because it effectively shifts focus from the grey (*X_t_*, *Y_t_*) ellipse to the coloured (*X_s_*_,_ *_t_*, *Y_s_*_,_ *_t_*) ellipses in Figure 1f, which, in turn, reflects the situation of Figure 1c that exhibits bias. Consequently, the biological necessity of studying within‐subject associations in heterogeneous systems (to avoid an ecological fallacy) implies that collecting data on multiple subjects is not expected to have the same benefit for reducing estimation bias compared to increasing time‐series length. Analysing multiple short time series with cross‐lags using static regression methods is thus expected to result in biased estimation too, if the analysis aims to test a hypothesis that reflects within‐subject patterns. Hence, also in such situations an outstanding question is whether this bias is likely to be strong for the lengths of subjects' time series that are typically achieved in ecological and evolutionary studies (Sections 3 and 4), and what statistical models could be used to avoid any potential bias (Section 5).

## 3 BIOLOGICAL EXAMPLES OF CROSS‐LAGS

We will now introduce three biological examples that likely exhibit cross‐lags and typically deal with relatively short time series. In Section 4, we will then use these examples to simulate cross‐lagged datasets with known effect sizes to quantitatively confirm the prediction from the graphical model that static regression models results in estimation bias. One example deals with a situation of analyses on a single time series (Section 3.1) while the other two reflect situations in which time series on multiple subjects are collected, but where the interest is on unbiased estimation of within‐subject patterns (Sections 3.2 and 3.3). These examples differ in the way in which cross‐lags occur and illustrate some of the ecological and evolutionary questions that encounter cross‐lags, but more examples likely exist.

### 3.1 Biological example: Density dependence of vital rates

The first example deals with cross‐lags that occur when the predictor variable itself is an explicit function of the dependent variables. Specifically, we consider the study on density dependence which aims to quantify the effect of population density (*X_t_*) on demographic vital rates (*Y_t_*; e.g. reproduction or survival, or traits or fitness components strongly associated with vital rates). Observational studies on density dependence often follow a single population over time for relatively short periods, typically determined by the number of years a population is studied in the case of annually reproducing species (Salguero‐Gómez et al. (2015, 2016) suggest that demographic studies used for population modelling span on average 11 years for animals (87% of studies <20 years) and 6 years for plants (99% <20 years)). In the case of an iteroparous species living in a population with limited dispersal, the population size equals the sum of the per capita reproductive and survival rate times the previous population size (Equation 2, Box 1). This means that a positive cross‐lag is expected because population size *X_t_* will depend on vital rate *Y_t_*_−1_ (in populations with dispersal, a cross‐lag may still occur, it will just be weaker).

The issue of cross‐lags has received no previous attention in the context of analysing observational time series on vital rates and population density, which is striking given the well‐established literature on the challenges that temporal dependencies cause for accurate estimation in the context of estimating density dependence of population growth or size (Freckleton et al., 2006; Maelzer, 1970; St. Amant, 1970). In fact, when reviewing the analysis of density dependence, Lebreton and Gimenez (2013) state that ‘*contrary to methods based on population* [growth and] *size, the presence and intensity of density is not overestimated*’ when using static regression models in studies on vital rates, and conclude that ‘*the assessment of density dependence based on traits* [such as vital rates] *is relatively straightforward*’. Their assessment, however, did not consider any potential influence of ignoring cross‐lags on the estimation accuracy of density dependence of vital rates. Moreover, using static regression to analyse density dependence in vital rates appears to be the norm: a literature search indicated that none of the nearly 3000 studies on this topic mentioned the terms ‘covariate endogeneity’ or ‘cross‐lag’, and focusing on 10 recent studies showed that all of them regressed vital rates on population size without accounting for any covariate endogeneity (Supplementary Material B).

### 3.2 Biological example: Benefits of group living

The second example considers studies on the evolution of cooperation or group living, which often focus on how group size affects fitness components (e.g. group productivity and survival). It is similar to the first example, but one key difference with studies on density dependence of vital rates is that behavioural ecologists typically follow many groups over time and thus analyse multiple time series. Positive cross‐lags may be expected in studies on group living because fitness components also determine group size in the next time step. Specifically, studies on cooperative breeding typically investigate how group size (*X_t_*) affects a group's total reproductive success (*Y_t_*). However, when offspring delay their dispersal to stay and help raise the next brood (Koenig & Dickinson, 2016), group size itself will directly depend on the reproductive success of previous years ($Y_{t‐1}$; Equation 3, Box 1).

In studies of cooperative species, it is challenging to implement meaningful experimentation, because manipulation of group size often has undesired side effects (Cockburn, 1998). Consequently, many studies rely on quantifying the cost and benefits of group living using longitudinal observational data (Majolo et al., 2008), with time‐series length constrained by life span of groups or study length (thus being relatively short). It has been widely acknowledged that among‐group variation in habitat quality may confound cross‐sectional associations, as high‐quality habitat could allow for some groups to have consistently large size and high reproduction, even if group size does not affect reproduction (Brown, 1978). This realization has led to the use of within‐group comparisons. Such ‘paired’ comparisons, in turn, have been criticized, as it has been suggested that groups that change size are a biased sample of the population (Dickinson & Hatchwell, 2004, but see Cockburn et al., 2008).

Interestingly, it has been acknowledged that the direction of causality can be two way: group size not only affects reproduction, but reproduction also affects group size (Cockburn, 1998). However, the implications of cross‐lags for statistical estimation have, to our knowledge, never been explored in the context of group living. Furthermore, a literature search indicated that none of the over 3000 studies on this topic mentioned the terms ‘covariate endogeneity’ or ‘cross‐lag’. And a focus on 10 recent studies that quantified the costs or benefits of group living on a fitness component using time‐series data showed that all of them regressed fitness components on group size while ignoring possible covariate endogeneity (Supplementary Material B).

### 3.3 Biological example: Life‐history trade‐offs

Finally, the third example considers the previously described scenario of a life‐history trade‐off, where a negative cross‐lag is likely due to organisms having to make sequential choices for recurring events during their lifetime on how to allocate limited resources. Evolutionary ecologists typically collect data on multiple individuals, and among‐individual covariance between growth and reproduction can be expected (e.g. caused by heterogeneity in individual quality; equation 4, Box 1). An alternative biological representation of such a simple cross‐lag structure, but one that involves positive cross‐lag, may occur from two‐way causality or a feedback loop. For example, when body size (*X_t_*) increases dominance (*Y_t_*) and high dominance (*Y_t_*), in turn, leads to large body size (*X_t_*_+1_; e.g. due to better access to food; Fitzpatrick et al., 2008). Notably, the length of time series on individuals is constrained by their life spans, which from a statistical perspective is very short in most species (e.g. the generation time across all mammal species has a median of 3 years and rarely exceeds 10 years; Pacifici et al., 2013).

### 3.4 Cross‐lags come in different forms

The above examples illustrate the diversity in biological questions in which cross‐lags can play a role. However, it should be noted that not only the sign of the cross‐lags but also the structure of the cross‐lags varies slightly among these examples. Specifically, when comparing the equations in Box 1, it becomes clear that data may exhibit simple cross‐lag in the case of trade‐offs ($X_{t}∼Y_{t‐1}$; Equation 4b), cross‐lag as well as auto‐lag in *X* in the case of group‐size studies ($X_{t}∼Y_{t‐1}+X_{t‐1}$; Equation 3b) and a cross‐lag that depends on the auto‐lag in the case of density dependence of vital rates ($X_{t}∼Y_{t‐1}\times X_{t‐1}$; Equation 2b). Finally, we note that Fieberg and Ditmer's (2012) example on movement and heart rates is structurally intermediate to our trade‐off and group living example, but that tracker/logger data typically involve rather long time series and thus the bias we focus on here may be less relevant in such situations (and for high‐frequency movement or physiological data more generally).

## 4 ESTIMATOR BIAS IN STATIC REGRESSION MODELS FOR SIMULATED DATA EXAMPLES

To determine to what extent static regression models result in biased estimates of the contemporaneous effect of interest *b*, we generated simulated datasets reflecting the above three biological examples (based on Equations 2–4 in Box 1). In addition, we explored how various relevant factors further affected this bias, by (a) varying either time‐series length and/or the number of subjects being measured, for a given level of the contemporaneous effect of interest, cross‐lag and among‐subject covariance, or by (b) varying the level of the contemporaneous effects of interest, cross‐lag and (positive) among‐subject covariance, for a given time‐series length and number of subjects (see Box 1 & Tutorial 1 for details, R code and parameter values). In our simulations, we used the most simplified representation of above biological examples, as they are sufficient to illustrate the way that bias may occur in such cases (discussed in Box 1); real‐world studies will likely be more complex—involving different distributions and other confounding processes—but we expect the same principles to apply there. We show that by applying conventional static regression models that either focus on overall (cross‐sectional) patterns or on within‐subject patterns to the simulated data, the graphical predictions from Figure 1 about bias in the estimator of the contemporaneous effect of interest are confirmed in all three examples (see next two subsections).

### 4.1 Single time series

**FIGURE 2:**
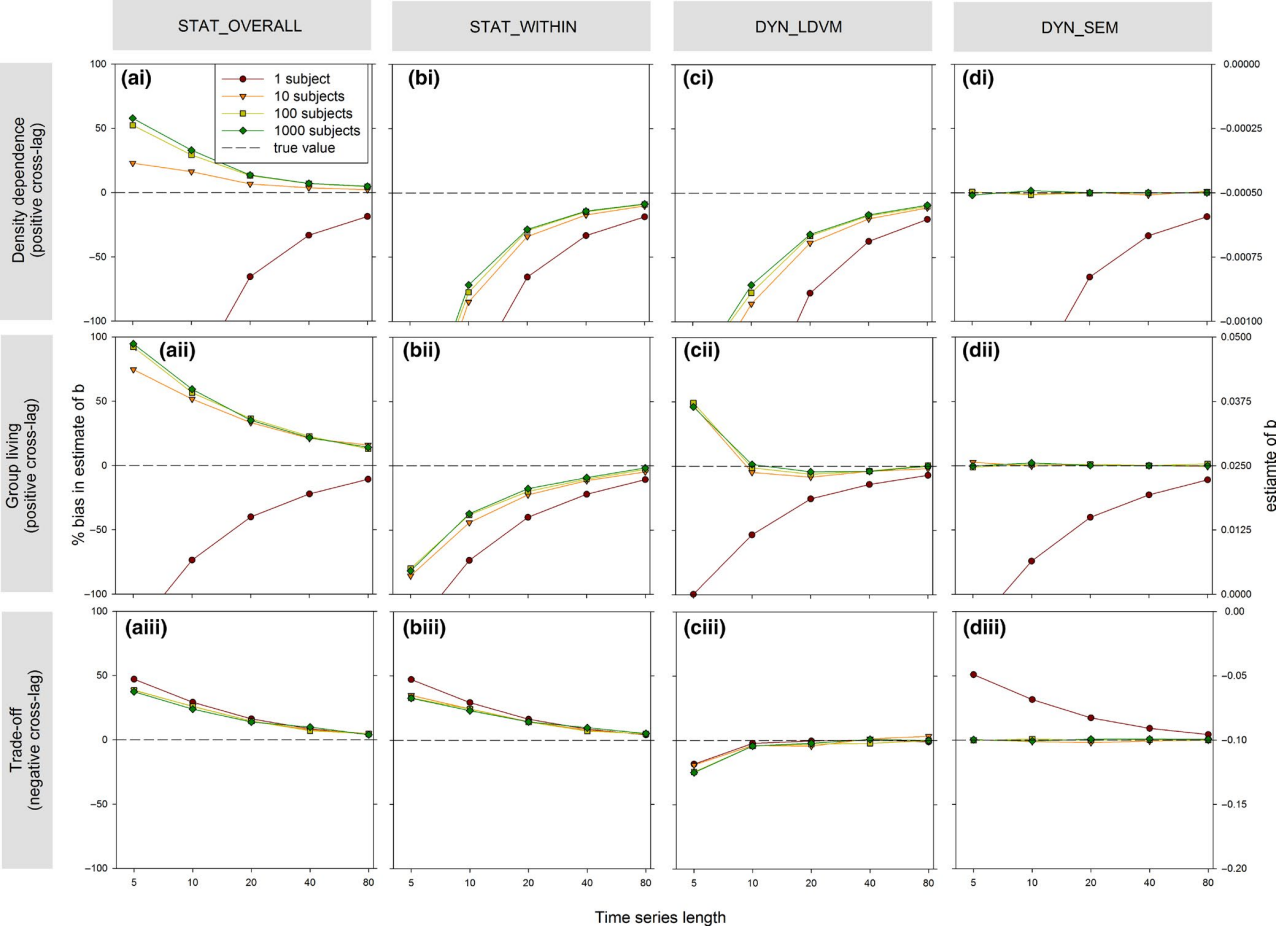
The (bias in) estimates of parameter of interest *b* (contemporaneous effect of *X_t_* on *Y_t_*) as a function of time‐series length determined by (a, b) static and (c, d) dynamical regression models applied to simulated cross‐lagged data with varying number of subjects (see legend). Panel rows reflect situations of (i) density dependence of vital rates, (ii) benefits of group living and (iii) trade‐offs. For each situation, we considered analyses of single time series (e.g. density dependence in a single population) as well as analyses of multiple time series (10, 100 or 1,000 subjects) in the presence of among‐subject covariance (see Boxes 1 and 2). Note that the *x*‐axes are logarithmic and that the left and right *y*‐axes show, respectively, the relative bias and absolute value of the estimate of *b* for each panel

In a situation reflective of a single time series (density‐dependent example), the static linear regression model (Box 2) provided negatively biased estimates of *b* for short time series (red lines in Figure 2a‐i & b‐i; for a single time‐series STAT_OVERALL and STAT_WITHIN are equivalent models). This was consistent with the prediction of our graphical model because the density‐dependent example reflects a scenario of a positive cross‐lag (rather than a negative cross‐lag as depicted in Figure 1a). Our simulation results highlight that this bias can potentially be substantial, particularly for time‐series lengths that are typically achieved in empirical studies (e.g. bias of >60% if ≤20 time steps/years; Figure 2a‐i). However, the bias gradually reduced in long time series (≥80 time steps; Figure 2a‐i), consistent with the graphical prediction that the bias disappears asymptotically with series length (due to the variance of *X_t_* also increasing and levelling off over time in cross‐lagged data, Figure A in Supplementary Material A).

BOX 2 **Static and dynamical statistical models used for parameter estimation**

We applied two static (STAT) and two dynamical (DYN) models to estimate the contemporaneous effect of *X_t_* on *Y_t_* (i.e. parameter *b* in Equations 2–4, Box 1). In addition to a conventional cross‐sectional static mixed model (STAT_OVERALL, Figure Box 2a), we also considered a static model that aims to filter out the masking effect of any among‐subject covariance in *X_t_* and *Y_t_* (i.e. $\sigma_{\mu ,\nu }>0$ in Equations 3 and 4, Box 1) on the estimation of parameter *b* (STAT_WITHIN, Figure Box 2b). To achieve this, we used a technique called within‐subject centring, which is widely used in analyses of longitudinal data of multiple subjects (Snijders & Bosker, 1999; van de Pol & Wright, 2009). This technique removes any among‐individual variation from the predictor variables *X* by subtracting the subject's mean value $\bar{X_{S}}$ from the original $X_{s,t}$ values. Analysing the time series of each subject separately using a simple static model and then taking the mean regression coefficient across all subjects gives similar outcomes as for STAT_WITHIN.

We implement a dynamical model with a lagged‐dependent variable (DYN_LDVM), which does not explicitly model the cross‐lagged dependencies in the data, but accounts for the autocorrelation in *Y_t_* that the cross‐lag causes, by including *Y_t_*_−1_ as a lagged‐dependent variable (Figure Box 2c). The DYN_LDVM also includes a random intercept term for subjects that accounts for systematic differences among subjects in *Y*; therefore, we also used within‐subject centring to the lagged *Y*‐term to assure it accounts for any within‐subject temporal dependency in *Y* caused by the cross‐lag. Finally, a multivariate dynamical model was implemented using structural equation models (DYN_SEM) depicted in Figure Box 2d‐i to d‐iii that specifically incorporates (a) the underlying cross‐lag structure between *X_t_* and *Y_t_*_−1_ as well as any auto‐lags present in Equations 2–4 (Box 1), respectively, and (b) correlated random intercept terms that describe how variables covary among subjects (e.g. covariance between *X* and *Y* is modelled by *σ_µ_*_,_ *_ν_* in Figure Box 2d‐iii). The correlated random intercept terms are crucial in allowing for the cross‐lag in the regression equation for *X_t_* to influence the estimation of the contemporaneous effect in the regression equation of *Y_t_*, as these are the only shared parameters between the two regression equations (Figure Box 2d‐iii). Therefore, when no correlated random intercepts are or can be included, which is the case when analysing a single time series, the regression equation of *Y_t_* of the DYN_SEM is identical to that of the STAT_OVERALL and gives the same estimate for the contemporaneous effect of interest.

The STAT_OVERALL, STAT_WITHIN and DYN_LDVM models were implement using the r packages lm and lme4 (Bates et al., 2015) while DYN_SEM was implemented using the r package lavaan (frequentist; Rosseel, 2012) and rstan (Bayesian; Guo et al., 2016), code in Tutorial 1 (Brouwer & van de Pol, 2021).

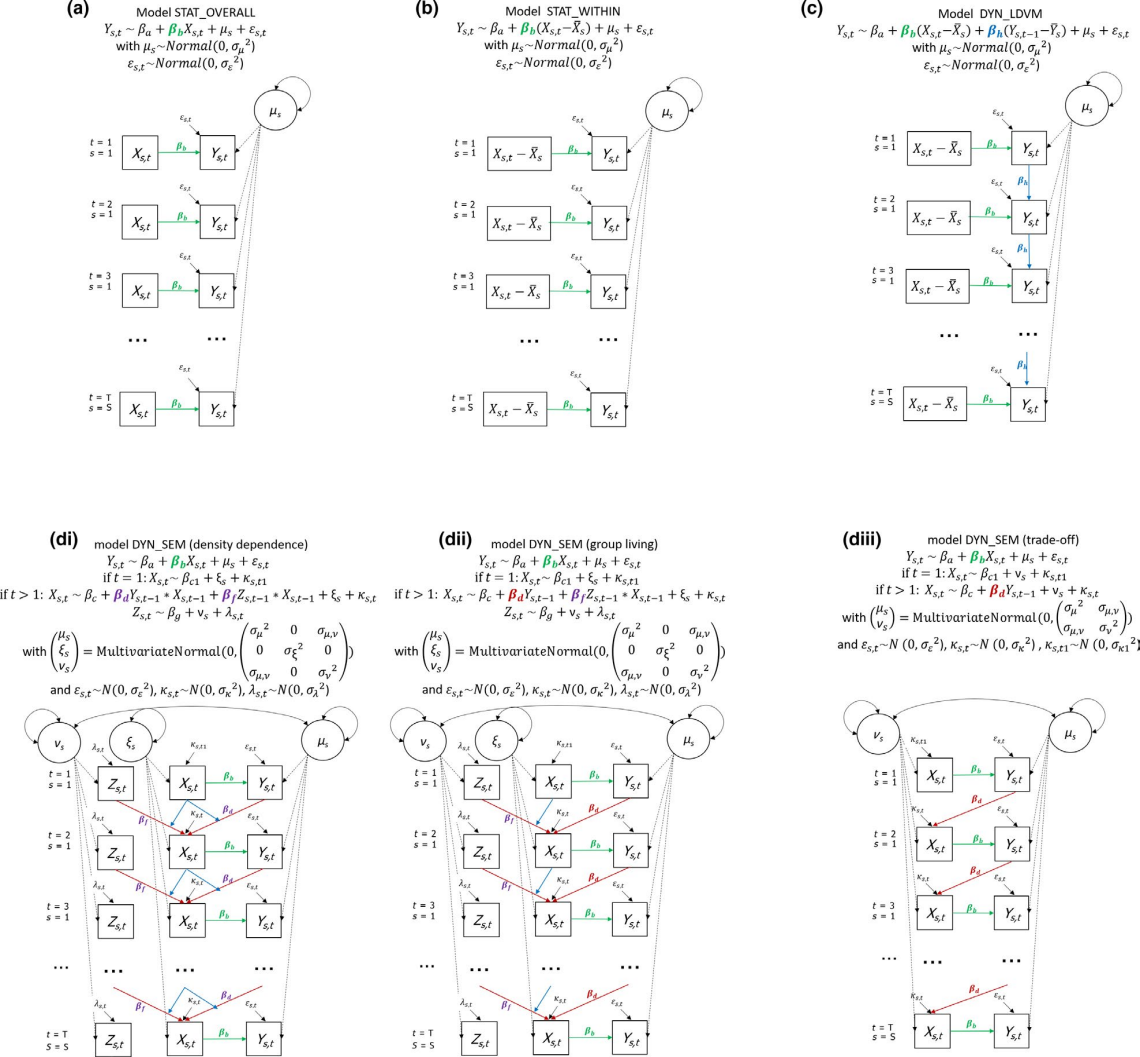

FIGURE BOX 2: Regression equations and graphical depiction (path diagrams) of models used to analyse the different example datasets (Equations 2–4, Box 1). The estimate of interest $\beta_{b}$ is highlighted in green. The parameter $\beta_{d}$ estimates the cross‐lag between $X_{t}$ and $Y_{t‐1}$ (in red). Some models also included an auto‐lag (in blue) between $Y_{t}$ and $Y_{t‐1}$, or an interactive effect of both cross‐ and auto‐lags (in purple). Circles represent (latent) random intercept variables that account for non‐independency among measurements of the same subjects. Two‐way arrows reflect correlated terms. When applying models to a single time series, all random intercept terms for subjects and correlations among them were dropped

### 4.2 Multiple time series

**FIGURE 3:**
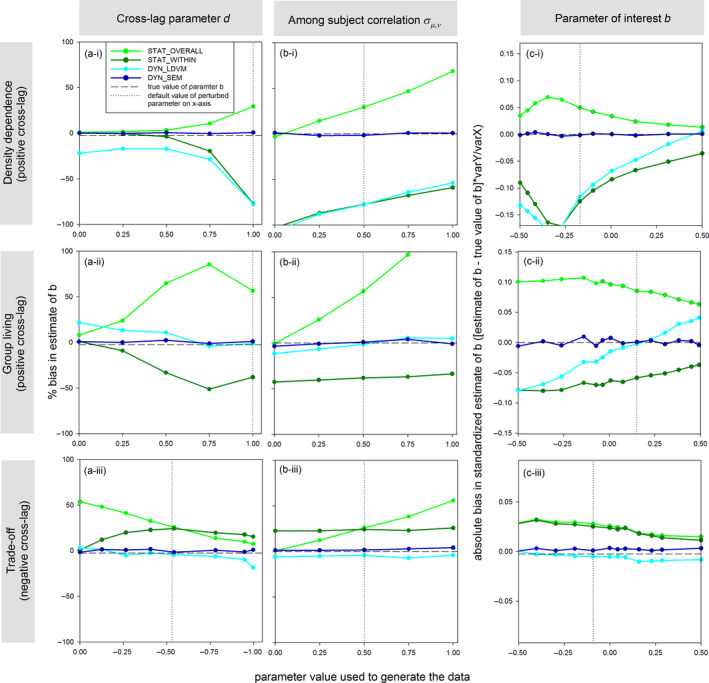
The sensitivity of estimation bias in parameter of interest *b* to (a) the strength of the cross‐lag, (b) the amount of among‐subject covariance and (c) the strength of the contemporaneous effect size *b*, when using static and dynamic regression models (see legend) for all three example scenarios (row panels i–iii). The vertical dotted lines show the default value of the parameters used in other simulations and figures. Note that values of parameters *b* and *d* are shown in standardized units obtained by multiplying them with var*Y*/var*X* and var*X*/var*Y*, respectively (except in panels a‐i & a‐ii, as the absolute value of *d* can be interpreted as the probability of natal philopatry/emigration). For panels (a) and (b), we calculated the relative bias, while for panel (c) we present the absolute bias (as relative bias does not exist for *b* = 0). All results are the mean across estimates on 1,000 simulated datasets of multiple (100) subjects and 10 time steps, see Figure D in Supplementary Material D for single time‐series datasets

As graphically predicted in Figure 1, static methods also showed biased estimation of *b* when multiple time series (subjects) are analysed simultaneously. The within‐subject focus of STAT_WITHIN caused negative bias in situations of positive cross‐lag (group living; Figure 2b‐ii) and positive bias in situations of negative cross‐lag (trade‐off; Figure 2b‐iii) for short time‐series lengths, similar to the previous analysis of single time series. Furthermore, in the cross‐sectional static analysis (STAT_OVERALL), a positive among‐subject covariance between *X* (e.g. growth) and *Y* (e.g. reproduction) is expected to mask any within‐subject relationship/trade‐off (see Figure 1f), and consequently estimates were too high in the presence of among‐subject covariance (Figure 2a). The bias of STAT_OVERALL and STAT_WITHIN both increased with the strength of the cross‐lag (Figure 3a) and, respectively, increased and decreased with the amount of among‐subject covariance (Figure 3b) while dependencies on effect size showed complex and variable patterns (Figure 3c).

Notwithstanding the observation that bias from static models is primarily relevant for short(ish) time series, biased estimation does not only occur when sample size (and statistical power) is low. For example, strong bias from static methods was apparent in short series of many subjects (e.g. 5 time steps of 1,000 subjects; Figure 2a,b) while such situations reflect study designs with large sample sizes and very high power (i.e. static estimates of parameter *b* were statistically significant at the 0.05 threshold level in all such simulated datasets, suggesting power of ~100%). The relationship between bias, statistical power and study design thus appears highly contextual. Bias depends on time‐series length while power depends on both the number of subjects and time‐series length (and the signal‐to‐noise ratio of the true effect size). Our simulation code provides researchers with a tool to perform power analyses tailored to their specific study system (see Tutorial 1, including for the dynamical models described in the next section).

## 5 A POTENTIAL SOLUTION: DYNAMICAL REGRESSION MODELS

The fact that static regression models did not adequately estimate effect sizes in short and sometimes even in quite long cross‐lagged datasets is likely due to its failure to capture the dynamical properties of the underlying data‐generating process. Dynamical regression models, such as lagged‐dependent variable models (Keele & Kelly, 2006) or multivariate models, may provide a more suitable alternative. Lagged‐dependent variable models (DYN_LDVM, Box 2) are univariate models that do not explicitly model the cross‐lagged dependencies in the data, but aim to account for the autoregressive nature of the dependent variable caused by the cross‐lag, via the inclusion of a lagged response variable as a predictor. By contrast, multivariate models allow for explicitly modelling the cross‐lagged mechanism underlying the data while also explicitly modelling any among‐subject covariance among variables (e.g. between *X* and *Y*). Here we adopt structural equation models (DYN_SEM, Box 2) and its associated terminology and estimation framework (Grace, 2006; Shipley, 2016), as this is arguably the multivariate framework most familiar to ecologists. In Section 8, we discuss similarities and differences with other multivariate time‐series modelling frameworks for cross‐lagged time series on multiple subjects (cross‐lagged panel models, Mund & Nestler, 2019; vector autoregressive models and state‐space models, de Valpine, 2002; Holmes et al., 2012).

### 5.1 Performance of dynamical regression models on simulated cross‐lagged data

Dynamical models outperformed the static models in terms of accuracy on all three simulated datasets, except when analysing a single time series (equally biased as static models) or when there was little among‐subject covariance (equally unbiased as static models). When analysing multiple time series, the multivariate DYN_SEM provided unbiased estimates of *b* in all three biological examples even for very short time series (Figure 2d). This unbiased estimation by DYN_SEM held over the entire parameter space explored (Figure 3). Both the cross‐lag and among‐subject covariance are important to jointly include in the DYN_SEM, as excluding either of these terms from the DYN_SEM causes the estimates for the contemporaneous effects to become biased estimates in the same way as the static models (Supplementary Material C). Contrasting the case of multiple series, for single short time series, the DYN_SEM resulted in a large bias (Figure 2d, similar to the static methods), which is unsurprising as for a single time series the regression equation for *Y_t_* in the DYN_SEM is equivalent to both static models (see Box 2).

The DYN_LDVM generated strongly downward biased estimates in the density‐dependent example (~50% bias for single series of length 20; Figure 2c‐i). The DYN_LDVM appeared to be quite unbiased in the group living example (Figure 2c‐ii), but further sensitivity analysis showed that bias was larger for weaker cross‐lags (Figure 3a‐ii) and stronger effect sizes (Figure 3c‐ii). Strikingly, the DYN_LDVM performed unbiased for the trade‐off example as long as time series were longer than five time steps (Figure 2c‐iii). This high accuracy held across the entire explored parameter space when analysing either multiple (Figure 3) or single time series (Figure D in Supplementary Material D). We hypothesize that the poor(er) performance of the DYN_LDVM in the density‐dependent and group living example is caused by the fact that the cross‐lag between *X_t_* and *Y_t_*_−1_ is moderated by *X_t_*_−1_, while this is not the case in the simpler trade‐off example (Equations 2 and 3 vs. 4, Box 1). Possibly, a complex lag‐structure is not well accounted for by the lagged‐dependent variable $Y_{t‐1}$, and thus bias remains.

In conclusion, only the DYN_SEM models performed well in all examples with multiple time series (Figure 2d). This could be viewed as a trivial result, because the DYN_SEM structures were specified such that they reflected the underlying causal cross‐ and auto‐lag as well as among‐subject (co)variance structure used to generate the data in each example (Boxes 1 and 2). Notwithstanding, the unbiased performance of DYN_SEM is insightful in three ways. First, it shows that DYN_SEM provides accurate estimates even when time series are very short, as long as there are multiple subjects (e.g. green lines at 5 time steps in Figure 2d). This is not trivial, because in some examples these multivariate mixed models included quite complex patterns of temporal cross‐ and auto‐lag and are thus expected to be data hungry. Second, the fact that DYN_SEM performed unbiased with multiple time series, while the DYN_LDVM and static models did not, highlights a novel point: in addition to knowledge about the underlying causal pathways, some multivariate models also required an additional variable to be modelled. Specifically, DYN_SEM was the only model that consistently provided unbiased estimates of group living (Figures 2 and 3), but at the same time it was also the only model that included a third variable *Z* (survival rate; Box 2) in addition to the reproduction and group‐size variables. Thus, this may imply that to obtain unbiased estimates of the reproductive benefits of group living (in situations of multiple shortish time series that exhibit among‐subject covariance), survival data are required and be modelled explicitly, thereby setting additional demands on data collection (similarly, unbiased estimation of survival benefits of group living may require reproduction data). Third, analysing single time series with dynamical models did not produce unbiased estimates, which may be particularly worrisome for studies on density dependence of vital rates, as these typically only analyse a single time series. This suggests no accurate method may yet exist for such cases, though bias was only strong when cross‐lags were strong (Figure 3a‐i) and very long time series are expected to have relatively little bias (>80 time steps may be achievable for some multivoltine species).

### 5.2 Risk of misspecifying the cross‐lag structure

The problem of estimation bias in short time series due to misspecification of cross‐lags in statistical models varied among examples and appeared to depend on the degree of misspecification. The static models always performed poorly, implying that including either an auto‐ or cross‐lag term is needed to account for the autocorrelated nature of the dependent variable caused by the cross‐lag (as well as correlated random intercept terms that allow the cross‐lag in the regression equation of *X_t_* to influence the estimation of the contemporaneous effect in the regression equation of *Y_t_*). The DYN_LDVM estimator was not biased in the trade‐off example (Figure 2c‐iii), although it only included an auto‐lag term, implying that in some cases cross‐lags may be adequately accounted for by auto‐lag terms. However, misspecification of the underlying cross‐lag structure appeared to be quite problematic in the other examples. For instance, in the density‐dependence example, the DYN_LDVM produced biased estimates (Figure 2c‐i). Regrettably, we lack a general understanding of when misspecification of the cross‐lag structure is problematic for estimation (and in what situations including a lagged‐dependent variable suffices). Our examples tentatively suggest that specifying the exact underlying cross‐ and auto‐lag structure becomes particularly important in situations that exhibit complex temporal dynamics (as in the density‐dependent example; Box 1), which provides a direction for future work on this topic.

### 5.3 Identifying the cross‐lag structure in dynamical models

In empirical studies, the causal pathways that generated the data are not known, meaning that deciding on the appropriate cross‐ (and auto‐) lag structure—and its functional form—is far from straightforward. Three types of approaches may provide some guidance in determining an appropriate model structure. First, in line with the SEM philosophy of constructing theoretically justified models representing the starting point for model selection, one can make assumptions about the underlying pathways based on theory or a priori information acquired through experiments. For instance, in the trade‐off example, modelling a causal cross‐lag pathway due to a cost of reproduction may be supported by brood size (reproductive effort) manipulations that illustrate growth costs. Furthermore, most researchers likely already have a reasonable understanding of how group or population size depends on vital rates based on previous research and population dynamical theory. However, our knowledge on all contributing processes may be incomplete, for example in our simulation examples we assumed that population and group size were determined by reproduction and survival only, while dispersal may also play a role and this is much harder to study in the field. If the number of immigrants and emigrants cannot be measured, it may be hard to model the specific cross‐ and auto‐lag structure correctly. Yet, if dispersal is high, modelling a cross‐lag may not be needed in the first place as population or group size will only weakly depend on local reproduction or survival.

Second, one could also explore evidence for cross‐lag patterns in the data itself. In the trade‐off example, it may not be obvious a priori whether a cross‐lag needs to be modelled, as this depends on the likelihood that a cost of reproduction exists, which is notoriously difficult to determine without proper experiments (Reznick et al., 2000). In such situations, a first step could be to determine whether *X_t_* and *Y_t_*_−1_ are correlated in the dataset at hand. However, the presence of such a correlation is a necessary, but not a sufficient condition. Fieberg and Ditmer (2012) showed that ignoring measurement error in *X* or ignoring important confounding variables can also cause *X_t_* and *Y_t_*_−1_ to be correlated in the absence of a causal cross‐lag. In what situations this may occur remains an open question, as none of our simulated examples that ignored measurement error in either *X* or *Y* in the absence of a causal cross‐lag generated a correlation between *X_t_* and *Y_t_*_−1_ (see Supplementary Material E). Simulation studies that mimic effects of confounding variables or measurement error could be used to further explore the likelihood of a correlation between *X_t_* and *Y_t_*_−1_ not being due to a causal cross‐lag.

Third, various more formal techniques than the above‐described exploratory approach exist to investigate whether there is evidence for a causal cross‐lag in the dataset at hand (reviewed by Hannisdal & Liow, 2018). The d‐separation test of graph theory can help to identify if there is evidence for a causal cross‐lag after conditioning on confounding variables (Shipley, 2016). Linear stochastic differential equations are a tool to both identify causal pathways and estimate parameters in longer time series (Hunt, 2006). Transfer entropy (Schreiber, 2000) and convergent cross‐mapping (Sugihara et al., 2012) are more model‐free approaches to investigate causality of pathways, the latter being particularly useful for multivariate time series that exhibit nonlinear dynamics.

Multiple of the above three approaches could be combined to support the choice of statistical model structure. Goodness of fit statistics of the chosen model can be checked (e.g. Grace, 2006) while tools for model comparison of multivariate models with competing cross‐ and auto‐lag structures are available (Vehtari et al., 2017).

## 6 ADDITIONAL INTERACTING SOURCES OF BIAS: MEASUREMENT ERROR

Thus far, we assumed that variables are measured with little or no error, but in practice this assumption is often not met. Measurement error in *X* or *Y* can also cause estimation bias. For example, measurement error may cause upward bias in auto‐lagged data, which has received much attention in the context of (over)estimating the strength of density dependence of population size/growth (e.g. Freckleton et al., 2006; Lebreton & Gimenez, 2013). However, little is known about estimation bias due to ignoring measurement error in cross‐lagged data structures. In Box 3, we show, for our three cross‐lagged simulated data examples, that (a) the direction and extent of bias due to measurement error can depend on the cross‐lag structure, and (b) that the direction of bias due to ignoring measurement error can be in a direction opposite to any bias caused by ignoring cross‐lags (e.g. in the density‐dependent example ignoring measurement error in *Y* leads to underestimation while ignoring cross‐lags is expected to cause overestimation of the strength of negative density dependence). Thus, for studies that have ignored both covariate endogeneity and measurement error, the overall direction of bias can be hard to predict. Reassuringly, dynamical structural equation models are in principle flexible enough (using latent variables; Tutorial 2) to account for measurement error too when analysing multiple time series (shown in Box 3 for all three simulation examples), although they are likely more data hungry.

BOX 3 **Bias due to ignoring measurement error and how to account for this in SEM**

In practice, variables are rarely measured with little or no error. It is well known that ignoring measurement error can cause bias, for example, when analysing auto‐lagged data (Freckleton et al., 2006). Furthermore, Fieberg and Ditmer (2012) showed that measurement error in the predictor variable (*X*) can also influence inference from cross‐lagged data in their example. In this Box, we first explore how ignoring measurement error in *X* or *Y* in our three simulation examples may cause bias in estimating the contemporaneous effects of interest *b* and next show that DYN_SEM models are flexible in accounting for such measurement error.

For simple situations with uncorrelated measurement errors among variables, theory predicts that measurement error in a predictor variable will bias estimates of *b* (the contemporaneous effect of *X_t_* on *Y_t_*) towards zero due to regression dilution while measurement error in response variable *Y* will not affect the estimation of *b* (but only affect the correlation coefficient or *R* ^2^; Grace, 2006). However, for more complex situations, like some of our cross‐lagged multivariate examples, the effects of measurement error in *X* and *Y* are likely different and harder to predict a priori (Fieberg & Ditmer, 2012; Grace, 2006).

For the analysis on potential estimation bias due to measurement uncertainty, we added measurement error to the previously described simulated data (based on Equations 2–4 in Box 1). The values of measurement error variance were equal to 25% of the total variance in, respectively, *X* and *Y*, which amounts to a fairly high reliability (average correlation between measurements of 0.75). We simulated datasets with varying values of *b* based on 100 subjects followed for 10 time steps each. The DYN_SEM+ model that was used to account for measurement errors, extended on the DYN_SEM models from Figure Box 2d by the inclusion of latent variables that describe the measurement process (Figure Box 3‐1). In the DYN_SEM+ model, the amount of measurement error was assumed to be known from external sources, such as repeated measurements. In Tutorial 2, we provide R code used to perform the simulations and analysis (Brouwer & van de Pol, 2021).

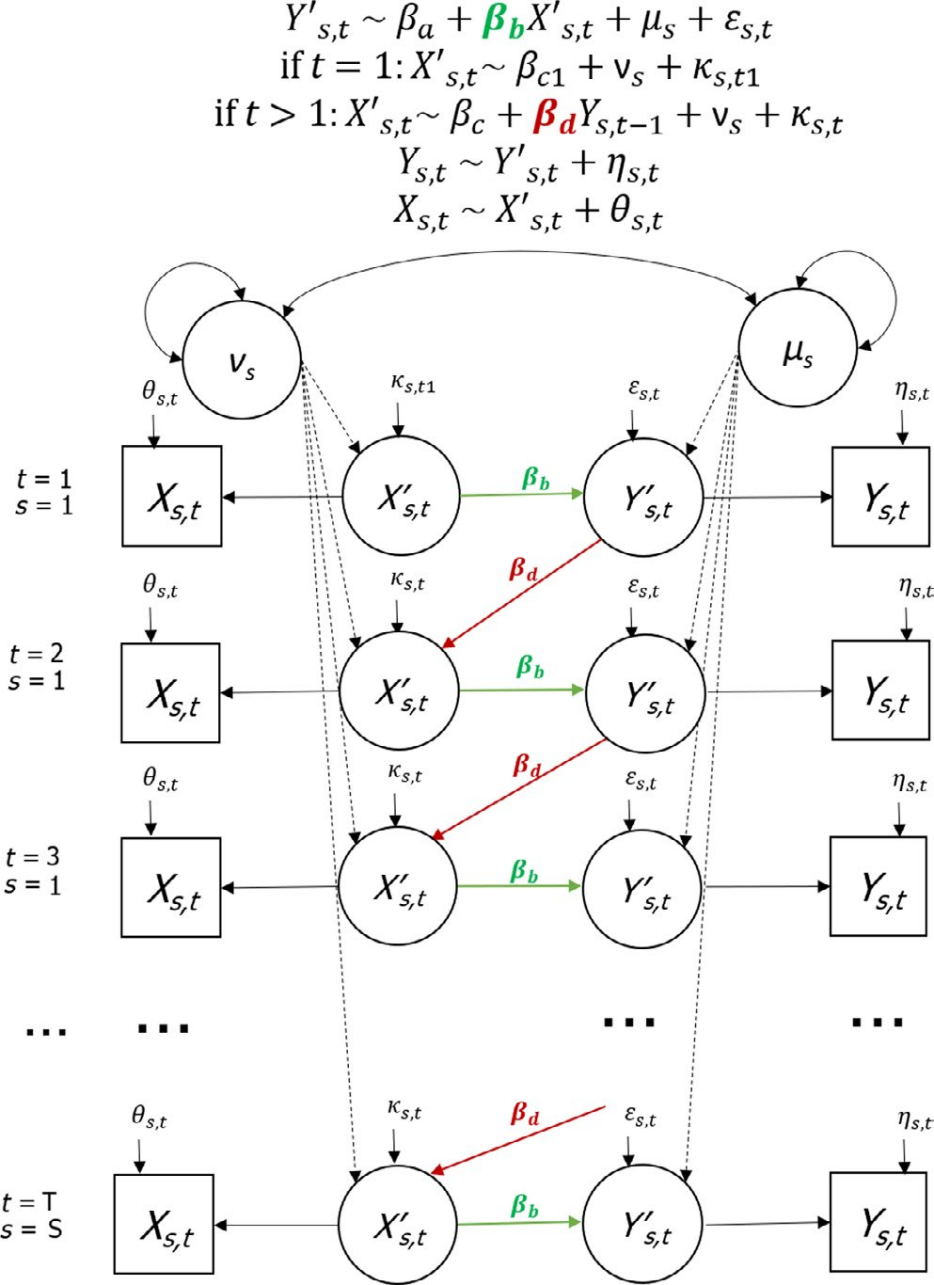

FIGURE BOX 3‐1: Structure of the structural equation model that accounts for measurement error in *X* and *Y* (DYN_SEM+) for a situation of a trade‐off with time series of multiple subjects. For DYN_SEM+ models, the group living and density‐dependent example, see Supplementary Material F. Latent variables *X*' and *Y*' are presented by circles as they are not directly observed, and observed variables *X* and *Y* have error terms ($\theta_{s,t}$ and $\eta_{s,t}$) that reflect the measurement error

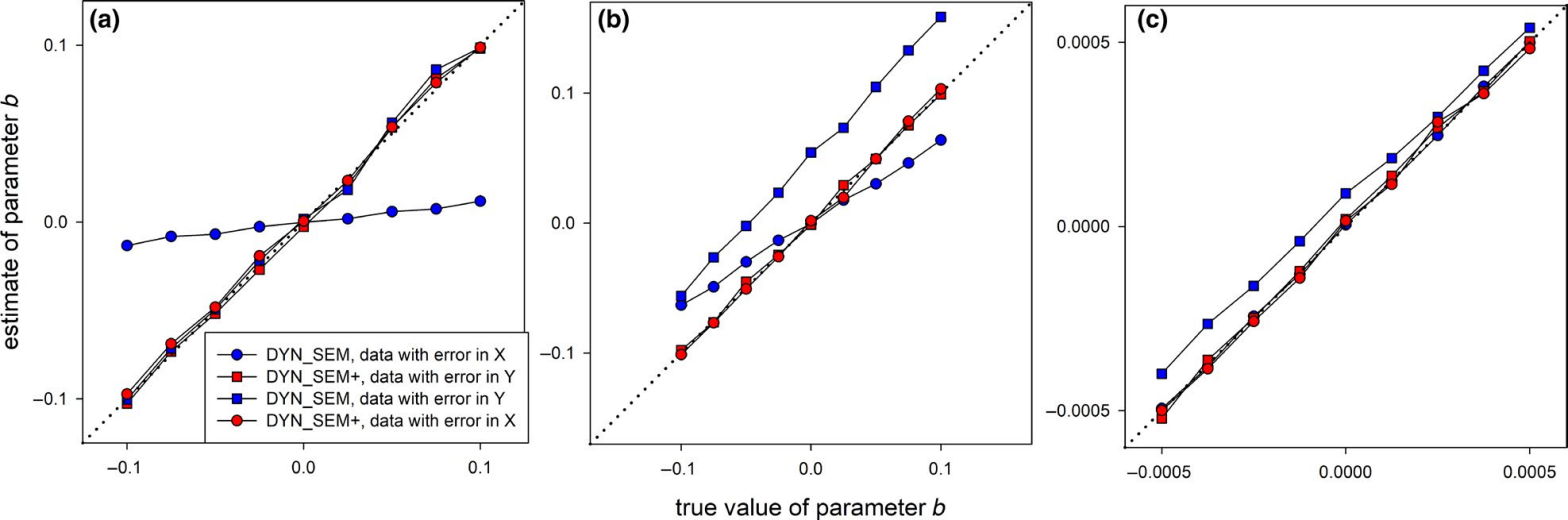

FIGURE BOX 3‐2: Bias in the estimate of parameter of interest *b* due to measurement error in *X* or *Y* for different true values of *b*, when using either a structural equation model that does not (DYN_SEM) or does model the measurement error process (DYN_SEM+), for the (a) trade‐off, (b) group living and (c) density‐dependent example simulated datasets

Analyses of the simulated example datasets that contained measurement error confirmed that in the simplest cross‐lag situation, reflecting that of a trade‐off, only measurement error in *X* biased estimates of *b* towards zero (Figure Box 3‐2a), consistent with the expectation due to regression dilution. In the more complex situation of the group living example, both measurement error in *X* and *Y* caused bias (towards zero and upwards, respectively, Figure Box 3‐2b), while in the situation of density dependence only measurement error in *Y* caused bias (upwards, Figure Box 3‐2c). These results thus suggest that (a) the direction and extent of bias due to measurement error can depend on the cross‐lag structure and (b) that direction of bias due to ignoring measurement error can be in a direction opposite to any bias caused by ignoring cross‐lags (e.g. in the density‐dependent example, we see that ignoring measurement error in *Y* leads to an underestimation of the strength of negative density dependence (Figure Box 3‐2c) while ignoring cross‐lags is expected to cause overestimation of the strength of density dependence; Figure 2b‐i). Thus, for studies that have ignored both covariate endogeneity and measurement error, the direction of bias that results from the combined action of both these sources of bias can be hard to predict.

Reassuringly, in all above situations, a dynamical error‐in‐variable model that specifically models the measurement error process using latent variables (DYN_SEM+) produced unbiased estimates of *b* in the presence of measurement error in *X* or *Y* (Figure Box 3‐2). Thus, in principle, dynamical structural equation models are flexible enough to also deal with additional bias due to measurement error when analysing multiple time series, but they are likely to be even more data hungry (above simulations were based on relatively large sample size: *n* = 1,000)

## 7 IT CAN MATTER IN REAL LIFE AS WELL: A CASE STUDY

Our graphical model explained why bias is expected to occur and our simulated examples highlighted that such biases can potentially be substantial and even occur in quite long time series when using static regression models on cross‐lagged data. Could the use of different estimation methods also affect key biological conclusions in a real‐world case study? To answer this question, we looked at cooperatively breeding red‐winged fairy wrens *Malurus elegans*, which mirrors our previous simulation example on group living in that we are interested in estimating the effect of group size on a group's annual offspring productivity from time series on multiple subjects (groups). In Box 4, we explain the details of the study system, data collection and how we identify the presence and type of cross‐lag in this dataset while Tutorial 3 provides the data and R code to reproduce the analysis and figures (Brouwer & van de Pol, 2021).

BOX 4 **Cross‐lags in reality—Benefits of group living in fairy‐wrens**

In our real‐world case study, we are interested in estimating the effect of group size on a group's annual offspring productivity from time series on multiple bird‐groups, structurally similar to the simulation example on group living. We aimed to quantify the within‐group association between group size and offspring production, as we expect that cross‐sectional patterns are confounded by among‐group heterogeneity in territory quality (Brown, 1978). Tutorial 3 provides the dataset and shows how to analyse it using R (Brouwer & van de Pol, 2021).

As part of a long‐term study, longitudinal data on group size, group productivity and survival were collected on 108 different groups of the cooperatively breeding *Malurus elegans* over 9 years (2008–2016; 678 group‐years; Brouwer et al., 2020). The study area comprised ~75 territories in which >99% of these red‐winged fairy‐wrens were individually recognizable by colour bands. In this area, each territory was checked at least fortnightly for group composition, survival and breeding activity throughout the breeding season. In addition, the surrounding areas were checked for the rare disperser, which in combination with *M. elegans*' extreme levels of male and female philopatry, and the isolated nature of the population (Brouwer et al., 2014) ensures that survival can reliably be inferred from presence/absence of individuals in a given year (annual detection rate is >99% in the main study area; Lejeune et al., 2016). We defined annual group productivity as the number of offspring produced that survived until the beginning of the next breeding season, group size as the number of adult group members (a breeder pair with 0–8 subordinates, typically previous‐year offspring), and survival as whether or not an adult group member survived from one breeding season to the next.

We inferred the presence and type of cross‐lag from external knowledge on the system. As offspring from the previous year almost always remain in their natal group (Brouwer et al., 2014), a positive within‐group cross‐lag between group size *X_t_* and reproductive success in the previous year *Y_t_*_−1_ is expected. Furthermore, because dispersal among groups is limited, the only other main contributor to group‐size variation is the survival of adult group members. We thus have good a priori reasons to assume that the underlying dynamics in our real‐world study reflects the temporal dynamics of the theoretical example on group living (Equation 3, Box 1), and hence modelled the cross‐ and auto‐lag structure to reflect this specific structure in the DYN_SEM (Figure Box 2d‐ii). Nevertheless, we empirically confirmed that a strong positive within‐group cross‐correlation between group size and previous‐year productivity was present in the *M*. *elegans* data (Figure Box 4a).

Furthermore, previous studies have shown that *M. elegans* territories differ systematically in their reproductive and survival rates (some groups always outperform others in various aspects; Lejeune et al., 2016). And indeed, we found a positive correlation between a group's average reproduction and survival (*r* = 0.53), which also likely caused the strong positive correlation between a group's average reproduction and group size (*r* = 0.64). Therefore, to avoid a confounding of the estimated effect of group size on productivity with among‐group associations due to, for example, territory quality, we focussed estimation on the within‐subject effect of group size (*X_t_*) on a group's offspring productivity (*Y_t_*) using the STAT_WITHIN model of Figure Box 2b and the DYN_SEM of Figure Box 2d‐ii. The STAT_WITHIN model estimates how productivity changes with group size within groups studied over multiple years by means of within‐group centring while the DYN_SEM estimates the group‐size effect while accounting for any among‐group associations by including an among‐subject covariance term. We found that a Poisson distribution approximated group productivity and size well (see Tutorial 3) and a assumed a binomial distribution for survival. These discrete response variable models were implemented using the Bayesian package rstan (Guo et al., 2016), using weakly informative priors that make minimal assumptions about the model (see Tutorial 3 for details).

The STAT_WITHIN model that ignored any cross‐lag estimated there to be a negative effect of group size on productivity of −6% offspring per additional group member (95% credible intervals overlapped with zero [−15%, +4%], analysing the time series of each group separately and averaging their static regression coefficient gave identical results). By contrast, DYN_SEM suggested a strong positive association of +12% offspring per additional group member (95% credible intervals did not overlap with zero [+2%, +22%]; Figure Box 4b). This difference in estimated effect size of +12% versus −6% is biologically very meaningful as in the former case it implies that the largest groups (10 members) had double the productivity than the smallest groups (two members), while in the latter it implies that the largest groups had nearly half the productivity of the smallest groups (Figure Box 4c).

This real‐world example shows that the biological interpretation can completely depend on the chosen estimation method. Assuming that our understanding of the causal temporal dependencies between group size, reproduction and survival is reliable in this model system, and based on our previous graphical and simulation results we interpret this outcome as that the conventional static model underestimates the true effect size, as positive cross‐lag causes downward bias (Figure 2b‐ii). The static model thereby likely obfuscated evidence in the data for large benefits of cooperation, as suggested by the strongly positive DYN_SEM estimate of group size on productivity (that likely was unbiased given the >100 *M*. *elegans'* groups followed for >5 years, Figure 2d‐ii). Finally, bias due to measurement error is likely negligible in this intensively studied population, which could otherwise cause upward bias and further complicate interpretation of results (Figure Box 3‐2b).

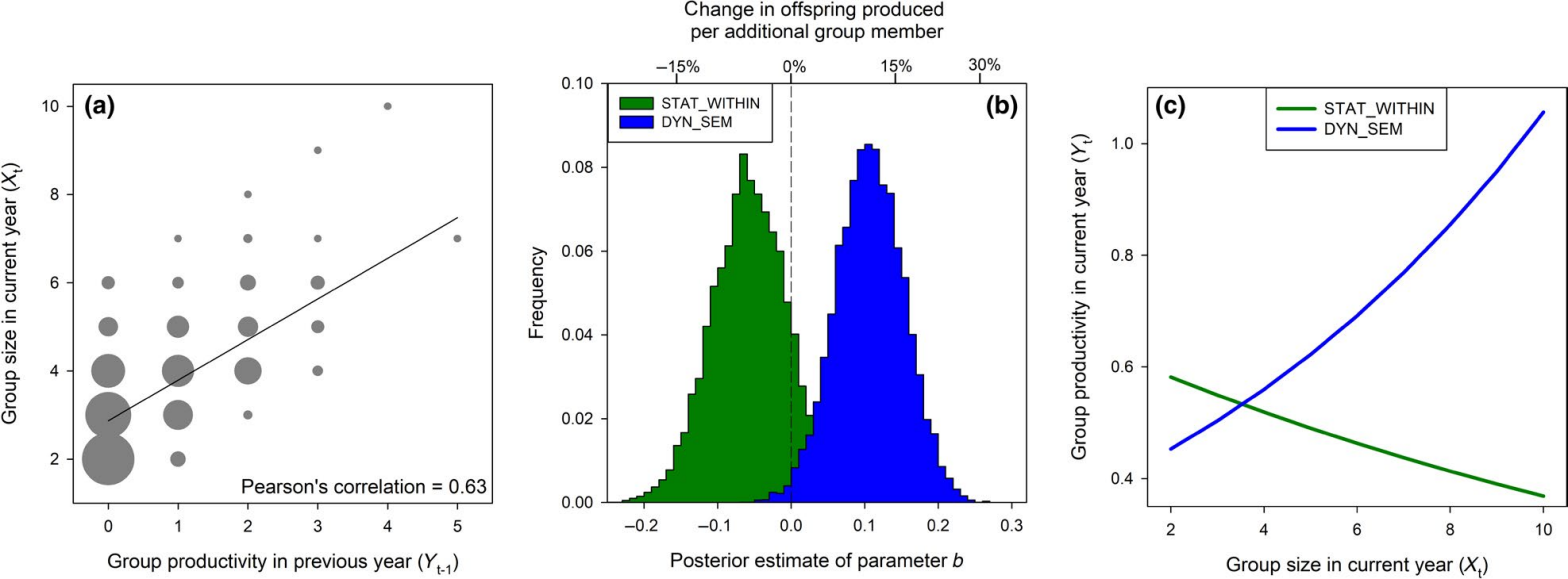

FIGURE BOX 4: (a) The (within‐group) cross‐lag correlation present in the *Malurus elegans* dataset, (b) histogram of the posterior estimate of the effect of group size (*X_t_*) on group productivity (*Y_t_*; number of offspring) from a static and dynamical model, and (c) their predicted group‐size effect across the range of group sizes observed in this population. In (a), symbol size is proportional to sample size (*n* = 678 in total). Note that in (b) the parameter estimate of *b* is on the log‐scale (Poisson regression) and biologically relevance of effect size is plotted on the second *x*‐axis at the top (e.g. a value of +12% implies that for each additional group member the number of offspring produced by the group increases with 12% compared to the productivity in a typically sized group).

The main result is that the static model that ignored the cross‐lag estimated there to be a negative effect of group size on productivity (6% less offspring per additional group member) while the dynamical model that specified a cross‐lag suggested a large positive effect of group size on productivity (12% more offspring per additional group member; Figure Box 4b). This real‐world example shows that the biological interpretation can completely depend on the chosen estimation method: the static model suggests substantial costs (i.e. the largest groups producing about half that of the smallest groups) while the dynamical model suggests strong benefits of group living (i.e. the largest groups producing double that of the smallest groups).

Which model results should we now trust? Assuming that our understanding of the causal temporal dependencies between group size, reproduction and survival is reliable in this model system, and based on our previous graphical and simulation results we interpret our results as follows: First, the conventional static model likely underestimated the true effect size because positive cross‐lag is expected to cause downward bias in static models applied to short time series (Figure 2b‐ii). Second, we can be more confident that the dynamical model estimate is accurate, as our simulations showed that bias is unlikely for this type of cross‐lag, number of subjects and time‐series length collected in the case study (Figure 2d‐ii; >100 bird‐groups [subjects] followed for >5 years/time steps). A tentative conclusion could thus be that the static model likely obfuscated evidence in the data for large benefits of cooperation.

## 8 ALTERNATIVE DYNAMIC REGRESSION MODELLING FRAMEWORKS

We showed that dynamical structural equation models that specifically model the underlying cross‐lagged nature of the data‐generating process provide a useful tool to analyse cross‐lagged data, but only when multiple time series are available. Furthermore, SEMs can deal with the separate but additional problem of biased estimation due to measurement error (Grace, 2006; Shipley, 2016). Our goal here was to illustrate that SEM, as one of the more familiar type of multivariate models to ecologists, can be flexibly applied to the biological examples we discuss. However, depending on the specific question, dataset and modelling background, alternative multivariate time‐series frameworks may prove useful. Vector autoregressive models (and closely related cross‐lagged panel models that are widely used in the social sciences) can sometimes be seen as a specific type of SEM (du Toit & Browne, 2007; Mund & Nestler, 2019), though differences in algorithms and approaches do exist (e.g. SEM mechanistically builds upon causal structures while vector autoregressive and cross‐lagged panel models are based on a more theory‐free modelling philosophy). Furthermore, most multivariate frameworks allow for hierarchical data structures as well as for separating the structural and measurement process: latent variables may reflect that some structural variables may not be known, for example due to measurement error (see particularly state‐space models; de Valpine, 2002; Holmes et al., 2012). A major difference however is that vector autoregressive and cross‐lagged panel models focus on the cross‐lags (e.g. effect of *Y_t_*_−1_ on *X_t_*) and auto‐lags (e.g. effect of *Y_t_*_−1_ on *Y_t_*) as being the parameters of interest. Notably, such models typically do not explicitly include any contemporaneous effects that are of interest in our biological examples (the effect of *X_t_* on *Y_t_*; though correlated error terms of *X_t_* on *Y_t_* are often included).

A practical challenge of dynamical models is that it is more difficult to generalize them. The lagged‐dependent variable model cannot directly be applied to discrete data (e.g. Poisson or Bernoulli), and while state‐space models may provide an alternative, they are data hungry (de Valpine, 2002). Most frequentist structural equation modelling software also has limited procedures to deal with non‐Gaussian data and can only handle simple random effects structures (but see Muthén & Muthén, 2015; Rosseel, 2012). Fortunately, Bayesian statistical inference with Markov chain Monte Carlo sampling offers a flexible alternative (Monnahan et al., 2017), as illustrated by our real‐world case study that included both count and binomial data and multiple random effects (Tutorial 3).

## 9 DISCUSSION

Nature is complex. This seems like an obvious statement, but too often we reduce it to straightforward models. *Y* ~ *X* and that sort of thing. Not that there's anything wrong with that: sometimes *Y* is actually directly a function of *X* and anything else would be […] statistical machismo. But I would wager that, more often than not […] *Y* may be affected by a host of direct and indirect factors, which themselves affect one another directly and indirectly. If only there was some way to translate this network of interacting factors into a statistical framework to better and more realistically understand nature. Oh wait, structural equation modelling.—J. Lefcheck (2014)

Our study shows that the temporal dependencies often present in biological data are a situation for which the above statement is particularly appropriate. Modelling *Y* as a simple function of *X* generates asymptotically unbiased estimates in situations of cross‐lag, but this provides little practical relief: for most sample sizes that are realistically achievable in observational studies in the wild it generates systematic bias, even in the absence of measurement error. By ignoring cross‐lags, static regression models omit an important confounding variable, and thereby assume the covariate *X* to be exogenous with respect to the response variable *Y*, while it is in fact endogenous (Diggle et al., 2002). Cross‐lags between *Y* and *X*—if unaccounted for—ultimately cause temporal autocorrelation in the residuals of *Y*, which violates the assumption of independence in static regression models.

Although problems of ignoring covariate endogeneity have long been recognized in the statistical literature (Diggle et al., 2002), only few ecological studies have highlighted the challenges of analysing cross‐lagged data (Eisenhauer et al., 2015; Fieberg & Ditmer, 2012; Hefley et al., 2016; Ives et al., 2003). However, neither these ecological nor statistical studies focused on estimation bias in short time series. We also showed that the challenge of cross‐lags extends to a variety of biological problems (it is also related, but should not be confused with similar challenges and asymptotic biases when analysing autoregressive data, such as in studies of density‐dependent changes in population size; St. Amant, 1970; Maelzer, 1970). Specifically, we found that estimation bias can be substantial when analysing a single subject time series of a length that is realistically achievable in ecological and evolutionary studies (e.g. a population followed for 10–20 years/time steps). Additionally, we identified that the common practice of focusing analyses on within‐subject patterns to avoid ecological fallacies, means that even studies that analyse time series of multiple subjects (individuals, groups and populations) are exposed to similar challenges as studies analysing single time series.

This thus far unrecognized bias in short time series potentially may have important implications. Since the mechanisms causing cross‐lags—such as feedback loops, two‐way causality and sequential allocation choices of limited resources—are common in biology, the implications could be relevant for many fields. Furthermore, the common practice of using static approaches and longitudinal data implies that results reported in the ecological and evolutionary literature are likely to be biased, with the direction of bias depending on the sign of the cross‐lag. For example, we could expect systematic underestimation of the existence and strength of life‐history trade‐offs and benefits of group living, and systematic overestimation of the strength of density dependence of vital rates in the existing literature utilizing time‐series data. The extent of these biases in published studies, and whether it is severe enough to really affect our biological conclusions, remains to be determined, not in the least because other biases may exist that act in opposite directions or directly interact with the bias due to cross‐lag (e.g. due to the common practice of ignoring measurement error).

Notwithstanding, our real‐world case study illustrates that choosing either static or dynamical statistical models can completely alter the biological interpretation of studies, in this case the evidence for benefits of group living. Re‐analyses of a large set of studies on the benefits of group living using both static and dynamical regression models could shed further light on how large biases are likely to be in the literature. However, such re‐analysis currently appears infeasible for studies on density dependence of vital rates, as they typically deal with a single time series, for which dynamical and static models both produce bias. Possibly analytical or bootstrap bias correction may provide a post‐hoc solution. Alternatively, the use of Bayesian informative priors—based on information from published studies—may improve parameter identifiability (Hobbs & Hooten, 2015) in the density‐dependent case specifically, and in complex dynamical models in general.

A clear disadvantage of multivariate models is that they may require additional data or assumptions in situations of complex cross‐lag structure. In our multiple time‐series example of reproductive benefits of group living, only the SEM that included data on the survival vital rate consistently provided unbiased estimates. Furthermore, particularly in cases of complex cross‐lag structure misspecification of the dynamical process in the regression model appears to be a risk, as in practice it will be impossible to be completely sure that all relevant pathways are included (e.g. many vital rates can affect population dynamics: immigration, emigration, recruitment, reproduction, survival, or there could be higher order cross‐lags or autoregressive signals in the noise, or nonlinearity in the functional form of cross‐lags). Applying dynamical multivariate models to cross‐lagged data thus requires critical thinking about which underlying causal pathways might be relevant, and sometimes data exploration to study whether the patterns in the data are consistent with such causal dependencies (Hannisdal & Liow, 2018; Shipley, 2016). Furthermore, it is important to be aware (and explore) how sensitive results can be to the chosen model specification. This should really not be viewed as a trivial challenge: there are usually several plausible causal networks that require consideration, and these causal networks may involve unmeasured variables of which the causality may be difficult to differentiate from the causal pathways of interest with sparse datasets (even if many subjects are studied).

### 9.1 Conclusions

In conclusion, we argue that biologists should be more alert for cross‐lags in observational longitudinal data and the consequences that this has for parameter estimation. In some situations, thoughtful use of dynamical models provides a better alternative to the widely used static models. However, more research is needed to understand in which situations this is particularly relevant, what model complexity is optimal given the structure and amount of data available, and whether other aspects than bias may also be important to consider (precision, prediction error and statistical power). In many ways, we have likely only scratched the surface on the challenges imposed by cross‐lags, as the impact of cross‐lags on contemporaneous effects in particularly short time series has thus far not received any attention among statisticians as far as we are aware, and thus there is no theory to rely on. We also acknowledge that the dynamical multivariate models presented will be technically more challenging to apply than static univariate models, but hope that our study convinces readers that this is not statistical machismo and instead can be crucial for a proper understanding of key biological questions. Sometimes, simple questions and datasets just can be difficult to analyse, but we hope that our R‐tutorials for the simulated and empirical examples provide useful tools to make this task somewhat easier.

## CONFLICT OF INTEREST

Both authors declare that they have no conflict of interest.

## AUTHORS' CONTRIBUTIONS

Both authors developed the ideas and designed the study; M.v.d.P. performed the analysis; L.B. runs the empirical study; M.v.d.P. and L.B. wrote the paper together.

## Supporting information

Supplementary Material

## Data Availability

Data, Tutorials and R code available from the Dryad Digital Repository https://doi.org/10.5061/dryad.7h44j0ztw (Brouwer & van de Pol, 2021).
