# Supplementary material for: Cross‐lags and the unbiased estimation of life‐history and demographic parameters
Source: J Anim Ecol. 2021 Aug 18;90(10):2234–53. doi: 10.1111/1365-2656.13572 (PMC9290935; doi:10.1111/1365-2656.13572)
Supplement: Supplementary file 1 — Supplementary Material [file JANE-90-2234-s001.zip › jane13572-sup-0005-Supinfo.docx]

**Supplementary material A:**

**How variance in X and Y changes with time series length in cross-lagged data**

In supplementary material A we explain how the variance in X and Y changes as a function of time series length for the three biological examples of cross-lagged data described in the main text: (a) density dependence of vital rates (b) benefits of group living, and (c) a trade-off. We generated a single time series dataset and varied the time series length between 3 and 80 timesteps and also simulated one very long time series with 200 timesteps to assess its asymptotic convergence. This stochastic procedure was replicated 10,000 times and for each time series duration we subsequently quantified the variance in X and Y across the time series, and then average that across all replicates with the same duration (giving VarX_d_). As the variance increases asymptotically with increasing time series length, we estimated the asymptotic variance in X and Y as the variance in X and Y at duration 200. We next expressed the variance in X and Y as a proportion of the asymptotic variance using: propVarX_t_ = VarX_t_ / VarX_t=200_

Figure A show that both the variance in X and Y initially increases strongly with time series duration and only levels off at length of time series that are not always easily attained in longitudinal observational studies in the wild. For comparison we have plotted the variance time plots next to the estimate of parameter of interest b for a single time series, which shows that the estimation bias by static methods shows a similar dependency on time series length as the variance (left panels; copied from Fig. 2 in main text).

*
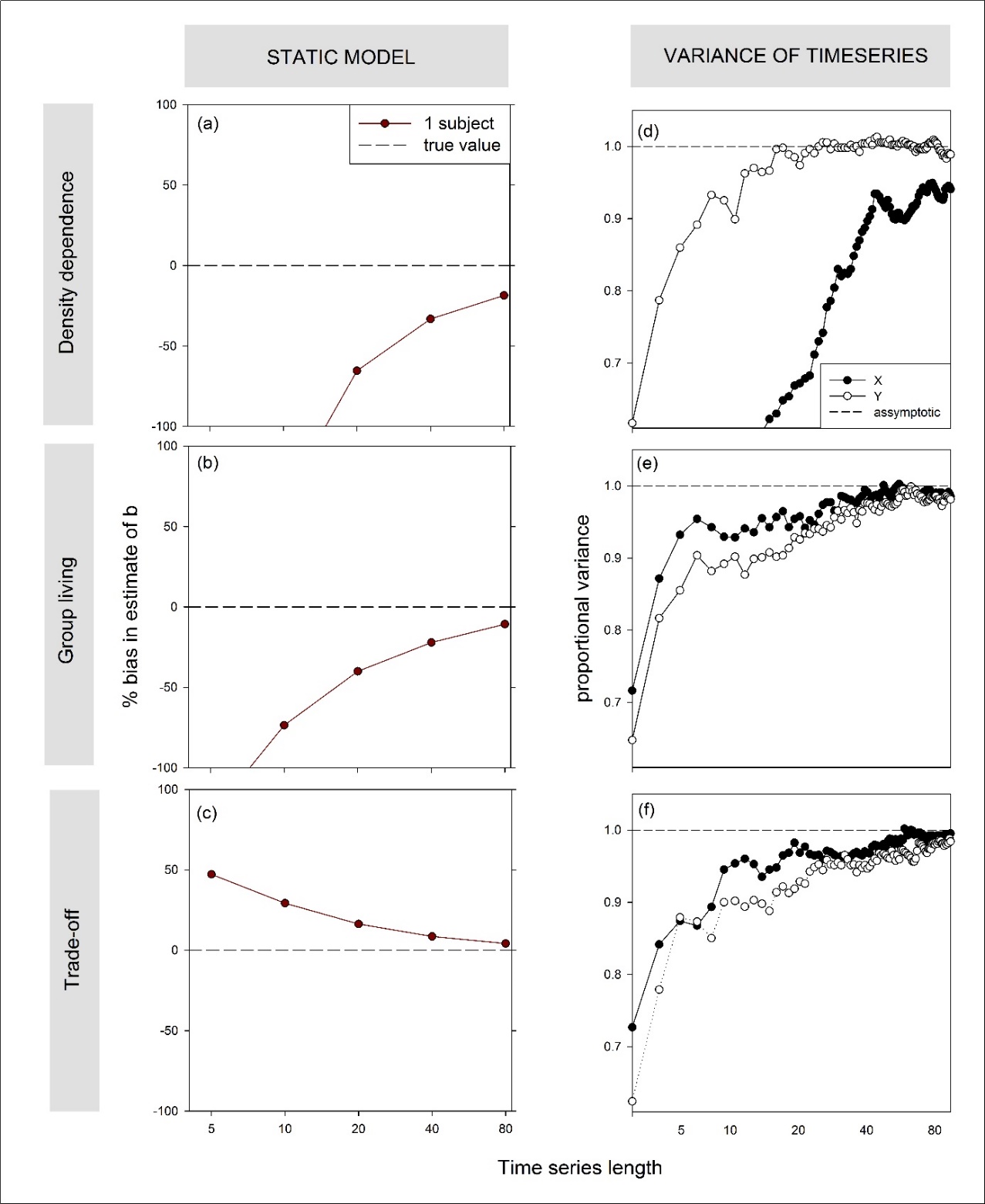
*

**Figure A**: The (bias in) estimates of parameter b (effect of X_t_ on Y_t_) as a function of time series length determined by static (STAT WITHIN or STAT_OVERALL which are equivalent when analysing one subject) applied to simulated cross-lagged data (see legend). Panels reflect a situation of (a) the density dependence of vital rates (b) the benefits of group living, and (c) a trade-off. Panels (d)-(f) show how the variance in X and Y (averaged across 10,000 replicate time series) increases and asymptotically levels of with increasing time series length in the three examples. Note the logarithmic x-axis.

**Supplementary material B:**

**Literature search on whether ecological studies consider cross-lags and the statistical methods they use**

In this supplementary material B we describe the literature search performed to determine the approaches studies have used to analyse (1) density dependence in vital rates and (2) the benefits of group living. We first performed a literature search in Google Scholar (<https://scholar.google.nl/>, accessed 15-04-2020) to identify empirical studies on both topics. For studies on density dependence we used the following search term (generating 3100 hits):

("density dependent survival" OR "density dependent reproduction") AND (longitudinal OR "long-term")

Note that we used the terms “longitudinal” OR "long-term" and not the term “time series” as few studies appear to use the more statistical term “time series” in their papers.

For studies on group living we used the following search term (generating 3300 hits):

("group size") AND (reproduc* OR surviv*) AND (“benefits”)

We then first checked if any of these studies mentioned the terms “covariate endogeneity” or “cross-lag” by expanding the above search terms with:

AND ("covariate endogeneity" OR "cross-lag")

In both the density dependence and group living search there were no hits, suggesting none of the studies on this topic have linked these analyses to the statistical literature on the problem at hand. We note that adding the term “two-way causality” (i.e. AND ("covariate endogeneity" OR "cross-lag" OR “two-way causality”) also did not results in identifying any papers that discussed cross-lags. Adding the term “feedback” (i.e. AND ("covariate endogeneity" OR "cross-lag" OR “feedback”) did result in hundreds of results, but a quick scan of 20 of such papers taught us that the term “feedback” was used to describe the feedback due to density dependence on population growth rate (an implication of density dependence in vital rates), the term did not refer to a cross-lag in any of these 20 papers.

Next, to verify that these studies indeed generally ignored cross-lags and identify the type of regression models they used, we did a smaller more in-depth literature investigation by selecting and reading recent papers on this topic and recorded whether they used a static or dynamic regression model to analyse time series. We limited our search set to papers published since 2013 (as recent papers are most likely to reflect the currently state-of-the-art analytical approaches), reducing numbers to respectively 406 and 959 hits. Subsequently, in order of appearance (reflecting their relevance and number of citations as determined by the search algorithm; <https://scholar.google.com/scholar/about.html>), we downloaded and started reading each hit. We first read the title and abstract and publication medium to determine if it met the following criteria:

1. It was a publication in a peer-reviewed scientific journal
2. It was an empirical paper
3. It utilized longitudinal (timeseries) data (and not primarily spatial heterogeneity)
4. It either analysed the effect of population density or group size on reproduction or survival

If it seemed likely that the hit met these selection criteria, we further checked this by downloading the PDF, reading the remainder of the paper and recorded (i) the identity of the hit (ii) if the paper did or did not mention that cross-lags, covariate endogeneity (or any other terminology implying the same temporal dependency, but we found no other terms) may have interfered with estimation, (iii) if the paper used static or dynamics regression models that accounted for any possible cross-lag, (iv) the average duration of the time series analysed, or if unavailable, the study duration.

For both the search on density dependence and group living studies, we stopped our literature search once 10 papers on either topic were deemed to have fulfilled our criteria, as it turned out all of these 10 papers gave the same outcome (i.e. none of the studies discussed cross-lags and all used static regression models; see Table B1), hence more reading would have unlikely to be much more informative.

| **Nr** | **Search** | **Reference** | **Discusses cross-lags?** | **Static or dynamic model?** | **Duration** |
| --- | --- | --- | --- | --- | --- |
| 1 | Density dependence | Gane & Burt, 2016 | No | Static | 18 years |
| 2 |  | Grünkorn et al. 2014 | No | Static | 22 years |
| 3 |  | Layton‐Matthews et al. 2019 | No | Static | 27 years |
| 4 |  | Paterson et al. 2018 | No | Static | 6 months |
| 5 |  | Poláková et al. 2018 | No | Static | 13 years |
| 6 |  | Marra et al. 2015 | No | Static | 14 years |
| 7 |  | Vincenzi et al. 2016 | No | Static | 10 years |
| 8 |  | Gamelon et al. 2016 | No | Static | 38 years |
| 9 |  | McDonald et al. 2016 | No | Static | 21 years |
| 10 |  | Norman & Peach 2013 | No | Static | 22 years |
| 11 | Group living | Groenendijk et al. 2015 | No | Static | 22 years |
| 12 |  | Paquet et al. 2015 | No | Static | 8 years |
| 13 |  | Marneweck et al. 2019 | No | Static | 23 years |
| 14 |  | Rézouki et al. 2016 | No | Static | 22 years |
| 15 |  | Creel & Creel 2015 | No | Static | 5 years |
| 16 |  | Mumme et al. 2015 | No | Static | 33 years |
| 17 |  | Keynan & Ridley 2016 | No | Static | 35 years |
| 18 |  | Koenig et al. 2015 | No | Static | 41 years |
| 19 |  | Wiley & Ridley 2018 | No | Static | 13 years |
| 20 |  | van Boheemen et al. 2019 | No | Static | 13 years |

*Table B1: Overview of papers looking at either density dependence in vital rates or the costs and benefits of group living and whether the discuss cross-lags (in any way, also using other terminology) and used static or dynamic models to analyse the problem. The duration of the study is also noted as an indicator of the upper limit of time series length in these studies.*

**References**

Creel, S., & Creel, N. M. (2015). Opposing effects of group size on reproduction and survival in African wild dogs. Behavioral Ecology, 26(5), 1414-1422.

Gamelon, M., Grøtan, V., Engen, S., Bjørkvoll, E., Visser, M. E., & Sæther, B. E. (2016). Density dependence in an age‐structured population of great tits: identifying the critical age classes. Ecology, 97(9), 2479-2490.

Gane, J., & Burt, A. (2016). Longevity and survival of the Endangered Seychelles Magpie Robin Copsychus sechellarum. Ostrich, 87(1), 81-83.

Groenendijk, J., Hajek, F., Schenck, C., Staib, E., Johnson, P. J., & MacDonald, D. W. (2015). Effects of territory size on the reproductive success and social system of the giant otter, south‐eastern Peru. Journal of Zoology, 296(3), 153-160.

Grünkorn, T., Potiek, A., Looft, V., Jonker, R. M., Chakarov, N., & Krüger, O. (2014). Territory quality affects the relative importance of habitat heterogeneity and interference competition in a long‐lived territorial songbird. Journal of avian biology, 45(1), 15-21.

Keynan, O., & Ridley, A. R. (2016). Component, group and demographic Allee effects in a cooperatively breeding bird species, the Arabian babbler (Turdoides squamiceps). Oecologia, 182(1), 153-161.

Koenig, W. D., & Walters, E. L. (2015). Temporal variability and cooperative breeding: testing the bet-hedging hypothesis in the acorn woodpecker. Proceedings of the Royal Society B: Biological Sciences, 282(1816), 20151742.

Layton‐Matthews, K., Loonen, M. J., Hansen, B. B., Coste, C. F., Sæther, B. E., & Grøtan, V. (2019). Density‐dependent population dynamics of a high Arctic capital breeder, the barnacle goose. Journal of Animal Ecology. https://doi.org/10.1111/1365-2656.13001

Marneweck, D.G., Druce, D.J. & Somers, M.J. (2019) Food, family and female age affect reproduction and pup survival of African wild dogs Behav Ecol Sociobiol 73: 65. https://doi.org/10.1007/s00265-019-2676-x

Marra, P. P., Studds, C. E., Wilson, S., Sillett, T. S., Sherry, T. W., & Holmes, R. T. (2015). Non-breeding season habitat quality mediates the strength of density-dependence for a migratory bird. Proceedings of the Royal Society B: Biological Sciences, 282(1811), 20150624.

McDonald, J. L., Bailey, T., Delahay, R. J., McDonald, R. A., Smith, G. C., & Hodgson, D. J. (2016). Demographic buffering and compensatory recruitment promotes the persistence of disease in a wildlife population. Ecology letters, 19(4), 443-449.

Mumme, R. L., Bowman, R., Pruett, M. S., & Fitzpatrick, J. W. (2015). Natal territory size, group size, and body mass affect lifetime fitness in the cooperatively breeding Florida Scrub-Jay. The Auk: Ornithological Advances, 132(3), 634-646.

Norman, D., & Peach, W. J. (2013). Density‐dependent survival and recruitment in a long‐distance Palaearctic migrant, the Sand Martin Riparia riparia. Ibis, 155(2), 284-296.

Paterson, J. E., & Blouin‐Demers, G. (2018). Density‐dependent habitat selection predicts fitness and abundance in a small lizard. Oikos, 127(3), 448-459.

Paquet, M., Doutrelant, C., Hatchwell, B. J., Spottiswoode, C. N., & Covas, R. (2015). Antagonistic effect of helpers on breeding male and female survival in a cooperatively breeding bird. Journal of Animal Ecology, 84(5), 1354-1362.

Poláková, K., Musil, P., Musilová, Z., & Zouhar, J. (2018). Density-dependent regulation of breeding success in the Red-crested Pochard Netta rufina. Bird study, 65(1), 92-97.

Rézouki, C., Tafani, M., Cohas, A., Loison, A., Gaillard, J. M., Allainé, D., & Bonenfant, C. (2016). Socially mediated effects of climate change decrease survival of hibernating Alpine marmots. Journal of Animal Ecology, 85(3), 761-773.

Van Boheemen, L. A., Hammers, M., Kingma, S. A., Richardson, D. S., Burke, T., Komdeur, J., & Dugdale, H. L. (2019). Compensatory and additive helper effects in the cooperatively breeding Seychelles warbler (Acrocephalus sechellensis). Ecology and evolution.

Vincenzi, S., Mangel, M., Jesensek, D., Garza, J. C., & Crivelli, A. J. (2016). Within‐and among‐population variation in vital rates and population dynamics in a variable environment. Ecological applications, 26(7), 2086-2102.

Wiley, E. M., & Ridley, A. R. (2018). The benefits of pair bond tenure in the cooperatively breeding pied babbler (Turdoides bicolor). Ecology and evolution, 8(14), 7178-7185.

**Supplementary material C:**

**Is the cross-lag crucial for the DYN_SEM in avoiding bias?**

If we compare the regression equation of DYN_SEM with those of the static models, taking the simplest example of the trade-off, we can see that the DYN_SEM differs from the static models in two key ways:

1. The DYN_SEM is multivariate and includes a cross-lag (Y_t-1_) in the equation for X_t_
2. The DYN_SEM includes a correlated random intercept with an among subject covariance between X and Y ($\sigma_{\mu,\nu}$).

This can be seen from the regression equations from Box 2 in the main text (Fig. Box2a, b & d-iii), which we repeat here:

**STAT_OVERALL:**  $Y_{s,t} \sim\beta_{a}+\beta_{b}X_{s,t}+\mu_{s}+\varepsilon_{s,t}$

with $\mu_{s}\sim Normal(0,$ ${\sigma_{\mu}}^{2}$), $\varepsilon_{s,t}\sim Normal(0,$ ${\sigma_{\varepsilon}}^{2}$)

**STAT_WITHIN:** $Y_{s,t} \sim\beta_{a}+\beta_{b}{(X}_{s,t}-\bar{X}_{s})+\mu_{s}+\varepsilon_{s,t}$

with $\mu_{s}\sim Normal(0,$ ${\sigma_{\mu}}^{2}$) $\varepsilon_{s,t}\sim Normal(0,$ ${\sigma_{\varepsilon}}^{2}$)

**DYN_SEM**: $Y_{s,t} \sim\beta_{a}+\beta_{b}X_{s,t}+\mu_{s}+\varepsilon_{s,t}$

$$if t=1:X_{s,t}\sim\beta_{c1}+\text{ν}_{s}+\kappa_{s,t1}$$

$$if t>1: X_{s,t} \sim\beta_{c}+\beta_{d}Y_{s,t-1}+\text{ν}_{s}+\kappa_{s,t}$$

with $\left( \begin{matrix} \mu_{s} \\ \text{ν}_{s} \end{matrix} \right)=MultivariateNormal(0,\left( \begin{matrix} {\sigma_{\mu}}^{2} & \sigma_{\mu,\nu} \\ \sigma_{\mu,\nu} & {\sigma_{\nu}}^{2} \end{matrix} \right))$

and $\varepsilon_{s,t}\sim N (0,$ ${\sigma_{\varepsilon}}^{2}$), $\kappa_{s,t}\sim N (0,$ ${\sigma_{\kappa}}^{2}$) , $\kappa_{s,t1}\sim N (0,$ ${\sigma_{\kappa1}}^{2}$)

In the main text we show that DYN_SEM is unbiased for situations of multiple time series, while the static models are not for shortish time series with among-subject covariance. We attribute this unbiasedness of the DYN_SEM to the inclusion of the cross-lag in the DYN_SEM together with the correlated random intercepts ($\mu_{s} and \text{ν}_{s})$ that allow the cross-lag in the regression equation of X_t_ to influence the estimation of the contemporaneous effect of interest in the regression equation of Y_t_.

Here we asses whether it is not solely the inclusion of the correlated random intercepts that is sufficient to remove any bias. We tested this by removing the cross-lag term for the DYN_SEM giving the following model:

**DYN_SEM_NO_CROSS_LAG**: $Y_{s,t} \sim\beta_{a}+\beta_{b}X_{s,t}+\mu_{s}+\varepsilon_{s,t}$

$$X_{s,t} \sim\beta_{c}+\text{ν}_{s}+\kappa_{s,t}$$

with $\left( \begin{matrix} \mu_{s} \\ \text{ν}_{s} \end{matrix} \right)=MultivariateNormal(0,\left( \begin{matrix} {\sigma_{\mu}}^{2} & \sigma_{\mu,\nu} \\ \sigma_{\mu,\nu} & {\sigma_{\nu}}^{2} \end{matrix} \right))$

and $\varepsilon_{s,t}\sim N (0,$ ${\sigma_{\varepsilon}}^{2}$), $\kappa_{s,t}\sim N (0,$ ${\sigma_{\kappa}}^{2}$)

In Fig. Suppl. C we can see that the bias of the DYN_SEM_NO_CROSS_LAG is similar as the bias as STAT_WITHIN, showing that the inclusion of the cross-lag is crucial for the unbiased estimation of the contemporaneous effect of interest b. We note that the inclusion of a correlated random intercept is also crucial for unbiased estimation, as if $\mu_{s} \& \text{ν}_{s}$ is uncorrelated (see DYN_SEM_UNCORRELATED below) then the equation for Y in the DYN_SEM and STAT_OVERALL is identical (as there are no shared model parameters between the regression equations of Y and X anymore), resulting in the same likelihood function and giving similar bias (Fig. Suppl. C).

**DYN_SEM_UNCORRELATED**: $Y_{s,t} \sim\beta_{a}+\beta_{b}X_{s,t}+\mu_{s}+\varepsilon_{s,t}$

$$if t=1:X_{s,t}\sim\beta_{c1}+\text{ν}_{s}+\kappa_{s,t1}$$

$$if t>1: X_{s,t} \sim\beta_{c}+\beta_{d}Y_{s,t-1}+\text{ν}_{s}+\kappa_{s,t}$$

with $\mu_{s}\sim Normal(0,$ ${\sigma_{\mu}}^{2}$), $\text{ν}_{s}\sim Normal(0,$ ${\sigma_{\text{ν}}}^{2}$)

and $\varepsilon_{s,t}\sim N (0,$ ${\sigma_{\varepsilon}}^{2}$), $\kappa_{s,t}\sim N (0,$ ${\sigma_{\kappa}}^{2}$), $\kappa_{s,t1}\sim N (0,$ ${\sigma_{\kappa1}}^{2}$)

**Fig. Suppl. C**: The (bias in) estimates of parameter of interest *b* (contemporaneous effect of *Xt* on *Yt*) as a function of time series length determined by (a,b) static and (c,d,e) dynamical regression models applied to simulated cross-lagged data with varying number of subjects (see legend) for the trade-off example. For each situation we considered analyses of 10 time series/subjects in the presence of among-subject covariance (see Box 1 & 2). Note that the x-axes is logarithmic and that the left and right y-axes show respectively the relative bias and absolute value of the estimate of *b* for each panel.

**
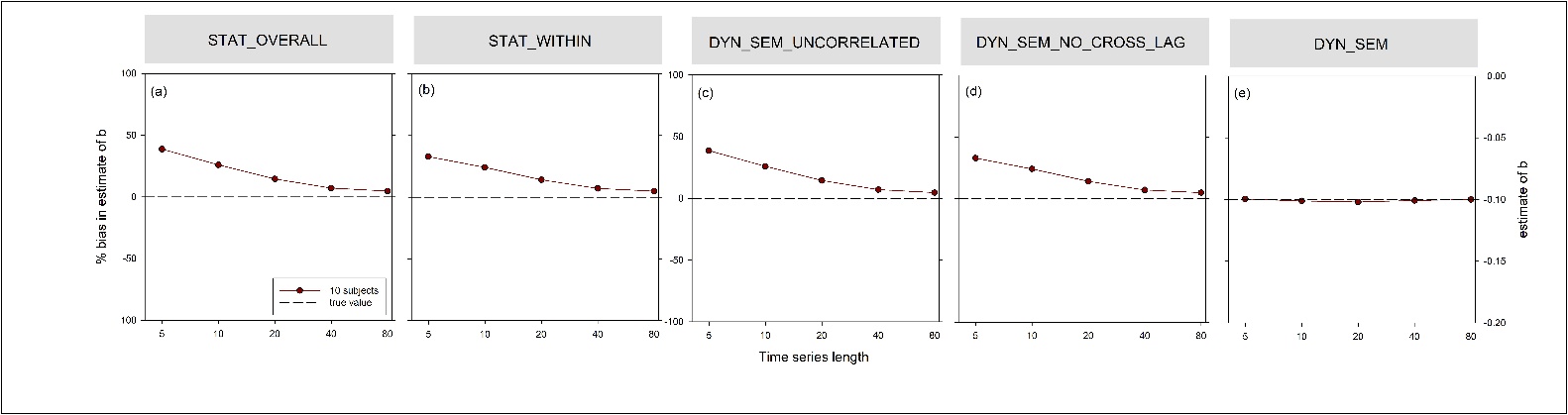
**

**Supplementary material D:**

**Sensitivity of estimation bias in single timeseries to levels of cross-lag, among-subject covariation and effect size**

***
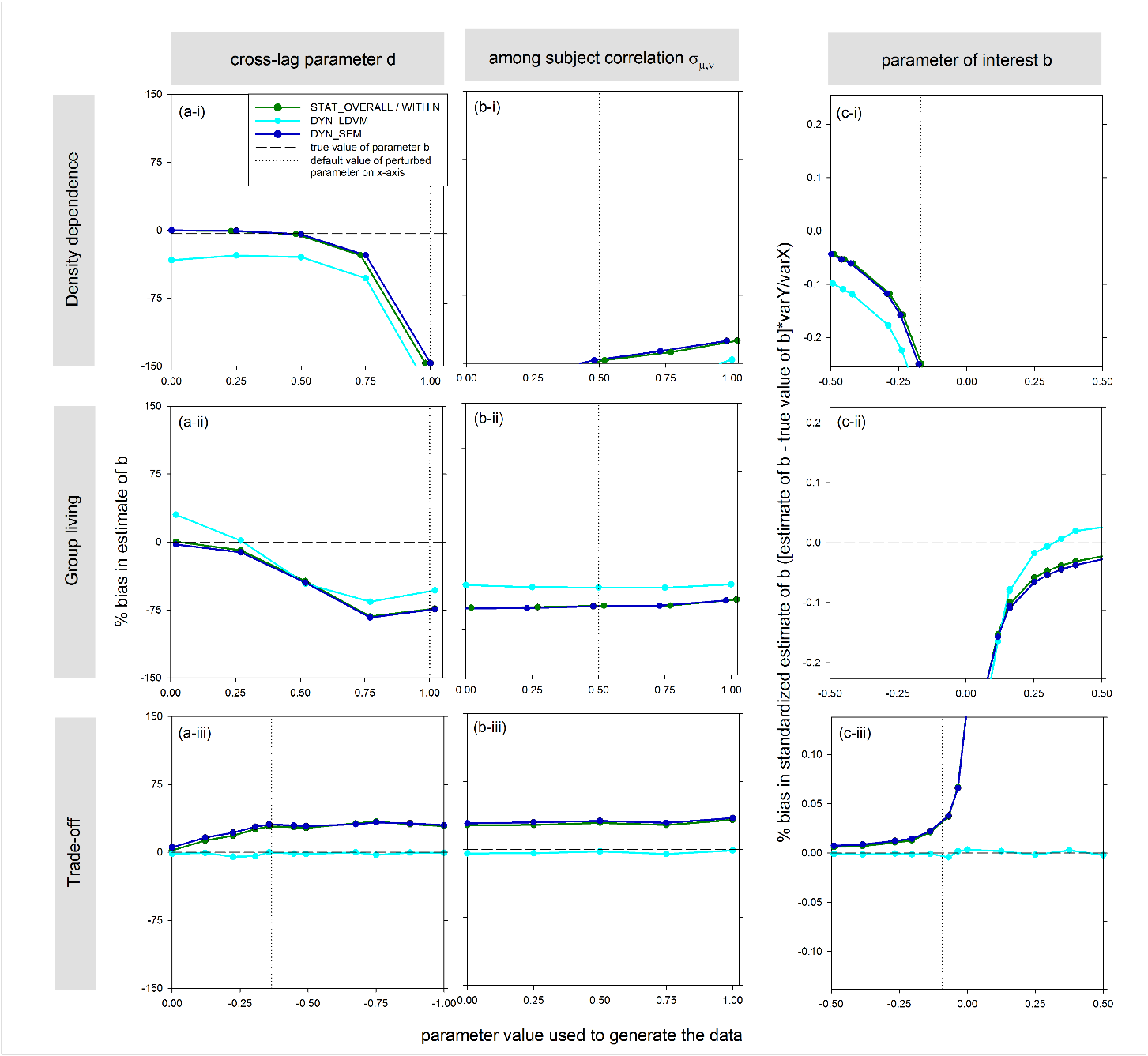
***

**Figure D:** This figure is similar to Figure 3 in the main text except that here it concerns the analysis of a single (instead of 100 subjects’) time series. The sensitivity of estimation bias in parameter of interest *b* to (a) the strength of the cross-lag, (b) the amount of among-subject covariance, and (c) the strength of the effect size (value of *b* used to generate the data), when using static and dynamic regression models (see legend) for all three examples (row panels i-iii). The vertical dotted lines show the default value of the parameters used in other simulations and figures. Note that values of parameters *b* and *d* are shown in standardized units obtained by multiplying them with varY/varX and varX/vary, respectively (except in panels a-i & a-ii, as there the absolute value of *d* can be interpreted as the probability of natal philopatry/emigration). For panels a & b we calculated the relative bias, while for panel c we present the absolute bias (as relative bias does not exist for *b*=0). Note that the dark blue and dark green lines mostly overlap and that the dark green line here represents also the STAT_WITHIN method, which becomes equivalent to STAT_OVERALL in the case of a single time series. All results are the mean estimate across 50000 simulated datasets of single subjects and 10 timesteps.

**Supplementary material E:**

**The influence of measurement error on the cross correlation between *X_t_* and *Y_t-1_***


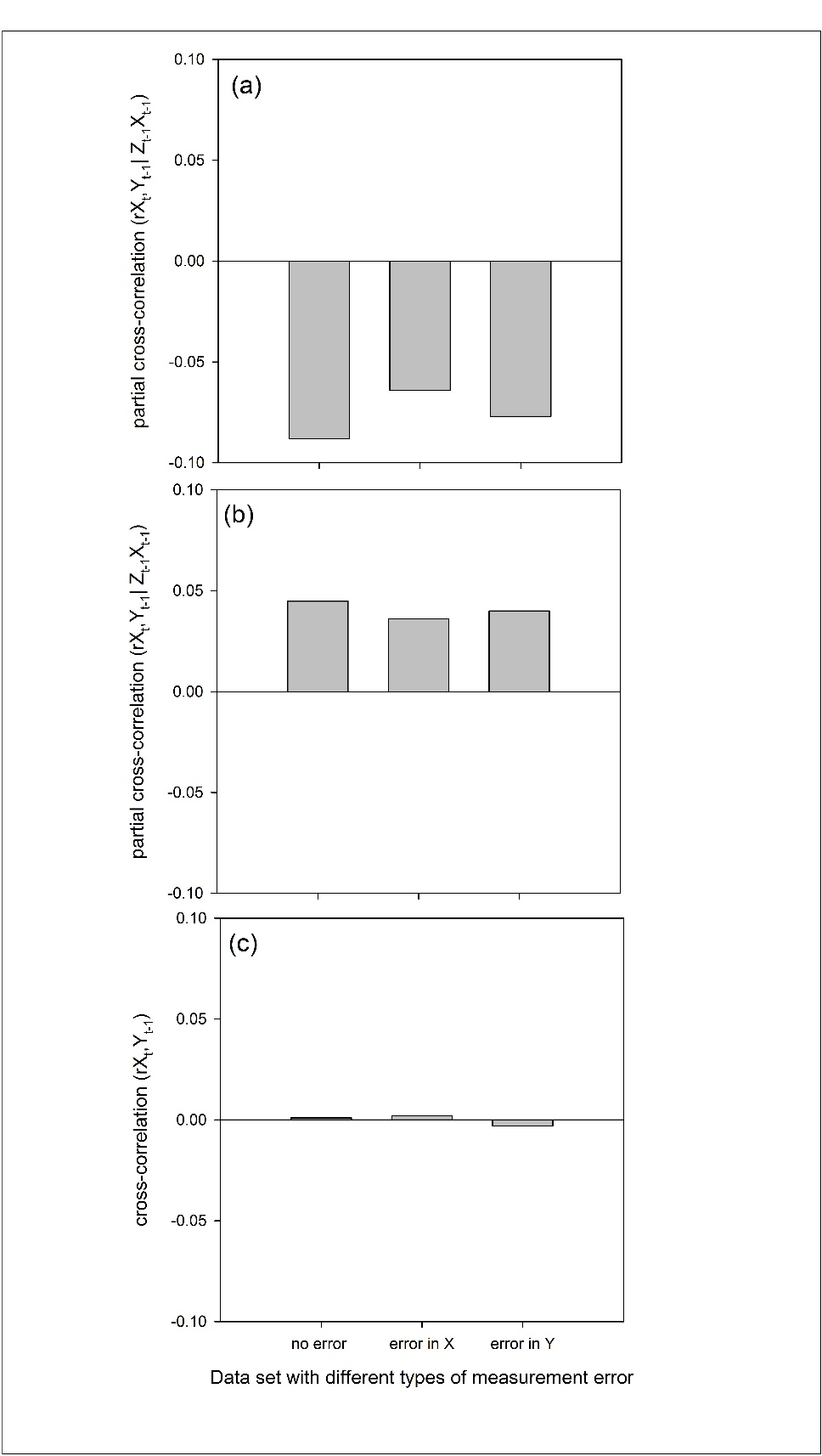
In this supplementary material we investigate whether measurement error in X or Y may cause a cross-correlation between X_t_ and Y_t-1_, even in the absence of any cross-lag in the data (i.e. without Y_t1_ affecting X_t_). We simulated datasets without cross-lag by setting parameter d=0 for each of the three examples in the main text. We subsequently calculated the cross-correlation between X_t_ and Y_t-1_ using (partial) correlation analysis for a situation with no measurement error, a situation with measurement error in X, and a situation with measurement error in Y. We used partial correlations, because X_t_ also depends on other factors (auto-lags) in the density dependence and group living example (i.e. on Z_t-1_X_t-1_; see eqs. 2 & 3 Box 1 main text).

All simulations concerned a single subject followed over 80 timesteps, for which (partial) correlations were calculated were subsequently averaged across 50,000 replicate time series. Our results show that measurement error in X or Y did not result in higher cross-correlation patterns than for the situation without any measurement error in any of the three examples (Fig. E).

***Fig. E.*** *The (partial) cross-correlation between X_t_ and Y_t-1_ in a situation without cross-lag (d=0) and either no measurement error, measurement error in X, or measurement error in Y for (a) the density dependence, (b) group living, and (c) trade-off example.*

**Supplementary material F:**

**Bias due to ignoring measurement error in cross-lagged data and how to accounting for this measurement process in dynamical models.**

Thus far, we have assumed that all variables are measured with little or no error, but in practice this is rarely true. It is well known that measurement error can cause bias in analysing auto-lagged data, which has received much attention among ecologists in the context of estimating the strength of density dependence in population size or growth from time series of population size (reviewed by Freckleton et al. 2006; Lebreton & Gimenez 2013). Furthermore, Fieberg & Didmer (2012) showed that measurement error in the predictor variable (*X*) can also influence inference from cross-lagged data in their example. In this section we will explore the influence of ignoring measurement error in both *X* and *Y* in our three simulation examples and show that DYN_SEM models are flexible in dealing with such measurement error.

For simple models with uncorrelated measurement errors among variables, theory predicts that measurement error in a predictor variable will bias estimates of *b* (the effect of *X_t_* on *Y_t_*) towards zero due to regression dilution, while measurement error in a response variable will not affect the estimation of *b* (but only affect the correlation coefficient or *R^2^*; Grace 2006). However, for more complex situations, like some of our cross-lagged multivariate examples, the effects of measurement error in *X* and *Y* are likely different and harder to predict *a priori* (Grace 2006; Fieberg & Didmer 2012).

For the analysis on potential estimation bias due to measurement uncertainty, we added measurement error to the previously described simulated data (based on eqs. 2-4 in Box 1). The values of measurement error variance were equal to 25% of the total variance in respectively X and Y, which amounts to a fairly high reliability (average correlation between measurements of 0.75). We simulated datasets with varying values of *b* based on 100 subjects followed for 10 time-steps each. The DYN_SEM+ model that was used to account for measurement errors, extended on the DYN_SEM models from Fig. Box2d*i*-*iii* by the inclusion of latent variables that describe the measurement process (Fig. F1). In the DYN_SEM+ model the amount of measurement error was assumed to be known from external sources, such as repeated measurements. See Tutorial 2 for R code for simulation and the construction of the DYN_SEM+ model in Lavaan.

Analyses of the simulated example datasets that contained measurement error confirmed that in the simplest cross-lag situation, reflecting that of a trade-off, only measurement error in *X* biased estimates of *b* towards zero (Fig. F2a), consistent with the expectation due to regression dilution. In the more complex situation of the group living example, both measurement error in *X* and *Y* caused bias (towards zero and upwards respectively, Fig. F2b), while in the situation of density dependence only measurement error in *Y* caused bias (upwards, Fig. F2c). In all above situations a dynamical error-in-variable model that specifically models the measurement error process using latent variables (DYN_SEM+), produced unbiased estimates of *b* in the presence of measurement error in *X* or *Y* (Fig. F2). Thus, in principle dynamical structural equation models are flexible enough to also deal with additional bias due to measurement error when analysing multiple time series, but they are likely to be even more data hungry (above simulation were based on relatively large sample size: n=1000).


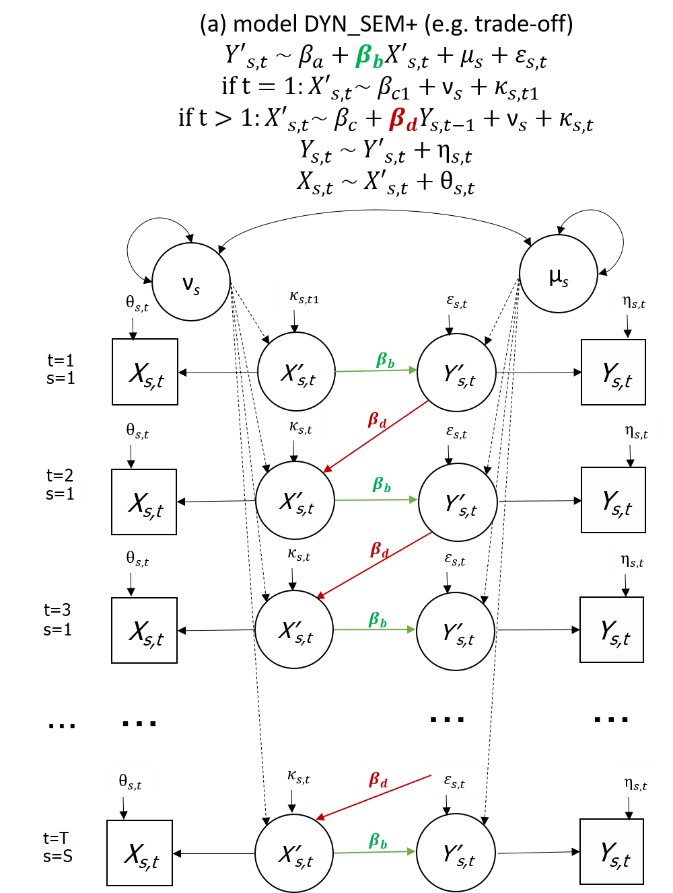


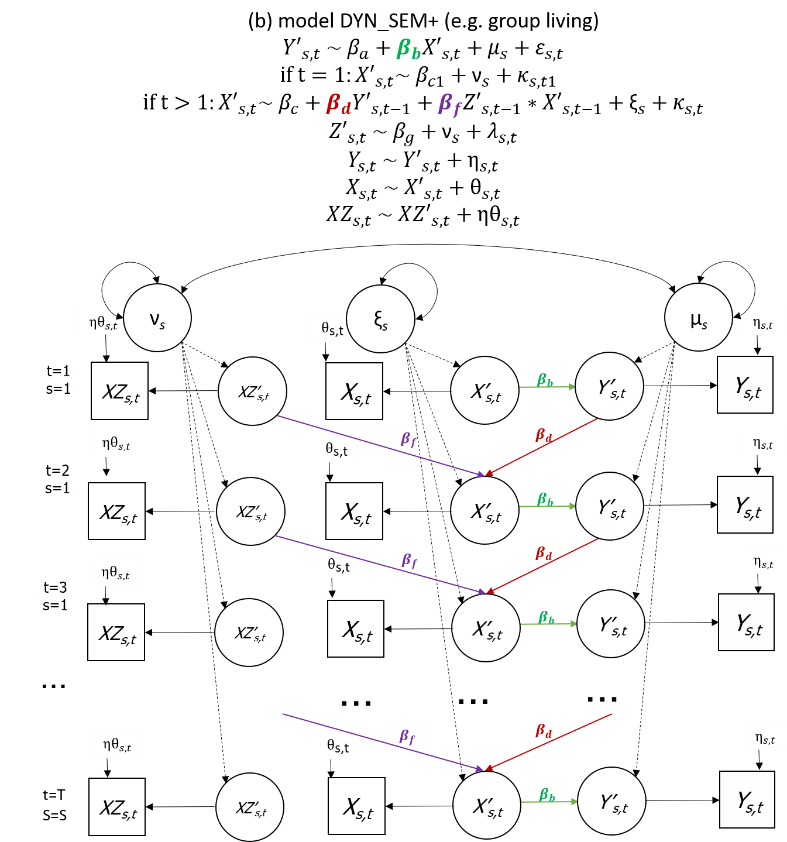


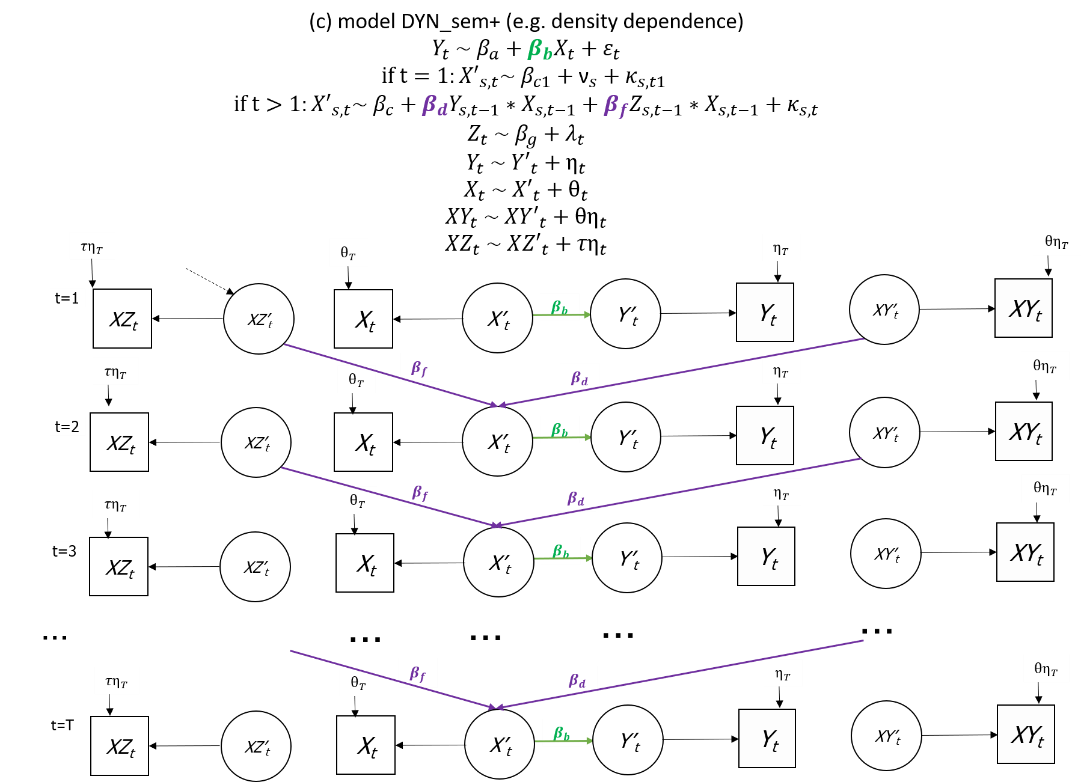


**Figure F1**: Structure of the structural equation model that accounts for measurement error in X and Y (DYN_SEM+) for a situation of (a) trade-off, (b) group living, and (c) density dependence, all with timeseries of multiple subjects. Fig F1a is also shown in the main text as Fig. Box 3-1.


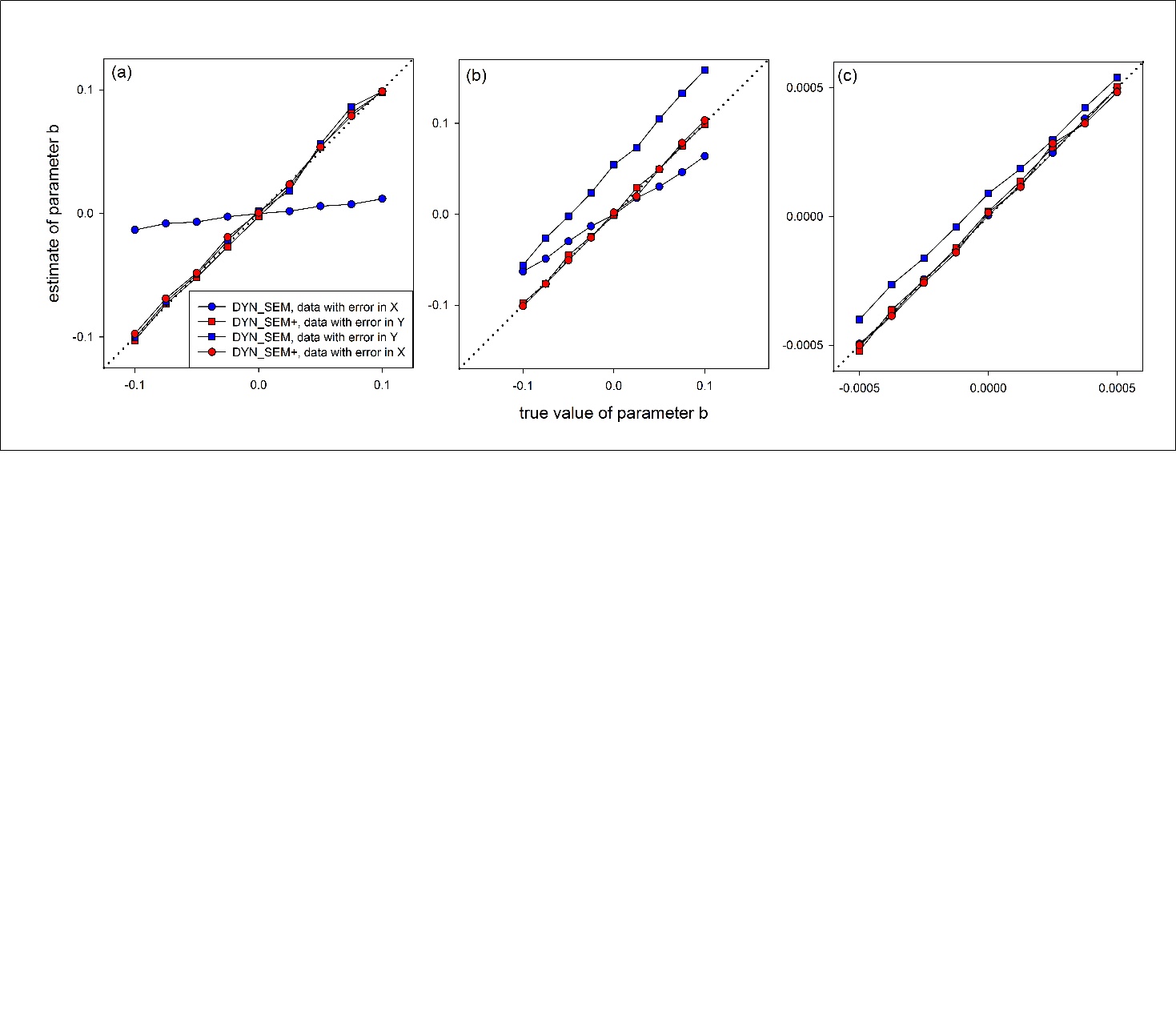


**Figure F2**: Bias in the estimate of parameter of interest *b* due to measurement error in *X* or *Y* for different true values of *b*, when using either a structural equation model that does not (DYN_SEM) or does model the measurement error process (DYN_SEM+), for the (a) trade-off, (b) group living and (c) density dependence example simulated datasets. Fig F2 is also shown in the main text as Fig. Box 3-2.

*References*

Fieberg, J. & Ditmer, M. (2012) Understanding the causes and consequences of animal movement: a cautionary note on fitting and interpreting regression models with time-dependent covariates Methods in Ecology and Evolution, 3, 983–991

Freckleton, R. P., Watkinson, A. R., Green, R. E. & Sutherland, W. J. Census error and the detection of density dependence: Census error and density dependence. *J. Anim. Ecol.* **75,** 837–851 (2006).

Grace, J. B. *Structural Equation Modeling and Natural Systems*. (Cambridge University Press, 2006).

Lebreton, J.-D. & Gimenez, O. Detecting and estimating density dependence in wildlife populations. *J. Wildl. Manag.* **77,** 12–23 (2013).
