# Supplementary material for: Cross‐lags and the unbiased estimation of life‐history and demographic parameters
Source: J Anim Ecol. 2021 Aug 18;90(10):2234–53. doi: 10.1111/1365-2656.13572 (PMC9290935; doi:10.1111/1365-2656.13572)
Supplement: Supplementary file 1 — Supplementary Material [file JANE-90-2234-s001.zip › Tutorial1.docx]

Tutorial 1: Estimation bias in simulated dataset

Martijn van de Pol & Lyanne Brouwer

7/15/2021

# Aim

In this Tutorial we show how to generate datasets of known effect size using simulation, construct static and dynamic models to analyze these datasets, and determine the bias that estimators in these models have for the contemporaneous effect of interest (parameter b in Box 1 in the main text). We will consider the three examples described in the main text on life-history trade-offs, density dependence and group living.

# Content

Structure of code document. It is divided into four sections:

1. Define the parameters and values used to generate the data for the trade-off, group living & density dependence examples.
2. Load the six different functions needed to simulate the data and run the analyses and the supporting R libraries needed in subsequent sections.
3. Run the data generating function, analyze it with different static and dynamics models and save a summary of the results to a file.
4. Explanation of the data input format and model structure used for lavaan and Stan models.

# 1. Define and set all parameter values used to generate the simulated datasets

TYPES<-c("TRADEOFF", "GROUP", "DENSDEP") # the possible data generating models
TYPE<-"TRADEOFF" # select either TRADEOFF, GROUP or DENSDEP, examples 1-3 in main text, our approach can only consider one of the three examples at a time
Subject_trials<-c(1,10,100,1000) # vector of the number of subjects that is considered in the simulted data
Timesteps_trials<-c(5,10,20,40,80) # time series length that is considered in the simulted data
SampleSize<-10 # we have set it to 10 here to speed up running the code, but in the simulations we set it to 50000
HETEROGENEITY<-TRUE # if true (default), among subject heterogeneity is included in the data generating process
MEASUREMENT_ERROR<-"NONE" # we can add measurement error on X, Y or NONE (default)
LABEL<-"outputfilename" # name of the outputfile that saves the results, by default a timestamp will be added

# parameters values used in generation of simulated datasets, see Box 1 in main text for equations, the first value is for the trade-off, the second value for the group living and third value for the density dependence example.
ParA<-c(0,55,0.55)
ParB<-c(-0.1,0.025,-0.0005) # effect of interest: effect of X on Y
ParC<-c(0,0,0)
ParD<-c(-0.5,1,1) # cross-lag: if ParD=0, then there is no cross-lag
ParG<-c(0,0.5,0.5)
ParF<-c(1,1,1)
ErrorEpsilon<-c(0.1,5,0.05) # residual noise in Y
ErrorKappa<-c(0.1,5,1.5) # residual noise in X
ErrorLambda<-c(0.1,0.1,0.05) # residual noise in Z
VarianceNu<-c(0.4,0.016,0.001) # among subject variance (heterogeneity) in X (TRADEOFF) or Z (GROUP or DENSDEP)
VarianceMu<-c(0.4,10,0.001) # among subject variance(heterogeneity) in Y
CovarMuNu<-c(0.2,0.2,0.0005) # among subject covariance
POP<-100 # initial population/group size at timestep=1 for DENSDEP and GROUP scenario
Reliability<-1 # determines measurement error, i.e. correlation between two measurements. If MEASUREMENT_ERROR!="NONE" the value is automatically set to 1 when generating the data.

# some RStan parameters (used in section 3)
StanIter<-1000 # number of iterations in Stan
StanChains<-1 # number of chains in Stan

# 2. Load seven functions to generate the data, format it in different ways, generate model structures for Lavaan and Stan, and summarize the output from analyses

There are seven functions used to perform the analysis:

1. run_sims function - generates the data, applies the static and dynamics models and calculcates their estimator bias for parameter of interest b. THis is a wrapper functions based on the six functions described below.
2. generate_data function - creates a dataset using the data generating functions described in Box 1 of the main text. It creates one dataset for a given number of replicates, subjects, time series length and the type of data structure (trade-off, group living or density dependence example)
3. lavaan_data function - transforms above data into the wide data format required by the Lavaan package for 1 replicate
4. stan_data function - transforms above data (see point a) into the list data format required by the RStan package for 1 replicate
5. lavaan_model function - produces the structure of the lavaan model, given the number of time steps, subjects and example dataset (trade-off, group living or density dependence example)
6. stan_modelfunction - produces the structure of the stan model, given the number of timesteps, subjects and example dataset
7. summarize_output function - summarizes the output of different statistical models and averages them over all replicates

We load these functions and the libraries on which they are based using:

# a list of libraries used in the analyses
library(lme4) # to run frequentist static models
library(lavaan) # to run frequentist dynamics models
library(rstan) # R package to run Bayesian models in Stan. we note that this package require some specific installation, see https://github.com/stan-dev/rstan/wiki/RStan-Getting-Started
library(shinystan) # to explore stan model output
# and various other libraries that provide useful functions to run our code
library(mvtnorm)
library(plyr)
library(gtools)
library(climwin)
library(clipr)

# next we load the seven functions described above
source("simulation_functions.R")

# 3. code that runs the functions that generate the data and models, runs the different statistical models and summarizes and stores the results on bias

We can generate simulated datasets of known effect size and determine both the absolute bias (estimate of b - true values of b, averaged over all replicates) as well as the relative bias ((estimate of b - true values of b)/true value of b), averaged over all replicates) from different methods.
Below we show how to do this for a small number of replicates (SampleSize=100). Note that in all our simulations used in the paper we set the SampleSize variable to 50000, which takes much longer to run. Furthermore, for the analysis in our paper we considered a wider range of Subjects/number of time series (Subject_trials<-c(1,10,100,1000), see Section 1) and a wider range of time series length (Timesteps_trials<-c(5,10,20,40,80)), but here we only consider a subset for illustrative purposes as otherwise it would take long to run.

Subject_trials<-c(10)
Timesteps_trials<-c(5,10)
SampleSize<-50
overview<-run_sims(STAN=FALSE, SEPARATE=FALSE)

## [1] "t5_s10_20%_TRADEOFF"
## [1] "t5_s10_40%_TRADEOFF"
## [1] "t5_s10_60%_TRADEOFF"
## [1] "t5_s10_80%_TRADEOFF"
## [1] "t5_s10_100%_TRADEOFF"
## [1] "t10_s10_20%_TRADEOFF"
## [1] "t10_s10_40%_TRADEOFF"
## [1] "t10_s10_60%_TRADEOFF"
## [1] "t10_s10_80%_TRADEOFF"
## [1] "t10_s10_100%_TRADEOFF"

print(overview)

## method median %bias mean mean_bias 2.5%
## 1 STAT_WITHIN 0.03594857 135.9 -0.001246872 98.8 -0.1259509
## 2 STAT_CROSS 0.02909257 129.1 0.014232777 114.2 -0.1093775
## 3 DYN_LDVM -0.03692532 63.1 -0.064340650 35.7 -0.4229367
## 4 DYN_SEM_LAVAAN 0.01134692 111.3 -0.025996288 74.0 -0.1449420
## 5 DYN_SEM_PLUS_LAVAAN NA NA NA NA NA
## 6 DYN_SEM_STAN NA NA NA NA NA
## 7 STAT_WITHIN_SEPARATE NA NA NA NA NA
## 8 STAT_WITHIN -0.03327547 66.7 -0.074038254 26.0 -0.1700315
## 9 STAT_CROSS -0.03051996 69.5 -0.067019216 33.0 -0.1631200
## 10 DYN_LDVM -0.10899305 -9.0 -0.123434633 -23.4 -0.2590089
## 11 DYN_SEM_LAVAAN -0.05693850 43.1 -0.097056755 2.9 -0.1875456
## 12 DYN_SEM_PLUS_LAVAAN NA NA NA NA NA
## 13 DYN_SEM_STAN NA NA NA NA NA
## 14 STAT_WITHIN_SEPARATE NA NA NA NA NA
## 25% 75% 97.5% SD SignCorr NonSign SignOppo
## 1 -0.07429099 0.038521718 0.116601687 0.10164212 0 100 0
## 2 -0.07890377 0.054791724 0.166531266 0.11597305 0 80 20
## 3 -0.08571376 0.009482067 0.227621874 0.25666538 20 80 0
## 4 -0.09982337 0.011958609 0.088038315 0.09830390 20 80 0
## 5 NA NA NA NA NA NA NA
## 6 NA NA NA NA NA NA NA
## 7 NA NA NA NA NA NA NA
## 8 -0.15947845 -0.014259932 0.005797941 0.08472146 40 60 0
## 9 -0.15612929 0.005919034 0.009169683 0.08636834 40 60 0
## 10 -0.19302665 -0.080052169 0.020109897 0.11322502 20 80 0
## 11 -0.18301476 -0.042577837 -0.017491109 0.08220069 40 60 0
## 12 NA NA NA NA NA NA NA
## 13 NA NA NA NA NA NA NA
## 14 NA NA NA NA NA NA NA
## TYPE Subjects Timesteps Replicates HETEROGENEITY MEASERROR parB parD
## 1 TRADEOFF 10 5 5 TRUE NONE -0.1 -0.5
## 2 TRADEOFF 10 5 5 TRUE NONE -0.1 -0.5
## 3 TRADEOFF 10 5 5 TRUE NONE -0.1 -0.5
## 4 TRADEOFF 10 5 5 TRUE NONE -0.1 -0.5
## 5 TRADEOFF 10 5 5 TRUE NONE -0.1 -0.5
## 6 TRADEOFF 10 5 5 TRUE NONE -0.1 -0.5
## 7 TRADEOFF 10 5 5 TRUE NONE -0.1 -0.5
## 8 TRADEOFF 10 10 5 TRUE NONE -0.1 -0.5
## 9 TRADEOFF 10 10 5 TRUE NONE -0.1 -0.5
## 10 TRADEOFF 10 10 5 TRUE NONE -0.1 -0.5
## 11 TRADEOFF 10 10 5 TRUE NONE -0.1 -0.5
## 12 TRADEOFF 10 10 5 TRUE NONE -0.1 -0.5
## 13 TRADEOFF 10 10 5 TRUE NONE -0.1 -0.5
## 14 TRADEOFF 10 10 5 TRUE NONE -0.1 -0.5
## Covar
## 1 0.2
## 2 0.2
## 3 0.2
## 4 0.2
## 5 0.2
## 6 0.2
## 7 0.2
## 8 0.2
## 9 0.2
## 10 0.2
## 11 0.2
## 12 0.2
## 13 0.2
## 14 0.2

When running the run_sims() function screenprints show what percentage of simulations has finished.

The output of this function gives us the bias of the different models applied to the dataset. Note that each method occurs twice in the first column as we supplied the function with a vector of Timesteps_trials and thus there are two levels of number of timesteps. We could also run the DYN_SEM using a Bayesian approach with Stan by setting the argument STAN=TRUE in the run_sims() function. Similarly we could also run the STAT_WITHIN_SEPERATE method mentioned in the output to analyze each time series seperately and then take the average estimate of b across all subjects, which can be seen as a two-step version of the STAT_WITHIN model (running STAT_WITHIN_SEPERATE requires putting the SEPARATE argument to TRUE in the run_sims() function). The method DYN_SEM_PLUS_LAVAAN is NA if variable MEASUREMENT_ERROR == “None”. If we set MEASUREMENT_ERROR == “X” or MEASUREMENT_ERROR == “Y” we also get the output for a DYN_SEM in lavaan that accounts for measurement error (see Tutorial 2).

Shown in the next columns is the median absolute bias, the median relative bias, the mean absolute bias, the mean relative bias and the quantiles of the bias across all replicates. The bias values shown above are not reliable, as the number of replicates was kept low in this illustration to reduce the computational time.

The columns after the bias values store whether (i) the effect of Xt on Yt (regression parameter B) was significant in the same direction as the sign of ParB (SignCorr), (ii) was statistically non-significant at the alpha=0.05 level (NonSign), or (iii) was significant in the direction opposite to the sign of ParB (SignOppo). Note that ParB contains the value of parameter B that was used to generate the data (the true value).
These three variables are useful for power-analysis, as for example the value of SignCorr helps us determine how likely it is that one will detect a statistically significant effect of Xt on Yt for a given value of parB, number of subjects, time series length and other parameter values (the value of SignCorr gives us the power (%) to detect an effect).

Finally, in the last columns of the output we see the parameter values used to generate the data.

By running these simulation for (i) different TYPE (TRADEOFF, GROUP or DENSDEP), (ii) different number of Subject_trials (subjects), (iii) different number of Timesteps_trials (timeseries length) and (iv) different parameter values for parB, parD and covarMuNu, we obtained all results shown in Fig. 2 and Fig. 3 in the main text.

# 4. Explanation of the data input format and model structure used for lavaan and Stan models.

In this section follows an explanation of what the data format and model structure looks like for both the static and dynamic models being run with either lavaan (frequentist) or Stan (Bayesian). This explanation can be useful for understanding as well as for those who would like to tweak the code to fit slightly different model structures relevant for other biological examples, however it is not needed to read this part for determining bias, as the above 3 sections already covered this. Note that the run_sims() function used in section 3 builds on some of the function used in this section 4.

For a given data generating process (i.e. TYPE=“TRADEOFF” or TYPE=“GROUP” or TYPE=“DENSDEP”, we consider all combinations of number of subjects and time steps that are given in the vectors Subject_trials and Timesteps_trials in the parameter values section. We assume readers will be familiar with the data format and model description used in static regression models that can be implemented in e.g. lme4, so will look at what the simulated datasets look like in a format that is suitable for use by lme4 or any other package for univariate regression modelling.

# we use a small number number of subjects, timesteps and replicates here to generate a small dataset to illustrate what it looks like
TYPE<-"GROUP"
subjects_TEST<-10
Timesteps_TEST<-5
Replicates_TEST<-2
datalong<-generate_data(TYPE=TYPE, subjects=subjects_TEST, Timesteps=Timesteps_TEST, Replicates=Replicates_TEST, HETEROGENEITY=HETEROGENEITY, MEASUREMENT_ERROR=MEASUREMENT_ERROR, parB=ParB[which(TYPES==TYPE)], parD=ParD[which(TYPES==TYPE)], parA=ParA[which(TYPES==TYPE)], parC=ParC[which(TYPES==TYPE)], parF=ParF[which(TYPES==TYPE)], parG=ParG[which(TYPES==TYPE)], errorEpsilon=ErrorEpsilon[which(TYPES==TYPE)], errorLambda=ErrorLambda[which(TYPES==TYPE)], errorKappa=ErrorKappa[which(TYPES==TYPE)], covarMuNu=CovarMuNu[which(TYPES==TYPE)], varianceNu=VarianceNu[which(TYPES==TYPE)], varianceMu=VarianceMu[which(TYPES==TYPE)], POP=POP)
head(datalong)

## , , 1
##
## X Y Z SubjectID timestep Xlagged Ylagged
## [1,] -0.7504727 0.2745418 1.1760908 1 1 NA NA
## [2,] 0.2820604 1.6802654 -0.9818436 1 2 -0.7504727 0.2745418
## [3,] -0.1590236 -0.9268538 -0.8922424 1 3 0.2820604 1.6802654
## [4,] -0.8820372 0.3415865 -0.3638839 1 4 -0.1590236 -0.9268538
## [5,] -0.2147088 -1.4489637 -0.2668052 1 5 -0.8820372 0.3415865
## [6,] -0.5242943 0.3751119 0.1257108 2 1 NA NA
## Zlagged YXlagged ZXlagged Xoriginal Yoriginal YX
## [1,] NA NA NA NA NA -0.2060361
## [2,] 1.1760908 -0.2060361 -0.8826241 NA NA 0.4739364
## [3,] -0.9818436 0.4739364 -0.2769392 NA NA 0.1473916
## [4,] -0.8922424 0.1473916 0.1418876 NA NA -0.3012920
## [5,] -0.3638839 -0.3012920 0.3209592 NA NA 0.3111052
## [6,] NA NA NA NA NA -0.1966690
## ZX deviationX deviationYlagged deviationY deviationYXlagged
## [1,] -0.88262406 -0.4056363 NA 0.2904265 NA
## [2,] -0.27693923 0.6268968 -0.03470800 1.6961502 -0.1544907
## [3,] 0.14188761 0.1858128 1.37101566 -0.9109690 0.5254817
## [4,] 0.32095915 -0.5372008 -1.23610351 0.3574712 0.1989370
## [5,] 0.05728542 0.1301276 0.03233674 -1.4330789 -0.2497466
## [6,] -0.06590945 -0.6484879 NA 0.3585900 NA
## deviationZXlagged meanX meanY meanZ sdX sdY sdZ
## [1,] NA 113.9083 58.54047 0.5321992 26.52773 6.902035 0.1502864
## [2,] -0.3550258 113.9083 58.54047 0.5321992 26.52773 6.902035 0.1502864
## [3,] 0.2506590 113.9083 58.54047 0.5321992 26.52773 6.902035 0.1502864
## [4,] 0.6694859 113.9083 58.54047 0.5321992 26.52773 6.902035 0.1502864
## [5,] 0.8485574 113.9083 58.54047 0.5321992 26.52773 6.902035 0.1502864
## [6,] NA 113.9083 58.54047 0.5321992 26.52773 6.902035 0.1502864
## sdXY SubjMeanX SubjMeanY SubjMeanZ
## [1,] 1.136833 -0.3448364 -0.01588476 -0.26573687
## [2,] 1.136833 -0.3448364 -0.01588476 -0.26573687
## [3,] 1.136833 -0.3448364 -0.01588476 -0.26573687
## [4,] 1.136833 -0.3448364 -0.01588476 -0.26573687
## [5,] 1.136833 -0.3448364 -0.01588476 -0.26573687
## [6,] 1.136833 0.1241936 0.01652193 0.03066043
##
## , , 2
##
## X Y Z SubjectID timestep Xlagged Ylagged
## [1,] -0.7606393 2.03321679 0.2992750 1 1 NA NA
## [2,] 0.1042874 1.03049878 1.4704328 1 2 -0.7606393 2.0332168
## [3,] 1.4983052 -0.42216488 1.3265661 1 3 0.1042874 1.0304988
## [4,] 1.9102100 0.46370297 1.9091196 1 4 1.4983052 -0.4221649
## [5,] 3.3144955 -0.05387232 1.7579180 1 5 1.9102100 0.4637030
## [6,] 0.8604240 -0.06306351 -0.3603831 2 1 NA NA
## Zlagged YXlagged ZXlagged Xoriginal Yoriginal YX ZX
## [1,] NA NA NA NA NA -1.54654467 -0.2276403
## [2,] 0.299275 -1.5465447 -0.2276403 NA NA 0.10746801 0.1533476
## [3,] 1.470433 0.1074680 0.1533476 NA NA -0.63253184 1.9876010
## [4,] 1.326566 -0.6325318 1.9876010 NA NA 0.88577005 3.6468194
## [5,] 1.909120 0.8857701 3.6468194 NA NA -0.17855955 5.8266112
## [6,] NA NA NA NA NA -0.05426136 -0.3100823
## deviationX deviationYlagged deviationY deviationYXlagged deviationZXlagged
## [1,] -1.9739711 NA 1.4229405 NA NA
## [2,] -1.1090444 1.2304769 0.4202225 -1.0100428 -1.2549903
## [3,] 0.2849735 0.2277589 -1.0324411 0.6439699 -0.8740025
## [4,] 0.6968782 -1.2249047 -0.1465733 -0.0960300 0.9602509
## [5,] 2.1011638 -0.3390369 -0.6641486 1.4222719 2.6194694
## [6,] 0.8718215 NA 0.5321302 NA NA
## meanX meanY meanZ sdX sdY sdZ sdXY
## [1,] 105.7689 57.56614 0.4702784 24.67516 6.15944 0.1784996 0.919178
## [2,] 105.7689 57.56614 0.4702784 24.67516 6.15944 0.1784996 0.919178
## [3,] 105.7689 57.56614 0.4702784 24.67516 6.15944 0.1784996 0.919178
## [4,] 105.7689 57.56614 0.4702784 24.67516 6.15944 0.1784996 0.919178
## [5,] 105.7689 57.56614 0.4702784 24.67516 6.15944 0.1784996 0.919178
## [6,] 105.7689 57.56614 0.4702784 24.67516 6.15944 0.1784996 0.919178
## SubjMeanX SubjMeanY SubjMeanZ
## [1,] 1.21333175 0.6102763 1.35266230
## [2,] 1.21333175 0.6102763 1.35266230
## [3,] 1.21333175 0.6102763 1.35266230
## [4,] 1.21333175 0.6102763 1.35266230
## [5,] 1.21333175 0.6102763 1.35266230
## [6,] -0.01139755 -0.5951937 -0.01316279

We can see that our data is stored in a three dimensional array, in which the third dimension codes for the replicate number. In the first two dimensions of the array we can see a record for each time step for each subject with the simulated variables X, Y, Z (Z is NA in the Trade-off example), the subjectIDs and timestep and a long list of derived variables. For example, derived variables labeled “lagged” are the values from the same subject one timestep earlier (and is thus missing if timestep=1). Derived variables labeled deviation are within-subject centred (e.g. devationX=X-SubjMeanX, where SubjMeanX is the mean of X for subject s). XY equals X*Y, while MeanX is the grand mean of X across all subjects and timesteps, etcetera.

## Explanation of the data format used by lavaan and Stan to model the DYN_SEM

This above dataset is in a long data format, which can be used for example by lmer() function from lme4 package. However, if we want to run the DYN_SEM model in lavaan we need to transform it to the wide format.

lavaanData<-lavaan_data(data=as.data.frame(datalong[,,1]), Timesteps=Timesteps_TEST, subjects=subjects_TEST, TYPE=TYPE)
head(lavaanData)

## y1 y2 y3 y4 y5 x1
## 1 0.27454175 1.6802654 -0.9268538 0.3415865 -1.44896369 -0.7504727
## 2 0.37511191 0.9853062 -1.1403141 -0.1685579 0.03106359 -0.5242943
## 3 -1.65244539 -0.4440028 0.5963821 -1.1747933 -0.89157219 -0.5996871
## 4 0.22042528 0.2745564 2.0675648 -0.6972738 1.44422668 -0.7127763
## 5 -0.20414406 -0.3580095 1.6588854 -0.4820446 -1.67097517 -0.9389547
## 6 -0.08146253 -0.6780725 0.1357261 0.5528319 0.06908336 -0.3735087
## x2 x3 x4 x5 z1 z2
## 1 0.2820604 -0.1590236 -0.8820372 -0.21470877 1.1760908088 -0.98184362
## 2 0.2789358 0.6342805 0.2119584 0.02008767 0.1257107822 0.37843845
## 3 -0.6384208 -0.3066959 0.3350336 -0.12996474 -0.0008053488 0.03598093
## 4 0.4636289 1.0825264 2.3280921 4.20396772 1.0077633964 0.64193680
## 5 -1.4711025 -0.4890061 -0.2771630 -0.87919166 -2.5008267524 -0.03225579
## 6 -0.2744043 -1.1218529 -0.6441487 -1.45230349 -0.4886948872 -1.97105134
## z3 z4 z5
## 1 -0.8922424 -0.3638839 -0.26680523
## 2 -0.1497123 -0.4647950 0.26366016
## 3 0.3522845 -0.1108819 0.51043555
## 4 1.6469850 2.7181995 0.97452004
## 5 -0.6774277 -1.4240358 -0.01906751
## 6 -1.2186300 -2.2644989 -1.12060124

Now each row is one subject with y1-y5 coding for the values of y in timesteps 1-5, x1-x5 coding for the values of x in timesteps 1-5, and z1-z5 coding for the values of z in timesteps 1-5. If we would have simulated more time steps, the data frame would have been wider. Also note that when running the code onselves, the generated data will differ as they are generated using random variable functions.

Lavaan uses a frequentist approach to analyzing DYN_SEM. If we want to analyzing the DYN_SEM using Bayesian approach with Stan we need yet another format.

stanData<-stan_data(data=as.data.frame(datalong[,,1]), subject=subjects_TEST) # transform output dataset into Stan format
stanData

## $subj
## [1] 1 1 1 1 1 2 2 2 2 2 3 3 3 3 3 4 4 4 4 4 5 5 5 5 5
## [26] 6 6 6 6 6 7 7 7 7 7 8 8 8 8 8 9 9 9 9 9 10 10 10 10 10
##
## $Y
## [1] 0.27454175 1.68026541 -0.92685376 0.34158649 -1.44896369 0.37511191
## [7] 0.98530616 -1.14031409 -0.16855789 0.03106359 -1.65244539 -0.44400279
## [13] 0.59638212 -1.17479334 -0.89157219 0.22042528 0.27455645 2.06756477
## [19] -0.69727377 1.44422668 -0.20414406 -0.35800950 1.65888542 -0.48204461
## [25] -1.67097517 -0.08146253 -0.67807246 0.13572614 0.55283185 0.06908336
## [31] -0.63713898 -0.08661171 -0.97889183 -1.60027551 0.48290311 -0.34507909
## [37] -1.15865861 -0.73031280 0.71400525 0.26986233 0.82025683 -0.83483173
## [43] 0.08103141 -0.93498632 -0.62321348 2.32401550 1.95142934 1.31284045
## [49] 0.27340711 1.01217660
##
## $X
## [1] -0.75047272 0.28206043 -0.15902361 -0.88203718 -0.21470877 -0.52429432
## [7] 0.27893585 0.63428050 0.21195842 0.02008767 -0.59968712 -0.63842080
## [13] -0.30669586 0.33503362 -0.12996474 -0.71277632 0.46362893 1.08252638
## [19] 2.32809212 4.20396772 -0.93895471 -1.47110250 -0.48900613 -0.27716296
## [25] -0.87919166 -0.37350873 -0.27440431 -1.12185290 -0.64414866 -1.45230349
## [31] -0.82586551 -0.47612324 -0.44585293 -0.57092731 -0.99816176 -0.26041953
## [37] 0.16347046 0.02421303 -0.53134284 -0.01685268 -0.86356191 0.77215248
## [43] 1.10107082 1.04056116 0.68043076 -0.26041953 1.41250227 0.95394569
## [49] 0.54498179 1.55534466
##
## $Z
## [1] 1.1760908088 -0.9818436177 -0.8922424106 -0.3638839265 -0.2668052287
## [6] 0.1257107822 0.3784384489 -0.1497122785 -0.4647949548 0.2636601571
## [11] -0.0008053488 0.0359809271 0.3522844807 -0.1108819244 0.5104355527
## [16] 1.0077633964 0.6419367957 1.6469849785 2.7181995191 0.9745200414
## [21] -2.5008267524 -0.0322557891 -0.6774276860 -1.4240357945 -0.0190675074
## [26] -0.4886948872 -1.9710513390 -1.2186299736 -2.2644988588 -1.1206012377
## [31] -0.3768014387 -0.4263571431 -0.3537749311 -0.1732158528 0.0195806759
## [36] 0.4922906981 0.0347033164 -0.8547850786 -0.3411407257 0.3463888722
## [41] 1.5028738773 1.3607264770 0.4785824452 0.1298833775 0.6305793704
## [46] 1.5701014552 -0.5421410368 -0.0913750483 1.5262906969 0.1836436207
##
## $N
## [1] 50
##
## $L
## [1] 5
##
## $S
## [1] 10

The StanData is in the format of a list, with elements ‘subj’, ‘X’, ‘Y’ and ‘Z’ just being the corresponding columns from the long format dataset, and N, L & S give the total sample size (N=5*10=50 here), the time series length (L=5) and number of subjects (S=10).

## Explanation of the model description format used by lavaan and Stan to model the DYN_SEM

Next we can check what the lavaan and Stan model structure looks like for the DYN_SEM. Let’s first remind us what the regression equations are for the DYN_SEM model in case of the group living example that we focus on here in our tutorial. From Box 2d-ii in the paper we can see that our DYN_SEM model aims to describe three response variables:
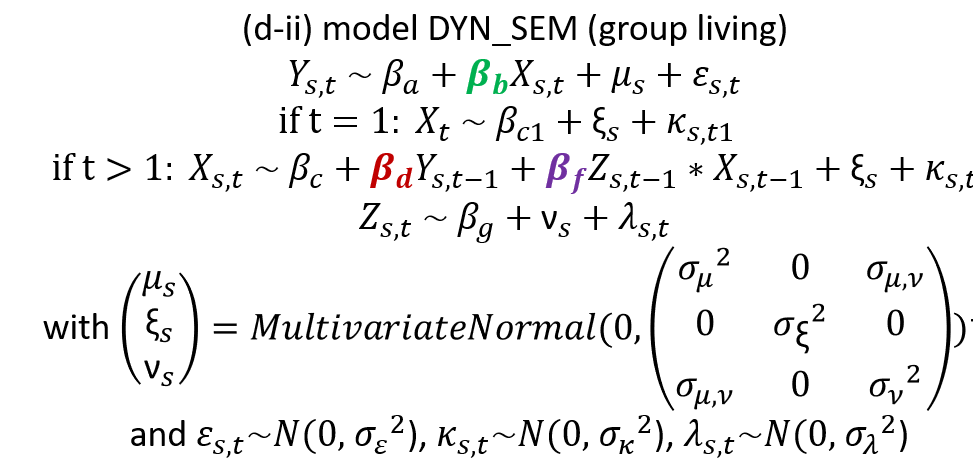


Next we can look at the code for the DYN_SEM model for group living for lavaan:

lavaanModel<-lavaan_model(TYPE, Timesteps_TEST)
print(lavaanModel)

## [1] "Mu =~1*y1 +1*y2+1*y3+1*y4+1*y5" "Nu =~1*z1 +1*z2+1*z3+1*z4+1*z5"
## [3] "Ksi =~1*x1 +1*x2+1*x3+1*x4+1*x5" "Mu~~varMu*Mu"
## [5] "Nu~~varNu*Nu" "Ksi~~varKsi*Ksi"
## [7] "Mu~~covMuNu*Nu" "Mu~~0*Ksi"
## [9] "Nu~~0*Ksi" "y1~inty*1+b*x1"
## [11] "y2~inty*1+b*x2" "y3~inty*1+b*x3"
## [13] "y4~inty*1+b*x4" "y5~inty*1+b*x5"
## [15] "x1~intx1*1" "x2~intx*1+d*y1 +f*z1:x1"
## [17] "x3~intx*1+d*y2 +f*z2:x2" "x4~intx*1+d*y3 +f*z3:x3"
## [19] "x5~intx*1+d*y4 +f*z4:x4" "z1~intz*1"
## [21] "z2~intz*1" "z3~intz*1"
## [23] "z4~intz*1" "z5~intz*1"
## [25] "y1~~vary*y1" "y2~~vary*y2"
## [27] "y3~~vary*y3" "y4~~vary*y4"
## [29] "y5~~vary*y5" "x1~~varx1*x1"
## [31] "x2~~varx*x2" "x3~~varx*x3"
## [33] "x4~~varx*x4" "x5~~varx*x5"
## [35] "z1~~varz*z1" "z2~~varz*z2"
## [37] "z3~~varz*z3" "z4~~varz*z4"
## [39] "z5~~varz*z5"

We will explain step by step how each expression in the lavaan code is linked to the DYN_SEM model described in the graphical path diagram of the model from the paper (and also shown above). In our lavaan code the first three expressions code the random subject intercepts in the model for variables Y Z and X respectively, by constructing latent variables Mu, Nu and Ksi:

Mu =~ 1 * y1 + 1 * y2 + 1 * y3 + 1 * y4 + 1 * y5

Nu =~ 1 * z1 + 1 * z2 + 1 * z3 + 1 * z4 + 1 * z5

Ksi =~ 1 * x1 + 1 * x2 + 1 * x3 + 1 * x4 + 1 * x5

These three random intercepts for subject have a variance to be estimated: varMu, varNu and varKsi, which is coded by the next three expressions:

Mu ~~ varMu * Mu

Nu ~~ varNu * Nu

Ksi ~~ varKsi * Ksi

These subject random effects may also covary, and in the next three expressions we specify that we are interested in estimating the covariance between Mu and Nu (among-subject covariance between Y and Z; reproduction and survival, e.g. due to some groups living in territories with much food, which allows them to both reproduce and survive well), but assume that group size does not covary with reproduction and survival (beyond the correlation that may exist caused by the covariance between Y and Z):

Mu ~~ covMuNu * Nu

Mu ~~ 0 * Ksi

Nu ~~ 0 * Ksi

The next five expression describe the fixed part of the reqression equation for Y, and how it depends on X via coefficient of interest b (and an intercept is also included):

y1 ~ inty * 1 + b * x1

y2 ~ inty * 1 + b * x2

y3 ~ inty * 1 + b * x3

y4 ~ inty * 1 + b * x4

y5 ~ inty * 1 + b * x5

The next five expression describe the fixed part of the reqression equation for X, and how it depends on Y via cross-lag coefficient d (and an intercept term and the auto-lag term Z*X is also included):

x1 ~ intx1 * 1

x2 ~ intx * 1 + d * y1 + f * z1 : x1

x3 ~ intx * 1 + d * y2 + f * z2 : x2

x4 ~ intx * 1 + d * y3 + f * z3 : x3

x5 ~ intx * 1 + d * y4 + f * z4 : x4

Note that in above regression equations the predictor variables are lagged (x2~y1), and this explains why for the first time step there is only an intercept, as there is no data on the predictor values in the previous time step then (note that the intercept coefficient for the first time-step (intx1) is a different regression parameter than the intercept in later timesteps (intx)!). Lavaan uses the semicolon (:) to indicate an interaction between variables (here z and x).

And for the z variable, we simply assume this only depends on an intercept:

z1 ~ intz * 1

z2 ~ intz * 1

z3 ~ intz * 1

z4 ~ intz * 1

z5 ~ intz * 1

Finally, we need to estimate the residual error terms for Y, X and Z:

y1 ~~ vary * y1

y2 ~~ vary * y2

y3 ~~ vary * y3

y4 ~~ vary * y4

y5 ~~ vary * y5

x1 ~~ varx1 * x1

x2 ~~ varx * x2

x3 ~~ varx * x3

x4 ~~ varx * x4

x5 ~~ varx * x5

z1 ~~ varz * z1

z2 ~~ varz * z2

z3 ~~ varz * z3

z4 ~~ varz * z4

z5 ~~ varz * z5

Again note that for timestep 1 for X (x1) we estimate the variance with a different parameter (varx1) than for the later timesteps (varx), as we are missing data on the lagged predictor variables for the first time step of X meaning that there will be more residual variance.

Similarly, we can look at the StanModel description and relate it to the three regression equations that we described in Fig. Box2d-ii and that we also presented earlier in this tutorial:

stanModel<-stan_model(TYPE, Timesteps_TEST, subjects_TEST)
print(stanModel)

## [1] "data {\n int<lower=1> N; //number of data points\n int<lower=1> S; //number of subjects\n int<lower=1> L; //number of timesteps\n vector [N] Y; \n vector [N] X;\n vector [N] Z; \n}\nparameters {\n vector[7] beta; //fixed intercept and slope first equation (Y~X)\n vector<lower=0>[4] sigma_e; // error sd of response vairables \n real<lower=0> sigma_v; //sd of random intercept effect for X\n vector[S] eta;\n vector<lower=0>[2] sigma_u; //among subject sd\n cholesky_factor_corr[2] L_u;\n matrix[2,S] z_u;\n}\ntransformed parameters {\n matrix[2,S] u;\n vector[S] v;\n u = diag_pre_multiply(sigma_u, L_u) * z_u; //subj random effects\n v = sigma_v * eta;\n}\nmodel {\n //priors\n sigma_e ~ normal(0,1);\n beta ~ normal(0,2);\n L_u ~ lkj_corr_cholesky(2.0);\n to_vector(z_u) ~ normal(0,1);\n eta ~ normal(0,1);\n //likelihood\n for (s in 1:S) {\n Y[(1+(s-1)*L):(L+(s-1)*L)] ~ normal(beta[1] + beta[2] * X[(1+(s-1)*L):(L+(s-1)*L)] + u[1,s], sigma_e[1]); // equation 1\n X[(2+(s-1)*L):(L+(s-1)*L)] ~ normal(beta[3] + beta[4] * Y[(1+(s-1)*L):((L-1)+(s-1)*L)]+ beta[6]* X[(1+(s-1)*L):((L-1)+(s-1)*L)].*Z[(1+(s-1)*L):((L-1)+(s-1)*L)] + v[s], sigma_e[2]); // equation 2f or t>1\n X[(1+(s-1)*L)] ~ normal(beta[5] + v[s], sigma_e[3]); // equation for t=1\n Z[(1+(s-1)*L):(L+(s-1)*L)] ~ normal(beta[7] + u[2,s], sigma_e[4]); \n }\n}\n"

We can rewrite this character string into a bit more convenient format to understand why the model looks like this:

data {
int<lower=1> N; //number of data points
int<lower=1> S; //number of subjects
int<lower=1> L; //number of timesteps
vector [N] Y;
vector [N] X;
vector [N] Z; }

This first section above describes the variables in the data (see object stanData).

parameters {
vector[7] beta; //fixed intercept and slope first equation (Y~X)
vector<lower=0>[4] sigma_e; // error sd of response variables
real<lower=0> sigma_v; //sd of random intercept effect for X
vector[S] eta;
vector<lower=0>[2] sigma_u; //among subject sd
cholesky_factor_corr[2] L_u;
matrix[2,S] z_u; }

The second section describes the parameters of the model. vector[7] beta -> describes the seven regression coefficients that are in the regression equations for X, Y and Z: we aim to estimate three parameters (b,d,f) and four intercepts (intercepts for Y,Z and X, with the intercepts for X varying between timestep 1 and later timesteps)
vector<lower=0>[4] sigma_e -> there are four error terms (for Y, Z and X, with the error term for X varying between timestep 1 and later timesteps). These error terms are constrained to be postive (lower=0). real<lower=0> sigma_v & vector[S] eta -> describe the among-subject random intercept term for X vector<lower=0>[2] sigma_u & cholesky_factor_corr[2] & L_u; matrix[2,S] z_u -> describe the among-subject random intercept term for Y and Z and their covariance

transformed parameters {
matrix[2,S] u;
vector[S] v;
u = diag_pre_multiply(sigma_u, L_u) * z_u; //subj random effects
v = sigma_v * eta; }

The third section introduces some transformed parameters that we need to obtain estimates for the (co)variances of the random subject effects that we just defined.

model {
//priors
sigma_e ~ normal(0,1);
beta ~ normal(0,2);
L_u ~ lkj_corr_cholesky(2.0);
to_vector(z_u) ~ normal(0,1);
eta ~ normal(0,1);
//likelihood
for (s in 1:S) { Y[(1+(s-1) * L):(L+(s-1) * L)] ~ normal(beta[1] + beta[2] * X[(1+(s-1) * L):(L+(s-1) * L)] + u[1,s], sigma_e[1]); // equation 1
X[(2+(s-1) * L):(L+(s-1) * L)] ~ normal(beta[3] + beta[4] * Y[(1+(s-1) * L):((L-1)+(s-1) * L)]+ beta[6] * X[(1+(s-1) * L):((L-1)+(s-1) * L)].*Z[(1+(s-1) * L):((L-1)+(s-1) * L)] + v[s], sigma_e[2]); // equation 2f or t>1
X[(1+(s-1) * L)] ~ normal(beta[5] + v[s], sigma_e[3]); // equation for t=1
Z[(1+(s-1) * L):(L+(s-1) * L)] ~ normal(beta[7] + u[2,s], sigma_e[4]); } }

The fourth model section first describes the priors used (which were set to be quite flat) for the residual error terms, the fixed effect coefficients and the among-subject (co)variance terms. Next is the description of the model likelihood functions. We use a loop to loop across all subjects in the dataset. These equations show that we assume that X, Y and Z are generated by a gaussian process (normal) and directly reflect the regression equations from Fig. Box2d-ii, but again it should be noted that for timestep 1 for each subject we do not know how X depends on Y, Z and X in the previous timestep as this data is unavailable and thus a seperate regression expression is needed for the first timestep of X. Note that in Stan-language a product of two variables is denoted by ".*".
