# Supplementary material for: Cross‐lags and the unbiased estimation of life‐history and demographic parameters
Source: J Anim Ecol. 2021 Aug 18;90(10):2234–53. doi: 10.1111/1365-2656.13572 (PMC9290935; doi:10.1111/1365-2656.13572)
Supplement: Supplementary file 1 — Supplementary Material [file JANE-90-2234-s001.zip › Tutorial2.docx]

Tutorial 2: Bias due to measurement error and how to account for it

Martijn van de Pol & Lyanne Brouwer

7/15/2021

# Aim

In this Tutorial we show how to create dynamic models that account for measurement error in either X or Y. This Tutorial builds on Tutorial 1.

# Content

Structure of code document. It is divided into four sections (sections 1-2 are the same as in Tutorial 1):

1. Define the parameters and values used to generate the data for the trade-off, group living & density dependence example
2. Load the functions needed to simulate the data and supporting R packages used by these functions
3. Run all code, but in contrast to Tutorial 1 we now add measurement error in X or Y and specifically compare the bias in models that do not to the bias in DYN_SEM models that do account for such error.
4. Explain how the DYN_SEM+ model that account for measurement error looks like.

# 1. Define and set all parameter values used to generate the simulated datasets

TYPES<-c("TRADEOFF", "GROUP", "DENSDEP") # the possible data generating models
TYPE<-"TRADEOFF" # select either TRADEOFF, GROUP or DENSDEP, examples 1-3 in main text, our approach can only consider one of the three examples at a time
Subject_trials<-100 # vector of the number of subjects that is considered in the simulted data
Timesteps_trials<-10 # time series length that is considered in the simulted data
SampleSize<-1000 # we have put it to 1000 here to speed up running the code, but in the simulations we set it to 50000
HETEROGENEITY<-TRUE # if true (default), among subject heterogeneity is included in the data generating process
MEASUREMENT_ERROR<-"X" # we can add measurement error on X, Y or NONE (default)
LABEL<-"outputfilename" # name of the outputfile that saves the results, by default a timestamp will be added

# parameters values used in generation of simulated datasets, see Box 1 in main text for equations, the first value is for the trade-off, the second value for the group living and third value for the density dependence example.
ParA<-c(0,55,0.55)
ParB<-c(-0.1,0.025,-0.0005) # effect of interest: effect of X on Y
ParC<-c(0,0,0)
ParD<-c(-0.5,1,1) # cross-lag: if ParD=0, then there is no cross-lag
ParG<-c(0,0.5,0.5)
ParF<-c(1,1,1)
ErrorEpsilon<-c(0.1,5,0.05) # residual noise in Y
ErrorKappa<-c(0.1,5,1.5) # residual noise in X
ErrorLambda<-c(0.1,0.1,0.05) # residual noise in Z
VarianceNu<-c(0.4,0.016,0.001) # among subject variance (heterogeneity) in X (TRADEOFF) or Z (GROUP or DENSDEP)
VarianceMu<-c(0.4,10,0.001) # among subject variance(heterogeneity) in Y
CovarMuNu<-c(0.2,0.2,0.0005) # among subject covariance
POP<-100 # initial population/group size at timestep=1 for DENSDEP and GROUP scenario
Reliability<-0.75 # determines measurement error, i.e. correlation between two measurements. If MEASUREMENT_ERROR!="NONE" the value is automatically set to 1 when generating the data.

# 2. Load six functions to generate the data, format it in different ways, generate model structures for Lavaan and Stan, and summarize the output from analyses

We load these functions using:

# 1. a list of libraries used in the analyses
library(rstan) # R package to run Bayesian models in Stan. we note that this package require some specific installation, see https://github.com/stan-dev/rstan/wiki/RStan-Getting-Started
library(shinystan) # to explore stan model output
library(lme4) # to run frequentist static models
library(lavaan) # to run frequentist dynamics models
# below various libraries that provided useful functions to run our code
library(mvtnorm)
library(plyr)
library(gtools)
library(climwin)
library(clipr)
source("simulation_functions.R")

# 3. Generate the data and run the different statistical models

In contrast to Tutorial 1 we now add measurement error in X or Y and specifically compare the bias in models that do not to DYN_SEM models that do account for such error (i.e. DYN_SEM_PLUS, see Box 3 in the paper).

## Generating the data and running DYN_SEM_PLUS

Below we generate some data with measurement error in X and compare the bias in estimate for parameter b for the DYN_SEM and DYN_SEM_PLUS model. We note that in the paper we have shown that for the number of subjects and timesteps that we use here (100 subjects, 10 timesteps) the DYN_SEM should produce unbiased estimates of b when there is no measurement error. The question at hand is thus whether the addition of measurement error in X introduces bias to DYN_SEM and if so whether DYN_SEM+ model is unbiased.

MEASUREMENT_ERROR<-"X"
overviewX<-run_sims(STAN=FALSE, SEPARATE=FALSE)

## [1] "t10_s100_10%_TRADEOFF"
## [1] "t10_s100_20%_TRADEOFF"
## [1] "t10_s100_30%_TRADEOFF"
## [1] "t10_s100_40%_TRADEOFF"
## [1] "t10_s100_50%_TRADEOFF"
## [1] "t10_s100_60%_TRADEOFF"
## [1] "t10_s100_70%_TRADEOFF"
## [1] "t10_s100_80%_TRADEOFF"
## [1] "t10_s100_90%_TRADEOFF"
## [1] "t10_s100_100%_TRADEOFF"

print(overviewX)

## method median %bias mean mean_bias 2.5%
## 1 STAT_WITHIN -0.02648210 73.5 -0.02571471 74.3 -0.04513480
## 2 STAT_CROSS -0.02677170 73.2 -0.02637499 73.6 -0.04570358
## 3 DYN_LDVM -0.01858372 81.4 -0.02099102 79.0 -0.04348188
## 4 DYN_SEM_LAVAAN -0.03093387 69.1 -0.03083102 69.2 -0.05087528
## 5 DYN_SEM_PLUS_LAVAAN -0.10652788 -6.5 -0.11697293 -17.0 -0.18082705
## 6 DYN_SEM_STAN NA NA NA NA NA
## 7 STAT_WITHIN_SEPARATE NA NA NA NA NA
## 25% 75% 97.5% SD SignCorr NonSign SignOppo
## 1 -0.03321025 -0.02079678 -0.002485978 0.01389005 80 20 0
## 2 -0.03336719 -0.02247901 -0.003943801 0.01348730 80 20 0
## 3 -0.02712485 -0.01645256 -0.001810495 0.01286777 30 70 0
## 4 -0.03871392 -0.02551571 -0.007704523 0.01406530 80 20 0
## 5 -0.15664125 -0.07778943 -0.072298250 0.04405620 80 20 0
## 6 NA NA NA NA NA NA NA
## 7 NA NA NA NA NA NA NA
## TYPE Subjects Timesteps Replicates HETEROGENEITY MEASERROR parB parD
## 1 TRADEOFF 100 10 10 TRUE X -0.1 -0.5
## 2 TRADEOFF 100 10 10 TRUE X -0.1 -0.5
## 3 TRADEOFF 100 10 10 TRUE X -0.1 -0.5
## 4 TRADEOFF 100 10 10 TRUE X -0.1 -0.5
## 5 TRADEOFF 100 10 10 TRUE X -0.1 -0.5
## 6 TRADEOFF 100 10 10 TRUE X -0.1 -0.5
## 7 TRADEOFF 100 10 10 TRUE X -0.1 -0.5
## Covar
## 1 0.2
## 2 0.2
## 3 0.2
## 4 0.2
## 5 0.2
## 6 0.2
## 7 0.2

From the output we can see that DYN_SEM_PLUS model is virtually unbiased (more replicates would make the estimate of bias more precise and likely closer to 0%), while the DYN_SEM model is now strongly upward biased (closer to zer0)

We can also look at the bias when b is +0.1 instead of -0.1

ParB[which(TYPES==TYPE)]<-0.1 # set value of b to +0.1 instead of -0.1
overviewX2<-run_sims(STAN=FALSE, SEPARATE=FALSE)

## [1] "t10_s100_10%_TRADEOFF"
## [1] "t10_s100_20%_TRADEOFF"
## [1] "t10_s100_30%_TRADEOFF"
## [1] "t10_s100_40%_TRADEOFF"
## [1] "t10_s100_50%_TRADEOFF"
## [1] "t10_s100_60%_TRADEOFF"
## [1] "t10_s100_70%_TRADEOFF"
## [1] "t10_s100_80%_TRADEOFF"
## [1] "t10_s100_90%_TRADEOFF"
## [1] "t10_s100_100%_TRADEOFF"

print(overviewX2)

## method median %bias mean mean_bias 2.5%
## 1 STAT_WITHIN 0.03020748 -69.8 0.02826340 -71.7 0.008922804
## 2 STAT_CROSS 0.03131557 -68.7 0.02937980 -70.6 0.010821797
## 3 DYN_LDVM 0.01032884 -89.7 0.01091088 -89.1 -0.007875866
## 4 DYN_SEM_LAVAAN 0.02454230 -75.5 0.02291286 -77.1 0.003845232
## 5 DYN_SEM_PLUS_LAVAAN 0.09276734 -7.2 0.09878949 -1.2 0.050677906
## 6 DYN_SEM_STAN NA NA NA NA NA
## 7 STAT_WITHIN_SEPARATE NA NA NA NA NA
## 25% 75% 97.5% SD SignCorr NonSign SignOppo
## 1 0.027746370 0.03489804 0.03895913 0.010160691 80 20 0
## 2 0.027767417 0.03612943 0.04043691 0.009921872 80 20 0
## 3 0.006229654 0.01946325 0.02346363 0.010578689 0 100 0
## 4 0.022279043 0.02972088 0.03368031 0.010058733 70 30 0
## 5 0.078599308 0.11855233 0.15518581 0.034161252 70 30 0
## 6 NA NA NA NA NA NA NA
## 7 NA NA NA NA NA NA NA
## TYPE Subjects Timesteps Replicates HETEROGENEITY MEASERROR parB parD
## 1 TRADEOFF 100 10 10 TRUE X 0.1 -0.5
## 2 TRADEOFF 100 10 10 TRUE X 0.1 -0.5
## 3 TRADEOFF 100 10 10 TRUE X 0.1 -0.5
## 4 TRADEOFF 100 10 10 TRUE X 0.1 -0.5
## 5 TRADEOFF 100 10 10 TRUE X 0.1 -0.5
## 6 TRADEOFF 100 10 10 TRUE X 0.1 -0.5
## 7 TRADEOFF 100 10 10 TRUE X 0.1 -0.5
## Covar
## 1 0.2
## 2 0.2
## 3 0.2
## 4 0.2
## 5 0.2
## 6 0.2
## 7 0.2

This output again shows that DYN_SEM_PLUS is virtually unbiased, but that DYN_SEM now underestimates b considerably (closer to zero). The fact that DYN_SEM produces estimates of b that are closer to zero in the presence of measurement error in X is to be expected due to regression dilution/attenuation.

Finally, we can also run the models for a situation with measurement error in Y:

MEASUREMENT_ERROR<-"Y"
SampleSize<-5000
overviewY<-run_sims(STAN=FALSE, SEPARATE=FALSE)

## [1] "t10_s100_2%_TRADEOFF"
## [1] "t10_s100_4%_TRADEOFF"
## [1] "t10_s100_6%_TRADEOFF"
## [1] "t10_s100_8%_TRADEOFF"
## [1] "t10_s100_10%_TRADEOFF"
## [1] "t10_s100_12%_TRADEOFF"
## [1] "t10_s100_14%_TRADEOFF"
## [1] "t10_s100_16%_TRADEOFF"
## [1] "t10_s100_18%_TRADEOFF"
## [1] "t10_s100_20%_TRADEOFF"
## [1] "t10_s100_22%_TRADEOFF"
## [1] "t10_s100_24%_TRADEOFF"
## [1] "t10_s100_26%_TRADEOFF"
## [1] "t10_s100_28%_TRADEOFF"
## [1] "t10_s100_30%_TRADEOFF"
## [1] "t10_s100_32%_TRADEOFF"
## [1] "t10_s100_34%_TRADEOFF"
## [1] "t10_s100_36%_TRADEOFF"
## [1] "t10_s100_38%_TRADEOFF"
## [1] "t10_s100_40%_TRADEOFF"
## [1] "t10_s100_42%_TRADEOFF"
## [1] "t10_s100_44%_TRADEOFF"
## [1] "t10_s100_46%_TRADEOFF"
## [1] "t10_s100_48%_TRADEOFF"
## [1] "t10_s100_50%_TRADEOFF"
## [1] "t10_s100_52%_TRADEOFF"
## [1] "t10_s100_54%_TRADEOFF"
## [1] "t10_s100_56%_TRADEOFF"
## [1] "t10_s100_58%_TRADEOFF"
## [1] "t10_s100_60%_TRADEOFF"
## [1] "t10_s100_62%_TRADEOFF"
## [1] "t10_s100_64%_TRADEOFF"
## [1] "t10_s100_66%_TRADEOFF"
## [1] "t10_s100_68%_TRADEOFF"
## [1] "t10_s100_70%_TRADEOFF"
## [1] "t10_s100_72%_TRADEOFF"
## [1] "t10_s100_74%_TRADEOFF"
## [1] "t10_s100_76%_TRADEOFF"
## [1] "t10_s100_78%_TRADEOFF"
## [1] "t10_s100_80%_TRADEOFF"
## [1] "t10_s100_82%_TRADEOFF"
## [1] "t10_s100_84%_TRADEOFF"
## [1] "t10_s100_86%_TRADEOFF"
## [1] "t10_s100_88%_TRADEOFF"
## [1] "t10_s100_90%_TRADEOFF"
## [1] "t10_s100_92%_TRADEOFF"
## [1] "t10_s100_94%_TRADEOFF"
## [1] "t10_s100_96%_TRADEOFF"
## [1] "t10_s100_98%_TRADEOFF"
## [1] "t10_s100_100%_TRADEOFF"

print(overviewY)

## method median %bias mean mean_bias 2.5%
## 1 STAT_WITHIN 0.11855055 18.6 0.11654482 16.5 0.002404269
## 2 STAT_CROSS 0.14154860 41.5 0.13736966 37.4 0.034062096
## 3 DYN_LDVM 0.04031724 -59.7 0.03754231 -62.5 -0.145383618
## 4 DYN_SEM_LAVAAN 0.09930895 -0.7 0.09781212 -2.2 -0.013388065
## 5 DYN_SEM_PLUS_LAVAAN 0.09876876 -1.2 0.09572001 -4.3 0.025153272
## 6 DYN_SEM_STAN NA NA NA NA NA
## 7 STAT_WITHIN_SEPARATE NA NA NA NA NA
## 25% 75% 97.5% SD SignCorr NonSign SignOppo TYPE
## 1 0.09056779 0.1419469 0.2624409 0.05885426 28 72 0 TRADEOFF
## 2 0.10550073 0.1594465 0.2504718 0.05445440 66 34 0 TRADEOFF
## 3 -0.03651778 0.1019579 0.2306902 0.10237184 4 96 0 TRADEOFF
## 4 0.06892164 0.1264086 0.2453623 0.05985500 12 88 0 TRADEOFF
## 5 0.06480018 0.1193816 0.1787981 0.04114643 12 88 0 TRADEOFF
## 6 NA NA NA NA NA NA NA TRADEOFF
## 7 NA NA NA NA NA NA NA TRADEOFF
## Subjects Timesteps Replicates HETEROGENEITY MEASERROR parB parD Covar
## 1 100 10 50 TRUE Y 0.1 -0.5 0.2
## 2 100 10 50 TRUE Y 0.1 -0.5 0.2
## 3 100 10 50 TRUE Y 0.1 -0.5 0.2
## 4 100 10 50 TRUE Y 0.1 -0.5 0.2
## 5 100 10 50 TRUE Y 0.1 -0.5 0.2
## 6 100 10 50 TRUE Y 0.1 -0.5 0.2
## 7 100 10 50 TRUE Y 0.1 -0.5 0.2

This shows that measurement error in Y does not cause bias in b, not even in the DYN_SEM.
By varying (i) whether there is measurement error in X or Y, and (ii) the value of b, we can reproduce Fig. Box 3-2a from the main text.

# 4. Explanation of the model strucuture of the DYN_SEM model in lavaan

We can look at what the lavaan model structure looks like for the DYN_SEM_PLUS model that is used to account for measurement error in X or Y. Let’s first remind us what the regression equations are for the DYN_SEM_PLUS model in case of the trade-off example that we focus on here in our tutorial. From Fig. Box 3-1 in the paper we can see that our DYN_SEM_PLUS model expands on the DYN_SEM by including two latent variables X’ and Y’:


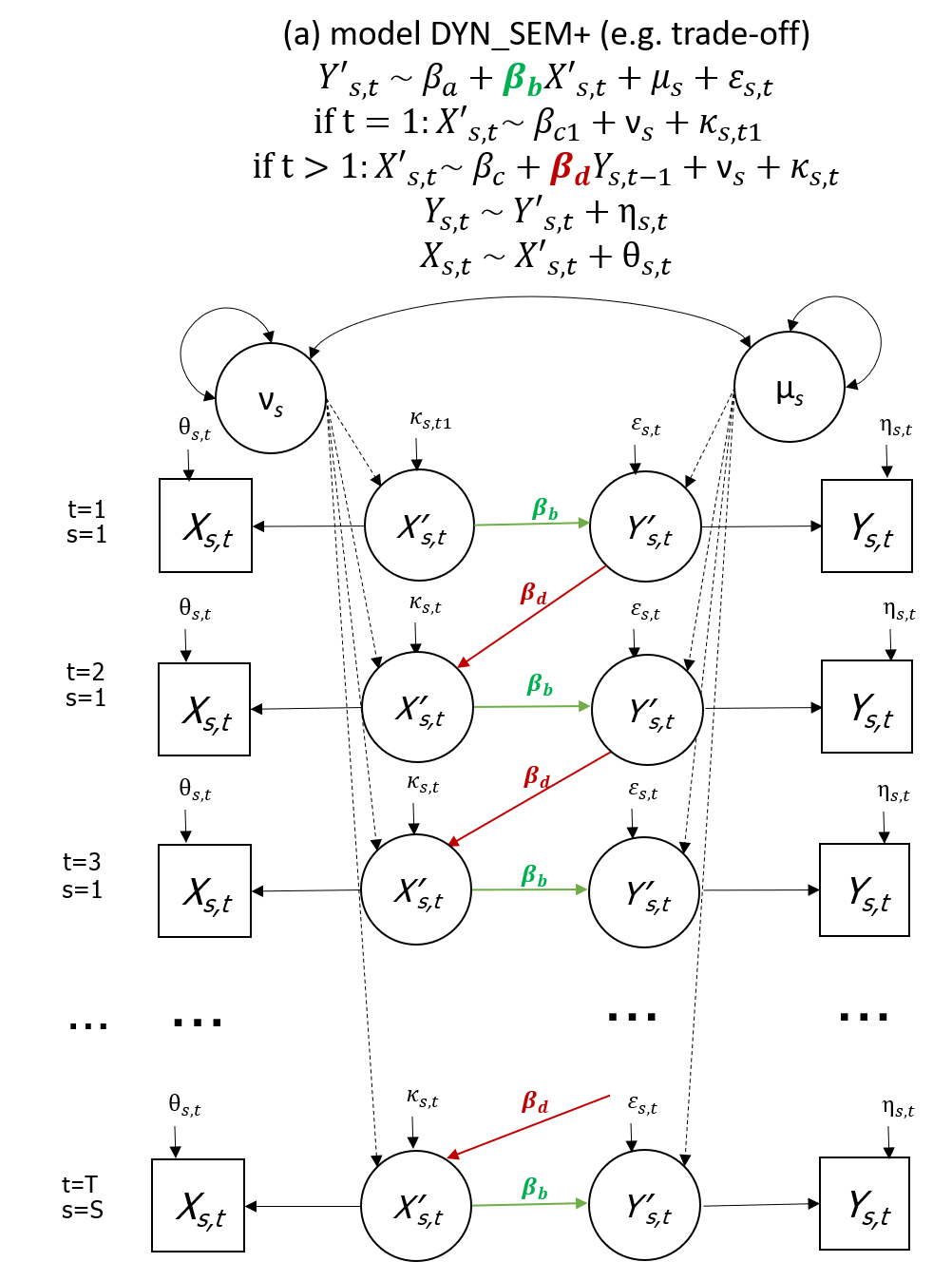


Fig. Box3-1

These two latent variables X’ and Y’ are informed by observed variables X and Y respectively. X and Y have error terms that equal their measurement error. We assume this measurement error is known from external data sources. For example, variable X in the trade-off example we consider here can be the somatic growth over a given period, defined by the change in body mass of an organism. If the organism is small it can be hard to measure their mass precisely, because scales have rounding errors, or may be affected by wind in the field. To account for such sources of measurement variation, one could conduct growth measurements twice on each occasion, and then determine the correlation between these two repeated measurements. If there is no measurement error then the correlation should be 1, but often we find that the correlation is less than one. In the dataset we have set this correlation (also called Reliability) to be 0.75. From this we can calculate the measurement error as being (1-Reliability)*variance(X). Because in our data simulations we have set the variance (X) equal to one, the measurement error is simply equal to 1-Reliability=0.25.

Next we can look at the code for the DYN_SEM_PLUS model for trade-off example for lavaan when aiming to account for measurement error in X:

lavaanModel<-lavaan_model(TYPE=TYPE, Timesteps=Timesteps_trials, ERROR=MEASUREMENT_ERROR)
print(lavaanModel)

## [1] "yL1 =~1*y1"
## [2] "yL2 =~1*y2"
## [3] "yL3 =~1*y3"
## [4] "yL4 =~1*y4"
## [5] "yL5 =~1*y5"
## [6] "yL6 =~1*y6"
## [7] "yL7 =~1*y7"
## [8] "yL8 =~1*y8"
## [9] "yL9 =~1*y9"
## [10] "yL10 =~1*y10"
## [11] "Mu =~1*yL1 +1*yL2+1*yL3+1*yL4+1*yL5+1*yL6+1*yL7+1*yL8+1*yL9+1*yL10"
## [12] "Nu =~1*x1 +1*x2+1*x3+1*x4+1*x5+1*x6+1*x7+1*x8+1*x9+1*x10"
## [13] "Mu~~varMu*Mu"
## [14] "Nu~~varNu*Nu"
## [15] "Mu~~covMuNu*Nu"
## [16] "yL1~inty*1+b*x1"
## [17] "yL2~inty*1+b*x2"
## [18] "yL3~inty*1+b*x3"
## [19] "yL4~inty*1+b*x4"
## [20] "yL5~inty*1+b*x5"
## [21] "yL6~inty*1+b*x6"
## [22] "yL7~inty*1+b*x7"
## [23] "yL8~inty*1+b*x8"
## [24] "yL9~inty*1+b*x9"
## [25] "yL10~inty*1+b*x10"
## [26] "x1~intx1*1"
## [27] "x2~intx*1+d*yL1"
## [28] "x3~intx*1+d*yL2"
## [29] "x4~intx*1+d*yL3"
## [30] "x5~intx*1+d*yL4"
## [31] "x6~intx*1+d*yL5"
## [32] "x7~intx*1+d*yL6"
## [33] "x8~intx*1+d*yL7"
## [34] "x9~intx*1+d*yL8"
## [35] "x10~intx*1+d*yL9"
## [36] "x1~~varx1*x1"
## [37] "x2~~varx*x2"
## [38] "x3~~varx*x3"
## [39] "x4~~varx*x4"
## [40] "x5~~varx*x5"
## [41] "x6~~varx*x6"
## [42] "x7~~varx*x7"
## [43] "x8~~varx*x8"
## [44] "x9~~varx*x9"
## [45] "x10~~varx*x10"
## [46] "yL1~~vary*yL1"
## [47] "yL2~~vary*yL2"
## [48] "yL3~~vary*yL3"
## [49] "yL4~~vary*yL4"
## [50] "yL5~~vary*yL5"
## [51] "yL6~~vary*yL6"
## [52] "yL7~~vary*yL7"
## [53] "yL8~~vary*yL8"
## [54] "yL9~~vary*yL9"
## [55] "yL10~~vary*yL10"
## [56] "y1~~0.250000*y1"
## [57] "y2~~0.250000*y2"
## [58] "y3~~0.250000*y3"
## [59] "y4~~0.250000*y4"
## [60] "y5~~0.250000*y5"
## [61] "y6~~0.250000*y6"
## [62] "y7~~0.250000*y7"
## [63] "y8~~0.250000*y8"
## [64] "y9~~0.250000*y9"
## [65] "y10~~0.250000*y10"

We will explain step by step how each expression in the lavaan code is linked to the DYN_SEM_PLUS model described in Fig. Box3-1 in the paper. We will first explain how to account for measurement error in X, then for measurement error in Y, which could be combined to arrive at the model shown in Fig. Box 3-1 in the paper and shown above.

In our lavaan code the first 10 expressions code for the latent variable X’, which we here coded as xL.

xL1 =~ 1 * x1

xL2 =~ 1 * x2

…

xL10 =~ 1 * x10

The next two expressions code for the random subject intercepts in the model for variables Y and X’ respectively, by constructing latent variables Mu and Nu:

Mu =~ 1 * y1 + 1 * y2 + …. + 1 * y10

Nu =~ 1 * xL1 + 1 * xL2 + …. + 1 * xL10

These two random intercepts for subject have a variance to be estimated: varMu and varNu, which is coded by the next two expressions:

Mu ~~ varMu * Mu

Nu ~~ varNu * Nu

These subject random effects may also covary, and in the next expression we specify that we are interested in estimating the covariance between Mu and Nu (among-subject covariance between Y and X; reproduction and growth, e.g. due to some individuals being better foragers allowing them to both grow and reproduce more than other individuals):

Mu ~~ covMuNu * Nu

The next ten expressions describe the fixed part of the regression equation for Y, and how it depends on X’ via coefficient of interest b (and an intercept is also included):

y1 ~ inty * 1 + b * xL1

y2 ~ inty * 1 + b * xL2

…

y10 ~ inty * 1 + b * xL10

The next ten expressions describe the fixed part of the regression equation for X’, and how it depends on Y via cross-lag coefficient d:

xL1 ~ intx1 * 1

xL2 ~ intx * 1 + d * y1

…

xL10 ~ intx * 1 + d * y10

Note that in all above expressions we have replaced x by xL, the latent variable that is informed by X.

Finally, we need to estimate the residual error terms for Y and X’:

y1 ~~ vary * y1

y2 ~~ vary * y2

…

y10 ~~ vary * y10

xL1 ~~ varx1 * xL1

xL2 ~~ varx * xL2

…

x10 ~~ varx * x10

Again note that for timestep 1 for X (xL1) we estimate the variance with a different parameter (varx1) than for the later timesteps (varx), because we are missing the data on the lagged predictor variables for the first time step of X meaning that there will be more residual variance.

The final expressions tell the model how much measurement error we think there is in X. It does so by fixing the variance of X to be equal to (1-Reliability)*variance(X), which equals 0.25 in all our data simulations.

x1 ~~ 0.25 * x1

x2 ~~ 0.25 * x2

…

x10 ~~ 0.25 * x10

If we would want to account for measurement error in Y instead of X, the DYN_SEM_PLUS model in lavaan looks like:

lavaanModel<-lavaan_model(TYPE=TYPE, Timesteps=Timesteps_trials, ERROR="Y")
print(lavaanModel)

## [1] "yL1 =~1*y1"
## [2] "yL2 =~1*y2"
## [3] "yL3 =~1*y3"
## [4] "yL4 =~1*y4"
## [5] "yL5 =~1*y5"
## [6] "yL6 =~1*y6"
## [7] "yL7 =~1*y7"
## [8] "yL8 =~1*y8"
## [9] "yL9 =~1*y9"
## [10] "yL10 =~1*y10"
## [11] "Mu =~1*yL1 +1*yL2+1*yL3+1*yL4+1*yL5+1*yL6+1*yL7+1*yL8+1*yL9+1*yL10"
## [12] "Nu =~1*x1 +1*x2+1*x3+1*x4+1*x5+1*x6+1*x7+1*x8+1*x9+1*x10"
## [13] "Mu~~varMu*Mu"
## [14] "Nu~~varNu*Nu"
## [15] "Mu~~covMuNu*Nu"
## [16] "yL1~inty*1+b*x1"
## [17] "yL2~inty*1+b*x2"
## [18] "yL3~inty*1+b*x3"
## [19] "yL4~inty*1+b*x4"
## [20] "yL5~inty*1+b*x5"
## [21] "yL6~inty*1+b*x6"
## [22] "yL7~inty*1+b*x7"
## [23] "yL8~inty*1+b*x8"
## [24] "yL9~inty*1+b*x9"
## [25] "yL10~inty*1+b*x10"
## [26] "x1~intx1*1"
## [27] "x2~intx*1+d*yL1"
## [28] "x3~intx*1+d*yL2"
## [29] "x4~intx*1+d*yL3"
## [30] "x5~intx*1+d*yL4"
## [31] "x6~intx*1+d*yL5"
## [32] "x7~intx*1+d*yL6"
## [33] "x8~intx*1+d*yL7"
## [34] "x9~intx*1+d*yL8"
## [35] "x10~intx*1+d*yL9"
## [36] "x1~~varx1*x1"
## [37] "x2~~varx*x2"
## [38] "x3~~varx*x3"
## [39] "x4~~varx*x4"
## [40] "x5~~varx*x5"
## [41] "x6~~varx*x6"
## [42] "x7~~varx*x7"
## [43] "x8~~varx*x8"
## [44] "x9~~varx*x9"
## [45] "x10~~varx*x10"
## [46] "yL1~~vary*yL1"
## [47] "yL2~~vary*yL2"
## [48] "yL3~~vary*yL3"
## [49] "yL4~~vary*yL4"
## [50] "yL5~~vary*yL5"
## [51] "yL6~~vary*yL6"
## [52] "yL7~~vary*yL7"
## [53] "yL8~~vary*yL8"
## [54] "yL9~~vary*yL9"
## [55] "yL10~~vary*yL10"
## [56] "y1~~0.250000*y1"
## [57] "y2~~0.250000*y2"
## [58] "y3~~0.250000*y3"
## [59] "y4~~0.250000*y4"
## [60] "y5~~0.250000*y5"
## [61] "y6~~0.250000*y6"
## [62] "y7~~0.250000*y7"
## [63] "y8~~0.250000*y8"
## [64] "y9~~0.250000*y9"
## [65] "y10~~0.250000*y10"

The structure is very similar to the DYN_SEM_PLUS model for measurement error in X, other than that we now have a latent variable Y’ (yL) instead of X’ (xL).
