# Supplementary material for: Cross‐lags and the unbiased estimation of life‐history and demographic parameters
Source: J Anim Ecol. 2021 Aug 18;90(10):2234–53. doi: 10.1111/1365-2656.13572 (PMC9290935; doi:10.1111/1365-2656.13572)
Supplement: Supplementary file 1 — Supplementary Material [file JANE-90-2234-s001.zip › Tutorial3.docx]

Tutorial 3: Analyzing the real-world case study of group living benefits in red-winged fairy wrens

Martijn van de Pol & Lyanne Brouwer

4/21/2021

# Aim

In this Tutorial we show how to carry out the analysis on the real world-case study of group living benefits in red-winged fairy wrens, which is described in section 7 of the paper “Cross-lags and the unbiased estimation of life-history and demographic parameters”

# Content

1. a list of libraries used in the analyses
2. the code to load and view the data
3. make some preliminary plots (incl. Fig. Box 4a from main text)
4. run and examine output from the STAT_WITHIN model
5. run and examine output from the DYN_SEM
6. plot the final results (Fig. Box 4b&c from main text)

# 1. a list of libraries used in the analyses

library(rstan) # package used to run the Bayesian version of the STAT_WTIHIN and DYN_SEM model

## Loading required package: StanHeaders

## Loading required package: ggplot2

## rstan (Version 2.21.2, GitRev: 2e1f913d3ca3)

## For execution on a local, multicore CPU with excess RAM we recommend calling
## options(mc.cores = parallel::detectCores()).
## To avoid recompilation of unchanged Stan programs, we recommend calling
## rstan_options(auto_write = TRUE)

## Do not specify '-march=native' in 'LOCAL_CPPFLAGS' or a Makevars file

library(shinystan) # package used to diagnose Bayesian output from Stan

## Loading required package: shiny

##
## This is shinystan version 2.5.0

library(lme4) # package used to run the frequentist version of the STAT_WTIHIN model

## Loading required package: Matrix

library(DHARMa) # package used for residual checks of generalized (Poisson) linear mixed models

## This is DHARMa 0.4.1. For overview type '?DHARMa'. For recent changes, type news(package = 'DHARMa') Note: Syntax of plotResiduals has changed in 0.3.0, see ?plotResiduals for details

library(climwin) # this package contains some within-subject centering functions (wgmean() and wgdev())

## Loading required package: gridExtra

library(ggplot2) # for plotting of results
# set some rstan parameters
rstan_options(auto_write = TRUE)
options(mc.cores = parallel::detectCores())

# 2.load and view the red-winged fairy wren dataset

The details of the dataset and how they were collected are described in Box 4 of the main text.

rwfw<-as.data.frame(read.table("Melegans.txt", header=T, sep="\t")) # we assumes the file is in the work-directory, the data file accompanies the paper.
rwfw[1:11,]

## SubjectID Time GroupSize Survivors Offspring LaggedUnavailable
## 1 1 1 7 6 4 1
## 2 1 2 10 8 2 0
## 3 1 3 8 6 1 0
## 4 1 4 5 3 0 0
## 5 1 5 3 3 0 0
## 6 1 6 3 2 2 0
## 7 1 7 4 4 1 0
## 8 1 8 5 3 0 0
## 9 1 9 4 3 1 0
## 10 2 1 6 4 2 1
## 11 2 2 5 4 1 0
## OffspringLagged GroupSizeLagged
## 1 NA NA
## 2 4 7
## 3 2 10
## 4 1 8
## 5 0 5
## 6 0 3
## 7 2 3
## 8 1 4
## 9 0 5
## 10 NA NA
## 11 2 6

We can see that each row contains one record for a year in which a group was monitored and data on group size, survival and offspring productivity was collected.

summary(rwfw)

## SubjectID Time GroupSize Survivors
## Min. : 1.00 Min. :1.000 Min. : 2.000 Min. :0.000
## 1st Qu.: 22.00 1st Qu.:3.000 1st Qu.: 2.000 1st Qu.:1.000
## Median : 44.00 Median :5.000 Median : 3.000 Median :2.000
## Mean : 46.04 Mean :4.929 Mean : 3.444 Mean :2.378
## 3rd Qu.: 67.00 3rd Qu.:7.000 3rd Qu.: 4.000 3rd Qu.:3.000
## Max. :108.00 Max. :9.000 Max. :10.000 Max. :8.000
##
## Offspring LaggedUnavailable OffspringLagged GroupSizeLagged
## Min. :0.0000 Min. :0.0000 Min. :0.0000 Min. : 2.000
## 1st Qu.:0.0000 1st Qu.:0.0000 1st Qu.:0.0000 1st Qu.: 3.000
## Median :0.0000 Median :0.0000 Median :0.0000 Median : 3.000
## Mean :0.5354 Mean :0.1932 Mean :0.5923 Mean : 3.563
## 3rd Qu.:1.0000 3rd Qu.:0.0000 3rd Qu.:1.0000 3rd Qu.: 4.000
## Max. :5.0000 Max. :1.0000 Max. :5.0000 Max. :10.000
## NA's :131 NA's :131

In total we have 678 records of 108 groups, with each group followed fro 1-9 years. Group size varies between 2-10. The number of offspring produced in a given year that survives to the start of the next year varies (variable “offspring”) between 0-5, and the number of adults that survives to the start of the next year (variable “Survivors”) varies between 0-8. We note that OffpsringLagged and GroupSizeLagged equal Offspring and GroupSize in the previous timestep, if such lagged data was available (note that LaggedUnavailable=1 for the first record of each subject).

# 3. evidence for the within-group cross-correlation in red-winged fairy wren data

We next explored the evidence for a withing-group cross-lag in the rwfw data

ggplot(data=rwfw[which(!is.na(rwfw$GroupSizeLagged)),], aes(x = OffspringLagged, y = GroupSize) ) +
 geom_point( position=position_jitter(h=0.2,w=0.1)) +
 scale_y_continuous(breaks = seq(0, 10, len = 6)) +
 geom_smooth(method = "lm", formula=y~x, se = FALSE) +
 labs(title=paste0("Emprical evidence for a cross-lag in the data, Pearson r=", round(cor(rwfw$OffspringLagged[which(!is.na(rwfw$GroupSizeLagged))], rwfw$GroupSize[which(!is.na(rwfw$GroupSizeLagged))],),2), ", n=",length(rwfw[which(!is.na(rwfw$GroupSizeLagged)),"GroupSize"]) ), x ="Group productivty in previous year (Yt-1)", y = "Group size in current year (Xt)")


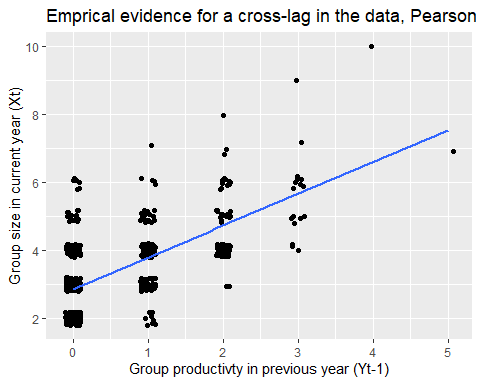


This plot reproduces Fig. Box 4a in the main text. We can see that the group size in year t strongly depends on the group productivity in the previous year, suggesting a cross-lag occurs. This cross-lag is not surprising as (i) all young of the previous year stay in their natal group for at least one year, (ii) besides the production of new group members the only other process that contributes to changes in group size is the survival of adult group members already present in the group (dispersal among groups is rare in this species).

# Explore presence of among-group covariances in group size, productivity and survival

We next explored whether there is evidence for an among-group covariance in productivity and survival. A positive covariance may be expected as some groups live in better territories than others, and more resources may mean these groups both survive and reproduce better than groups living in poorer territories. Such a postive covariance in turn may also cause a positive among-group covariance in productivity and group size. If a positive among-group covariance in productivity and group size exists, this suggest that we should focus our analysis on within-group associations between group size and productivity if we want to study the benefits of group living.

We first calculate for each group their average productivity, survival and group size across all years that they were monitored

rwfw$OffspringMean<-wgmean(rwfw$Offspring, rwfw$SubjectID) # calculates a new variable with the mean group productivty for each group
rwfw$SurvivorsMean<-wgmean(rwfw$Survivors, rwfw$SubjectID) # calculates a new variable with the mean survival for each group
rwfw$GroupSizeMean<-wgmean(rwfw$GroupSize, rwfw$SubjectID) # the wgmean function from package climwin calculates the subjects mean across all observations

Then we calculate and plot the among-group correlation in productivity and survival

ggplot(data=rwfw, aes(x = OffspringMean, y = SurvivorsMean) ) +
 geom_point() +
 geom_smooth(method = "lm", formula=y~x, se = FALSE) +
 labs(title=paste0("Emprical evidence for among-group correlation in the data, Pearson r=", round(cor(rwfw$OffspringMean,rwfw$SurvivorsMean),2), ", n=",length(unique(rwfw$SubjectID)) ), x ="Mean group productivty of a group across all years", y = "Mean number of surviving adults of a group across all years")


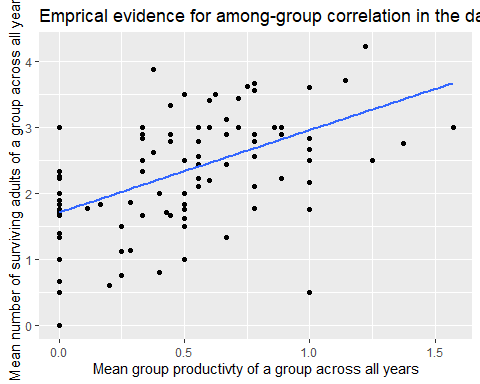


We can see there is indeed a strong positive among-group correlation between a group’s productivity and survival.

ggplot(data=rwfw, aes(x =GroupSizeMean , y = OffspringMean) ) +
 geom_point() +
 geom_smooth(method = "lm", formula=y~x, se = FALSE) +
 labs(title=paste0("Emprical evidence for a cross-lag in the data, Pearson r=", round(cor(rwfw$OffspringMean,rwfw$GroupSizeMean),2), ", n=",length(unique(rwfw$SubjectID)) ), x = "Mean group size of a group across all years", y = "Mean group productivty of a group across all years")


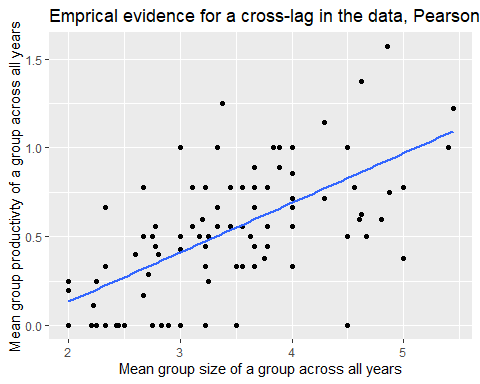


We can see in above plot that this also leads to a strong positive among-group correlation in group size and productivity. Thus large groups always produce more offspring. However, the critical question for understanding the benefits of group living is whether productivity also changes if a group changes size from year to year. Thus whether there is a within-group association between group size and productivity that is not confounded by among-group correlations between group size and productivity that are caused by heterogeneity in for example the quality of the territories these groups live in.

# 4. A static analysis of within-group association of group size and productivity.

We first run the STAT_WITHIN model. The STAT_WITHN model uses the within-subject deviation of the predictor variable, here group size. Thus, we need to calculate for each observation what the difference was between the group size and the mean group size of that group averaged over all observations. This can be done using the wgdev() function from package climwin, where SubjectID is unique for each group.

rwfw$GroupSizeDev<-wgdev(rwfw$GroupSize, rwfw$SubjectID) # the wgdev function from package climwin subtracts the subject mean from each observation (i.e. the group size - mean group size of that group across all observations)
rwfw[1:11,c(1,2,3,9,12)]

## SubjectID Time GroupSize OffspringMean GroupSizeDev
## 1 1 1 7 1.222222 1.5555556
## 2 1 2 10 1.222222 4.5555556
## 3 1 3 8 1.222222 2.5555556
## 4 1 4 5 1.222222 -0.4444444
## 5 1 5 3 1.222222 -2.4444444
## 6 1 6 3 1.222222 -2.4444444
## 7 1 7 4 1.222222 -1.4444444
## 8 1 8 5 1.222222 -0.4444444
## 9 1 9 4 1.222222 -1.4444444
## 10 2 1 6 0.600000 1.4000000
## 11 2 2 5 0.600000 0.4000000

From above view of the dataset ‘rwfw’ we can see how GroupSizeDev is calculated from the GroupSize and GroupSizeMean.

We first run the STAT_WITHIN using a frequentist mixed model with the glmer() function from package lme4 (as most readers will be familiar with using lme4), before we will run the same STAT_WITHIN model using a Bayesian approach in Rstan (a package less readers will likely be familiar with).

We assume the response variable Offspring can be described by a Poisson distribution. We added SubjectID as random intercept to the model, as we have multiple observations of the same group, and these might be correlated. Similarly, we added Time as random intercept to the model, as we have multiple observations of the same year, and these might be correlated.

STAT_WITHIN<-glmer(Offspring~GroupSizeDev+(1|SubjectID)+(1|Time), family=poisson, data=rwfw)
plot(simulateResiduals(STAT_WITHIN)) # we use the simulateResiduals function from the DHARMa


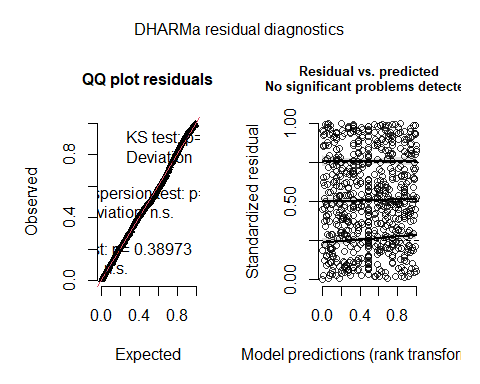


summary(STAT_WITHIN)

## Generalized linear mixed model fit by maximum likelihood (Laplace
## Approximation) [glmerMod]
## Family: poisson ( log )
## Formula: Offspring ~ GroupSizeDev + (1 | SubjectID) + (1 | Time)
## Data: rwfw
##
## AIC BIC logLik deviance df.resid
## 1341.2 1359.3 -666.6 1333.2 674
##
## Scaled residuals:
## Min 1Q Median 3Q Max
## -1.0731 -0.6951 -0.5850 0.5725 4.2378
##
## Random effects:
## Groups Name Variance Std.Dev.
## SubjectID (Intercept) 0.15663 0.3958
## Time (Intercept) 0.09326 0.3054
## Number of obs: 678, groups: SubjectID, 108; Time, 9
##
## Fixed effects:
## Estimate Std. Error z value Pr(>|z|)
## (Intercept) -0.77760 0.12811 -6.070 1.28e-09 ***
## GroupSizeDev -0.05403 0.04979 -1.085 0.278
## ---
## Signif. codes: 0 '***' 0.001 '**' 0.01 '*' 0.05 '.' 0.1 ' ' 1
##
## Correlation of Fixed Effects:
## (Intr)
## GroupSizeDv 0.020

The residual plots obtained using the DHARMa package (The ‘DHARMa’ package uses a simulation-based approach to create readily interpretable scaled (quantile) residuals for fitted generalized linear mixed models, such as mixed Poisson regression models used here) suggest that a Poisson distribution seems reasonable.

The model output next shows that group size has an effect on productivity of -0.054 (0.050S.E.) This negative estimate is on the log scale due the Poisson model, and 95% confidence intervals overlap with zero (P=0.28)

# STAT_WITHIN using a Bayesian approach.

As we will need to run the DYN_SEM using a Bayesian approach, we also will run the STAT_WITHIN model using a Bayesian approach, such that we can directly compare the posterior distribution for the effect size of interest (parameter b, the effect of group size on productivity).

We first prepare data into the correct format for RStan.

rwfwData_STAT <- list(subj = rwfw$SubjectID, ## subject identifier
 time = rwfw$Time, ## timestep identifier
 Y = rwfw$Offspring, ## the Y variable offspring productivity
 Xdev = rwfw$GroupSizeDev, ## the X variable group size, within-group centered deviation
 N = nrow(rwfw), ## number of observations
 S = length(unique(rwfw$SubjectID)), ## number of subjects
 T = length(unique(rwfw$Time))) ## number of timesteps

# Then we describe the Stan model for STAT_WITHIN.

A Stan model description has 5 sections: data, parameters, transformed parameters and model.

- The data section identifies the dimension and type of each data variable.
- The parameter section describes the regression model parameters
- the transformed parameter section can be used to calculate derived parameters from the data and parameter section
- the model section describes the priors of the regression parameters and the model’s likelihood function (based on the distribution of the regression model, here a Poisson model)
- generated quantities: an optional section that is used to do simulations from the posterior predictive distribution

Note that Stan language uses “//” for comments

BAYESIAN_STAT_WITHIN<-"data {
 int<lower=1> N; //number of data points
 int<lower=1> S; //number of subjects
 int<lower=1> T; //number of years
 int<lower=1, upper=S> subj[N]; //subject identifier
 int<lower=1, upper=T> time[N]; //timestep identifier
 int<lower=0> Y[N]; // response variable offspring productivty
 real Xdev[N]; //predictor variable within-group deviation of group size
}
parameters {
 vector[2] beta; //fixed intercept and slopes of the regression equation
 real<lower=0> sigma_u; //the among subject variability (standard deviation) in the response variable Y
 real<lower=0> sigma_v; //the among time step (i.e. year) variability (standard deviation) in the response variable Y
 vector[S] u; // a vector of subject random intercepts
 vector[T] v; // a vector of timestep random intercepts
}
transformed parameters {
 real <lower=0> m1[N]; // we calculate a new parameter m1 that equals to the regression equation
 for (n in 1:N) { // loops across all records to calculate m1
 m1[n] = exp(beta[1] + beta[2] * Xdev[n]+ u[subj[n]]+ v[time[n]]); // the regression equation, note that we use an exp() due to the log link fucntion used by a Poisson model (see next section)
 }
}
model {
 //priors
 u ~ normal(0, sigma_u); //we assume the subject random intercept is normally distributed
 v ~ normal(0, sigma_v); //we assume the year random intercept is normally distributed
 //likelihood
 Y ~ poisson(m1); // we assume a poisson model
}
generated quantities {
 // Here we do the simulations from the posterior predictive distribution
 vector[N] Y_rep ; // vector of same length as the data y
 for (n in 1:N) {
 Y_rep[n] = poisson_rng(m1[n]) ;
 }
}
"

For an explanation of why the model looks like it does above in the Stan language, please see Tutorial 1.

# run the Bayesian STAT_WITHIN

Running the Bayesian model with the next bit of code will take quite some time….

Bayesian_model_STAT_WITHIN<- stan(model_code = BAYESIAN_STAT_WITHIN, data = rwfwData_STAT, iter = 5000, chains = 4, refresh = 1000, seed=12345)
# launch_shinystan(Bayesian_model_STAT_WITHIN) # this code can be used to look at diagnostics and posteriors and opens a seperate webbrowser window.
summary(Bayesian_model_STAT_WITHIN)$summary["beta[2]",]

## mean se_mean sd 2.5% 25%
## -5.693693e-02 4.736768e-04 5.080618e-02 -1.573666e-01 -9.053749e-02
## 50% 75% 97.5% n_eff Rhat
## -5.646489e-02 -2.293117e-02 4.222729e-02 1.150453e+04 9.999856e-01

posteriors_STAT_WITHIN<-extract(Bayesian_model_STAT_WITHIN, permuted = TRUE)$beta[,2]
hist(posteriors_STAT_WITHIN) # plots the posterior distribution for parameter b


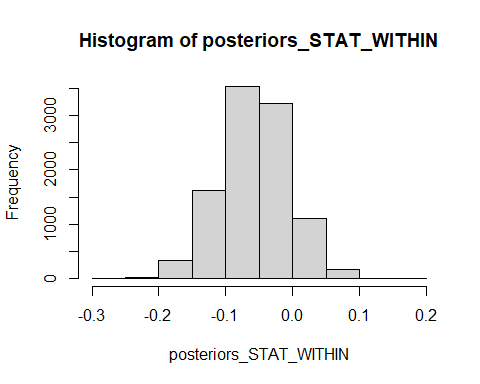


The model output gives the summary statstics (mean and a measure of precision (sd)) of the posterior distribution, as well as the 95% credible intervals. We can see that the estimate for the Bayesian version of STAT_WITHIN is very similar to the previous frequentist version of STAT_WITHIN, the estimate of b is -0.057 (+-0.051SE), compared to lme4 estimate of -0.054 (0.050.E.). The histogram gives the posterior distribution of the estimate for parameter of interest b, from which we can see that the 95% credible intervals overlap with zero. Thus, this model suggests that there are costs of group living in terms of reduced productivity, but that we cannot be very confident that they are different from zero.

To determine what this means biologically, we can plot the effect of group size on offspring productivity

GS<-seq(min(rwfw$GroupSize),max(rwfw$GroupSize),1) # create vector of observed group sizes
meanGS<-mean(rwfw$GroupSize) # calculate the mean group size in the population
# next we generate 1000 predicted values for different levels of group deviations (random intercept for SUbjectID) and different levels of year deviations (random intercept of time)
predicted_RS<-matrix(data=NA, nrow=length(GS), ncol=1000)
for(i in 1:1000) {
 predicted_RS[,i]<-exp(summary(Bayesian_model_STAT_WITHIN)$summary["beta[1]",1]+summary(Bayesian_model_STAT_WITHIN)$summary["beta[2]",1]*(GS-meanGS)+rnorm(1,0,summary(Bayesian_model_STAT_WITHIN)$summary["sigma_u",1])+rnorm(1,0,summary(Bayesian_model_STAT_WITHIN)$summary["sigma_v",1]))
}
plot(GS,rowMeans(predicted_RS), ylab="offspring productvity", xlab="group size", type = "l")


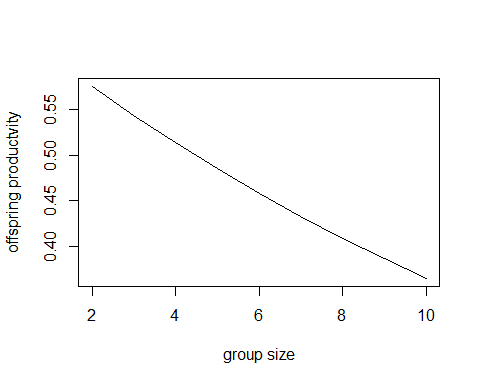


From this plot we can see that the negative effect size of b implies that the largest groups (group size 10) produce almost half the number of offspring annually (0.38 offspring) compared to the smallest groups (group size 2, produced 0.60 offspring).

# 5. run and examine output from the DYN_SEM

We next run the DYN_SEM model. Note that in contrast to the STAT_WITHIN , here we do not provide a frequentist model version but the Bayesian version using Rstan only. The frequentist version of the Lavaan model used in the simulation study is not easily extendable to non-normally distributed response variables. In the multivariate DYN_SEM model we have three response variables: group productivity (Poisson), group size (Poisson) and survival (Binomial).

First, we prepare the data into the correct format for RStan (for an explanation see Tutorial 1):

rwfwData <- list(subj = rwfw$SubjectID, ## subject identifier
 time = rwfw$Time, ## timestep identifier
 Y = rwfw$Offspring, ## the Y variable offspring productivity
 X = rwfw$GroupSize, ## the X variable offspring productivity
 Z = rwfw$Survivors, ## the Z variable offspring productivity
 L = rwfw$LaggedUnavailable, ## variable indicating whether for this record a previous group record exists. This variable is needed as we will model lags, and for the first timestep the lagged variables are unavailable.
 N = nrow(rwfw), ## number of observations
 S = length(unique(rwfw$SubjectID)), ## number of subjects
 T = length(unique(rwfw$Time))) ## number of timesteps

Next, we describe that Stan model for DYN_SEM. This model description has again 5 sections: data, parameters, transformed parameters, model and generated quantities.

- The data section identified the dimenstion and type of each data variable.
- The parameter section describes the regression model parameters
- the transformed parameter section can be used to calculate derived parameters from the data and parameter section
- the model section describes the priors of the regression parameters and the model likelihood function (based on the distribution of the regression model, here a multivariate model with two Poisson and a binomial model)
- generated quantities: an optional section that is used to do simulations from the posterior predictive distribution

BAYESIAN_DYN_SEM<-"data {
 int<lower=1> N; //number of data points
 int<lower=1> S; //number of subjects
 int<lower=1> T; //number of years
 int<lower=1, upper=S> subj[N]; //subject id
 int<lower=1, upper=T> time[N]; //timestep id
 int<lower=0> Y[N]; // variable offspring productivty
 int<lower=0> X[N]; // variable group size
 int<lower=0> Z[N]; // variable group survival
 int<lower=0, upper=1> L[N]; // indicator variable to denote whether it is the first (in time) record of subject
}
parameters {
 vector[7] beta; //fixed intercept and slopes equations 1-3. see transformed paramter section for interpretation
 real<lower=0> sigma_v; //the among subject variability (standard deviation) in variable X
 vector[S] eta; // needed for the among subject variance in X
 vector<lower=0>[2] sigma_u; // needed for the among subject variance-covariance matrix
 vector<lower=0>[3] sigma_w; // needed for the among year variance-covariance matrix
 cholesky_factor_corr[2] L_u; // needed for the above among subject variance-covariance matrix
 cholesky_factor_corr[3] L_w; // needed for the above among year variance-covariance matrix
 matrix[2,S] z_u; //a vector of subject random intercepts
 matrix[3,T] z_w; //a vector of timestep random intercepts
}
transformed parameters {
 real <lower=0> m1[N];// mu for variable y (offspring number)
 real <lower=0> m2[N]; // mu for variable x (group size)
 real <lower=0, upper=1> m3[N]; // mu for variable z (survival)
 vector[S] v;
 matrix[2,S] u;
 matrix[3,T] w;
 v = sigma_v * eta;
 u = diag_pre_multiply(sigma_u, L_u) * z_u; //needed for subj random effects
 w = diag_pre_multiply(sigma_w, L_w) * z_w; //needed for timestep random effects
 for (n in 1:N) { // loops across all records
 m1[n] = exp(beta[1] + beta[2] * X[n] + u[1,subj[n]]+ w[1,time[n]]); // y model
 m3[n] = inv_logit(beta[7] + u[2,subj[n]]+ w[2,time[n]]); // Z model
 if (L[n]==1)
 m2[n] = exp(beta[3] + v[subj[n]] + w[3,time[n]]); // X model for records where lagged variable is missing, e.g for first record of Subject
 else
 m2[n] = exp(beta[4] + beta[5] * Y[n-1] + beta[6] * Z[n-1] + v[subj[n]]+ w[3,time[n]]); // X model for records where lagged variable is available
 }
}
model {
 //priors
 eta ~ normal(0,1);
 L_u ~ lkj_corr_cholesky(2.0);
 to_vector(z_u) ~ normal(0,1);
 L_w ~ lkj_corr_cholesky(2.0);
 to_vector(z_w) ~ normal(0,1);
 //likelihood
 Y ~ poisson(m1); // equation 1, Y
 X ~ poisson(m2); // equation 2, X
 Z ~ binomial(X,m3); // equation 3, Z
}
generated quantities {
 // Here we do the simulations from the posterior predictive distribution
 vector[N] Y_rep; // vector of same length as the data y
 for (n in 1:N)
 Y_rep[n] = poisson_rng(m1[n]);
}
"

For an explanation of why the model looks like it does above in the Stan language, please see Tutorial 1.

Then we run the DYN_SEM model, which can take substantial amounts of time (~5-10 minutes)

Bayesian_model_DYN_SEM<- stan(model_code = BAYESIAN_DYN_SEM, data = rwfwData, iter = 5000, chains = 4, refresh = 1000, control = list(adapt_delta = 0.99), seed=12345)
#launch_shinystan(Bayesian_model_DYN_SEM) # this code can be used to look at diagnostics and posteriors
summary(Bayesian_model_DYN_SEM)$summary["beta[2]",]

## mean se_mean sd 2.5% 25% 50%
## 1.057629e-01 6.443344e-04 4.582445e-02 1.465197e-02 7.543493e-02 1.061832e-01
## 75% 97.5% n_eff Rhat
## 1.364653e-01 1.952025e-01 5.057918e+03 9.996499e-01

posteriors_DYN_SEM<-extract(Bayesian_model_DYN_SEM, permuted = TRUE)$beta[,2]
hist(posteriors_DYN_SEM)


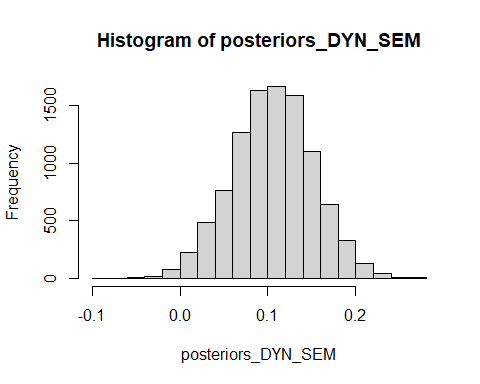


We can see that the estimate of b (beta[2]) for DYN_SEM is positive and 95% credible intervals do not overlap with zero (i.e. 0.11 (0.05S.E.), 95% CI [0.01-0.20]). Furthermore, the DYN_SEM estimate is very different (and opposite sign) from the negative estimate of STAT_WITHIN (-0.057 (0.051S.E))

To determine what this means biologically, we can plot the effect of group size on offspring productivity

# next we generate 1000 predicted values for different levels of group deviations (random intercept for SUbjectID) and different levels of year deviations (random intercept of time)
predicted_RS_DYN<-matrix(data=NA, nrow=length(GS), ncol=1000)
for(i in 1:1000) {
 predicted_RS_DYN[,i]<-exp(summary(Bayesian_model_DYN_SEM)$summary["beta[1]",1]+summary(Bayesian_model_DYN_SEM)$summary["beta[2]",1]*GS+rnorm(1,0,summary(Bayesian_model_DYN_SEM)$summary["sigma_u[1]",1])+rnorm(1,0,summary(Bayesian_model_DYN_SEM)$summary["sigma_w[1]",1]))
}
plot(GS,rowMeans(predicted_RS_DYN), ylab="offspring productvity", xlab="group size", type = "l")


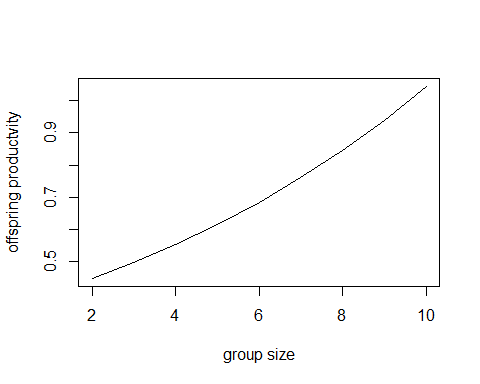


From this plot we can see that the positive effect size of b implies that the largest groups (group size 10) produce more than double the number of offspring annually (1.06 offspring) compared to the smallest groups (group size 2, produced 0.45 offspring).

# 6. Plot the final results (Fig. Box 4b&c from main text)

We can create Fig. Box 4b in the main text by plotting the posteriors of parameter of interest b for both the STAT_WITHIN and the DYN_SEM model in the same plot.

dat <- data.frame(x = c(posteriors_STAT_WITHIN, posteriors_DYN_SEM ) ,y = c(rep("Stat",10000),rep("DYN",10000)))
ggplot(dat, aes(x = x, fill = y)) + # Draw overlaying histogram
 geom_histogram(position = "identity", alpha = 0.2, bins = 50)


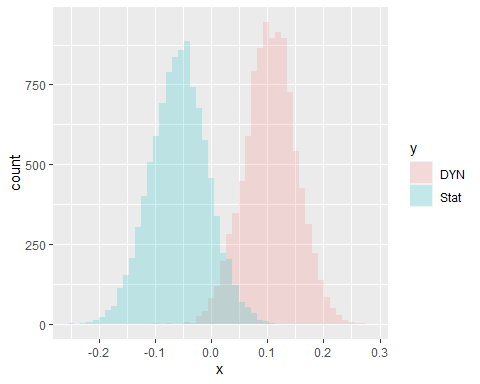


We can see that although the posterior distribution overlap somewhat, that the mean or median of the DYN_SEM posterior distribution does not overlap with the STAT_WITHIN distribution (and vice versa). Note that the effect of group size on group offspring productivity is on the log scale, as this is a regression coefficient of a Poisson regression.

We can also combine the effect size on the normal scale for both models to reproduce Fig. Box 4c in the main text.

dat2 <- data.frame(x =c(GS,GS), y = c(rowMeans(predicted_RS_DYN), rowMeans(predicted_RS)), z=c(rep("DYN",length(GS)), rep("STAT", length(GS))))
ggplot(data = dat2, aes(x = x, y = y, colour = z)) + geom_line() +
 labs(title="Effect of group size on offspring production in red-winged fairy wrens", x ="Group size in current year (Xt)", y = "Offspring productivty in current year (Yt)")


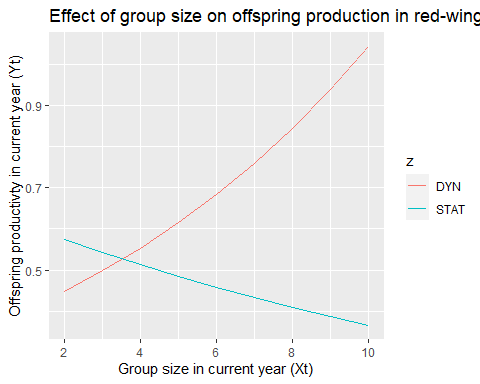


This plot emphasizes the different biological conclusions that could be drawn from applying either a static or dynamical regression model on the benefits of group living.
