# Supplementary figures and images for: Cross‐lags and the unbiased estimation of life‐history and demographic parameters
Source: J Anim Ecol. 2021 Aug 18;90(10):2234–53. doi: 10.1111/1365-2656.13572 (PMC9290935; doi:10.1111/1365-2656.13572)

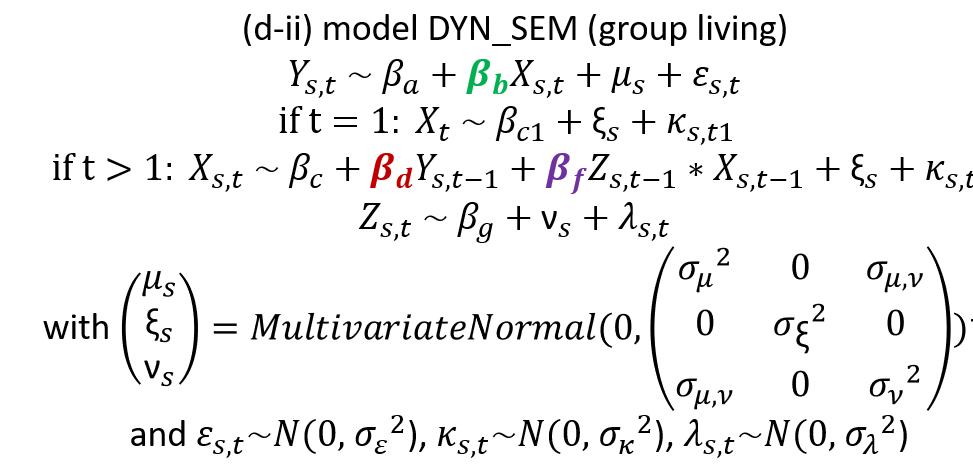

Supplement: Supplementary file 1 — Supplementary Material [file JANE-90-2234-s001.zip › Fig_tutorial1.png.png]

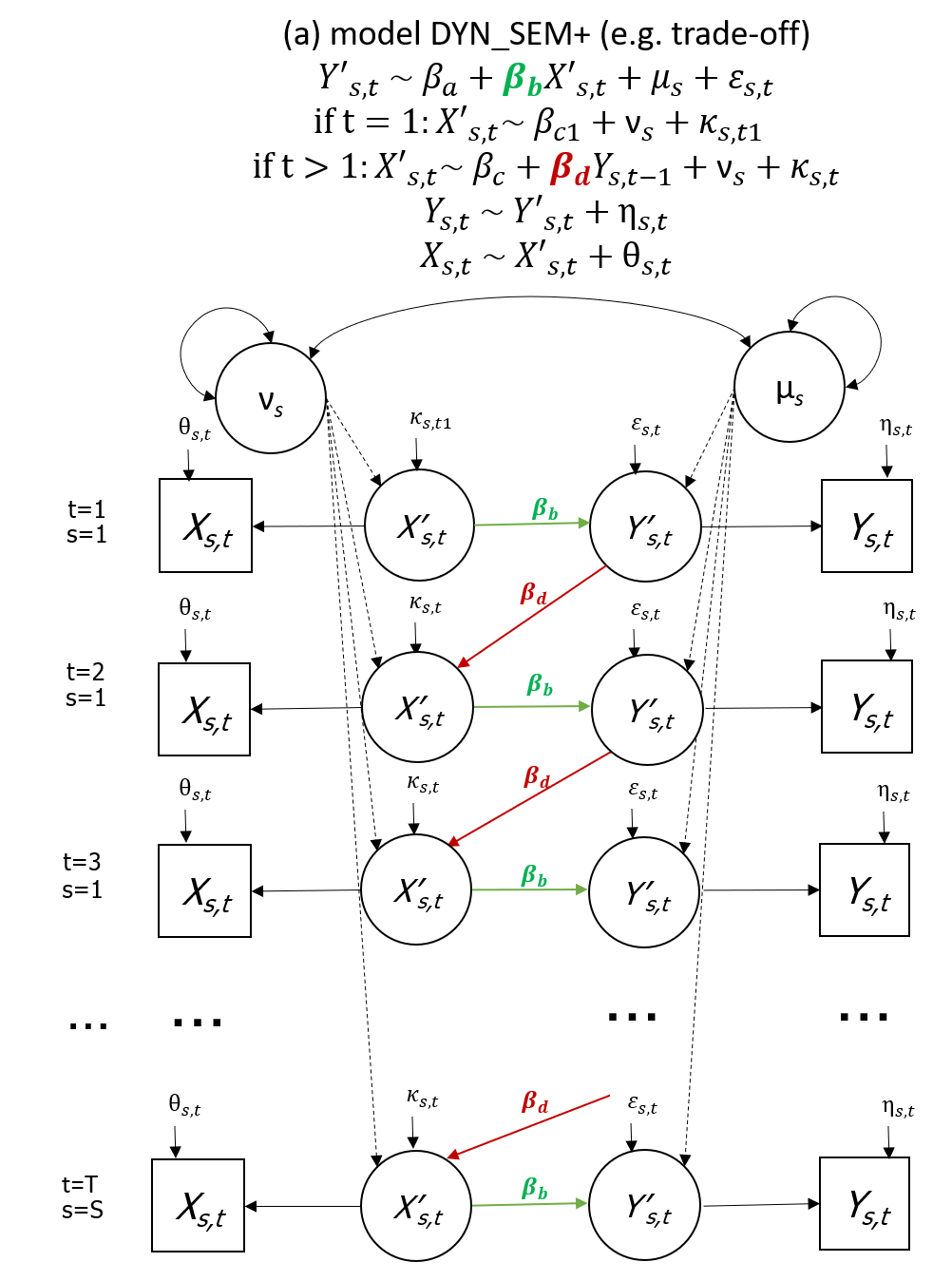

Supplement: Supplementary file 1 — Supplementary Material [file JANE-90-2234-s001.zip › Fig_tutorial2.png.png]
